# Iron Carbonyl Complexes of [2.2.2]Hericene as a Rigid Tris(1,3-diene) Ligand

**DOI:** 10.3390/molecules29225337

**Published:** 2024-11-13

**Authors:** Jinfeng Luo, Haoyu Chen, Huidong Li, Yongxiang Zheng, Qunchao Fan, Robert Bruce King, Henry F. Schaefer

**Affiliations:** 1 School of Science, Key Laboratory of High Performance Scientific Computation, Xihua University, Chengdu 610039, China; luojinfengxhu@163.com (J.L.); chenhaoyuxhu@163.com (H.C.); yongxz@xhu.edu.cn (Y.Z.); fanqunchao@sina.com (Q.F.); 2 Center for Computational Quantum Chemistry, University of Georgia, Athens, GA 30602, USA; ccq@uga.edu

**Keywords:** Hericenen, iron carbonyl compounds, density functional methods

## Abstract

Hericene is an unusual hexaolefin consisting of three 1,3-diene units located on a rigid bicyclo [2.2.2]octane framework that restricts the geometrical relationships of metal atoms bonded to these olefinic units. In order to explore possible effects of this rigidity limiting metal–metal interaction in polynuclear derivatives possibly stabilizing coordinatively unsaturated species, the structures and energetics of the hericene iron carbonyl complexes (hericene)Fe*_m_*(CO)*_n_* (*m* = 1, *n* = 3; *m* = 2, *n* = 6, 5; *m* = 3, *n* = 9, 8) have been investigated by density functional theory. The lowest-energy (hericene)Fe*_m_*(CO)_3*m*_ (*m* = 1, 2, 3) structures have the cavities of the hericene ligand filled with a single Fe(CO)_3_ moiety bonded to a 1,3-diolefin unit. Such species have been synthesized by the reaction of Fe_2_(CO)_9_ with hericene. For the (hericene)Fe_2_(CO)_5_ system, the lowest energy structures are singlet structures with an Fe(CO)_3_ unit bonded to a 1,3-diene unit in one hericene cavity and an Fe(CO)_2_ unit in another hericene cavity bonded to three C=C double bonds from two 1,3-diene units. Higher energy (hericene)Fe_2_(CO)_5_ structures include a structure in which a single hericene cavity contains a Fe_2_(CO)_4_(µ-CO) moiety with each iron atom bonded to a 1,3-diene unit. In addition, both singlet and triplet (hericene)Fe_2_(CO)_5_ structures are found in which an Fe(CO)_3_ moiety and an Fe(CO)_2_ moiety located in separate hericene cavities are each bonded to a 1,3-diene unit. The lowest-energy (hericene)Fe_3_(CO)_8_ structures have two hericene cavities containing Fe(CO)_3_ moieties fully bonded to 1,3-diene units and a third hericene cavity containing an Fe(CO)_2_ moiety fully bonded to a 1,3-diene unit.

## 1. Introduction

**Figure 1:**
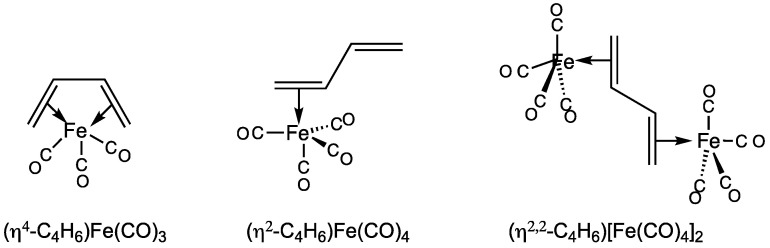
Experimentally known butadiene iron carbonyl complexes.

The observation that butadiene can form stable complexes with metal carbonyls predates the 1951 seminal discovery of ferrocene [1,2] by more than two decades. Thus, in 1930, Reihlen and coworkers [3] found that the reaction of butadiene with Fe(CO)_5_ in an autoclave at elevated temperatures gave essentially air-stable liquid (butadiene)Fe(CO)_3_ that could be purified by vacuum distillation. A 1958 reinvestigation of this species by Hallam and Pauson [4] a few years after the discovery of ferrocene led to the tetrahapto (η^4^-C_4_H_6_)Fe(CO)_3_ structure (Figure 1), in which both C=C double bonds of butadiene are coordinated to the central iron atom, thereby giving it the favored 18-electron configuration [5]. This tetrahapto structure was subsequently confirmed by X-ray crystallography at –40 °C [6]. Later work by Murdoch and Weiss [7] showed that the reaction of butadiene with Fe_2_(CO)_9_ under much milder conditions (40 °C) gave a vacuum distillable liquid dihaptobutadiene derivative (η^2^-C_4_H_6_)Fe(CO)_4_ in which only one of the C=C double bonds of butadiene was coordinated to the iron atom. In addition, Murdoch and Weiss isolated a binuclear (η^2,2^-C_4_H_6_)[Fe(CO)_4_]_2_ derivative in which each C=C double bond of the butadiene ligand was bonded to a separate Fe(CO)_4_ unit.

**Figure 2:**
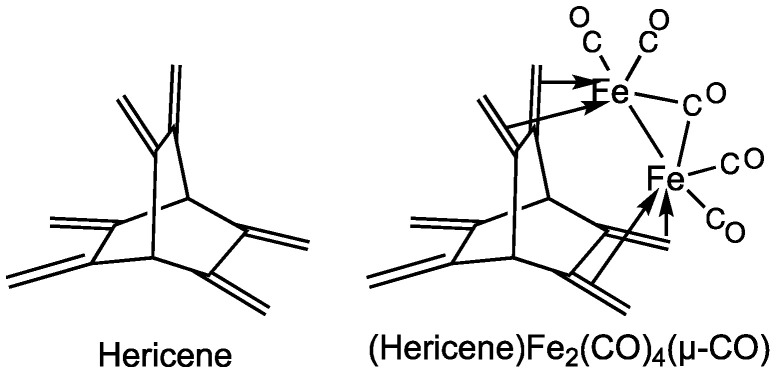
[2.2.2]Hericene and a possible type of binuclear (hericene)Fe_2_(CO)_4_(µ-CO) complex using two of the 1,3-diene units.

**Figure 3:**
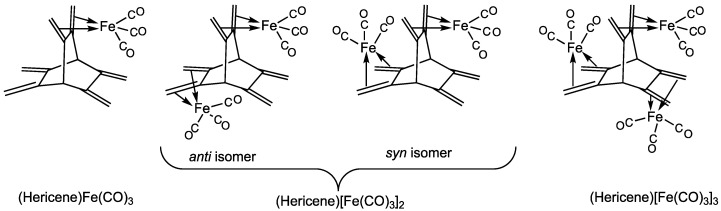
The four types of (hericene)[Fe(CO)_3_]*_n_* (*n* = 1, 2, 3) that have been synthesized.

The observation of the stability of butadiene iron tricarbonyl suggests that hydrocarbons containing multiple 1,3-diene units might serve as ligands in polynuclear iron carbonyl derivatives. The rigidity of the backbone supporting multiple 1,3-diene units might then be used to control the proximity of the metal atoms in such polynuclear metal complexes. In this connection, the hydrocarbon [2.2.2]hericene (formally 2.3.5.6.7.8-hexamethylidenebicyclo [2.2.2]octane) is of interest as containing three equivalent 1,3-diene units supported on a rigid bicyclo [2.2.2]octane framework (Figure 2). The three-fold symmetry of this hericene structure provides three equivalent cavities to accommodate iron tricarbonyl moieties bonded to each of the three 1,3-diene units. Thus, four structures of the type (hericene)[Fe(CO)_3_]*_n_* (*n* = 1, 2, 3) are possible with one, two, or three of these cavities filled by single Fe(CO)_3_ units (Figure 3). For the binuclear complex (hericene)[Fe(CO)_3_]_2_, two stereoisomers are possible. The cavities between the 1,3-diene units are not large enough to accommodate two independent Fe(CO)_3_ units without significant steric repulsion. However, a binuclear Fe_2_(CO)_4_(µ-CO) unit with an iron-iron bond could conceivably be accommodated in a single cavity using the 1,3-diene units on each side of the cavity to bond to the iron atoms in the Fe_2_ system (Figure 2). However, this has not been experimentally realized.

The hydrocarbon [2.2.2]hericene was synthesized in 1980 by Pilet, Birbaum, and Vogel [8] by an eight-step procedure starting from coumalic acid and maleic anhydride. The four possible (hericene)[Fe(CO)_3_]*_n_* (*n* = 1, 2, 3) derivatives with single Fe(CO)_3_ moieties in cavities were synthesized three years later by Hänisch, Tagliaferri, and Roulet [9] by the reaction of hericene with Fe_2_(CO)_9_ and separated by column chromatography. Köhler and Steck [10] (1993) have used hericene as a ligand for the synthesis of the complexes (hericene)[Fe(PMe_3_)_3_]*_n_* (*n* = 1, 2) and (hericene)[Co(η^5^-C_5_H_5_)]*_n_* (*n* = 1, 2, 3).

We now report the first theoretical study on hericene iron carbonyl derivatives in order to direct possible future experimental work with the goal of extending hericene iron carbonyl chemistry beyond that of the experimentally known (hericene)[Fe(CO)_3_]*_n_* derivatives (Figure 3). We are particularly interested in the possibilities for stable molecules containing iron–iron bonds and/or having sterically protected iron atoms with fewer electrons than the favored 18-electron configuration. We have included, in these studies, the (hericene)Fe*_n_*(CO)_3*n*_ derivatives isomeric with the experimentally known species in order to validate our theoretical methods by comparison with experiment. We then extend our studies to include systems of stoichiometry (hericene)Fe_2_(CO)_5_ and (hericene)Fe_3_(CO)_8._ These are the most likely potentially accessible experimental systems by simple decarbonylation of the known (hericene)[Fe(CO)_3_]*_n_* derivatives that have the possibility of being stable molecules with iron–iron bonds and/or coordinatively unsaturated iron atoms.

## 2. Results and Discussion

### 2.1. (hericene)Fe(CO)_3_ Structures

**Figure 4:**
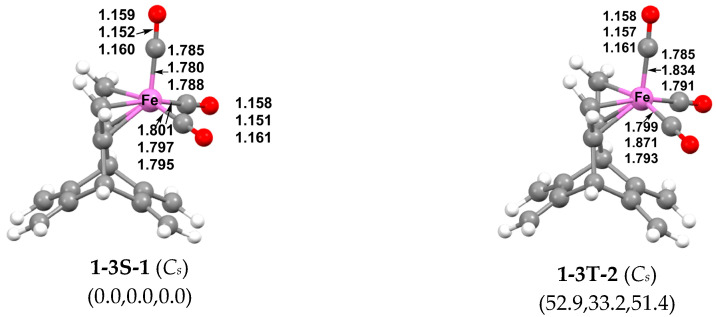
Optimized (hericene)Fe(CO)_3_ structures. In Figure 4, Figure 5, Figure 6, Figure 7 and Figure 8, the numbers in the parentheses are the relative energies in kcal/mol calculated by B3LYP, ωB97XD, and M06-L methods in that order.

For the (hericene)Fe(CO)_3_ compounds, only one low-energy structure, namely, the singlet **1-3S-1** with *C_s_* symmetry, was predicted, corresponding to the structure of the experimentally known (hericene)Fe(CO)_3_ (Figure 4) [9] In **1-3S-1**, the Fe(CO)_3_ unit is fully complexed to one of the three butadiene units, giving the iron atom a favored 18-electron configuration. The corresponding triplet spin state structure **1-3T-2** is of too high an energy to be chemically significant.

### 2.2. Binuclear (hericene)Fe_2_(CO)_n_ (n = 6, 5) Structures

#### 2.2.1. (hericene)Fe_2_(CO)_6_

**Figure 5:**
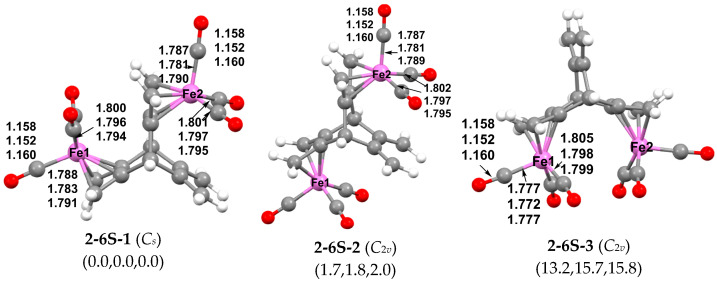
Optimized (hericene)Fe_2_(CO)_6_ structures within 25 kcal/mol of the global minimum.

Three low-energy structures were found for the binuclear (hericene)Fe_2_(CO)_6_ derivatives (Figure 5). The lowest energy structure is the singlet structure **2-6S-1** with *C_s_* symmetry, in which each Fe(CO)_3_ unit is fully complexed to one of the three butadiene units and located in a separate hericene cavity. This gives each iron atom in **2-6S-1** the favored 18-electron configuration. The two Fe(CO)_3_ moieties exhibit *anti* stereochemistry relative to the two 1,3-diene units to which they are bonded. The singlet structure **2-6S-2** lies only 1.7 kcal/mol (B3LYP), 1.8 kcal/mol (ωB97XD), or 2.0 kcal/mol (M06-L) higher in energy than the lowest energy structure **2-6S-1**. These results are within the computational error, so structures **2-6S-1** and **2-6S-2** can be considered to be essentially isoenergetic. Structure **2-6S-2** is similar to **2-6S-1** except for the *syn* stereochemistry of its Fe(CO)_3_ moiety relative to the two 1,3-diene units. The two essentially isoenergetic (hericene)Fe_2_(CO)_6_ structures **2-6S-1** and **2-6S-2** are both formed in the reaction of hericene with Fe_2_(CO)_9_ and separated by column chromatography [9]. The third (hericene)Fe_2_(CO)_6_ structure **2-6S-3**, like both **2-6S-1** and **2-6S-2**, consists of two (1,3-diene)Fe(CO)_3_ units, but they are located in the same cavity of the hericene ligand. Because of the resulting steric interaction, structure **2-6S-3** is of significantly higher energy than **2-6S-1** and **2-6S-2**, namely, 13.2 kcal/mol (B3LYP), 15.7 kcal/mol (ωB97XD) or 15.8 kcal/mol (M06-L) above **2-6S-1**. In **2-6S-3** without an iron–iron bond, both iron atoms have the favored 18-electron configuration as in both **2-6S-1** and **2-6S-2.** Thus, the Fe⋯Fe distance of 4.368 Å (B3LYP), 4.237 Å (ωB97XD), or 4.234 Å (M06-L) in **2-6S-3** is clearly a non-bonding distance.

#### 2.2.2. (hericene)Fe_2_(CO)_5_

**Figure 6:**
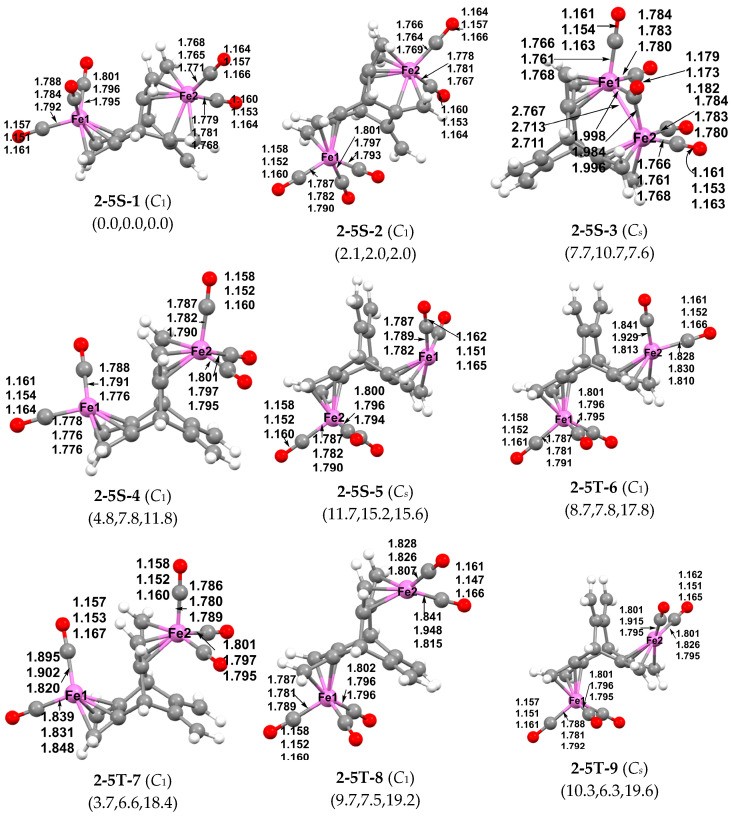
Optimized (hericene)Fe_2_(CO)_5_ structures within 15 kcal/mol of the global minimum.

Removing one carbonyl group from (hericene)Fe_2_(CO)_6_ to give (hericene)Fe_2_(CO)_5_ leads to nine structures within 15 kcal/mol of the lowest energy structure. The lowest energy (hericene)Fe_2_(CO)_5_ structure is the singlet structure **2-5S-1** with an Fe(CO)_3_ group complexed to a 1,3-diene unit in one of the ligand cavities and an Fe(CO)_2_ group complexed to three of the four double bonds in two 1,3-diene units flanking a second ligand cavity (Figure 6). The two Fe(CO)*_n_* moieties are on opposite sides of the hericene ligand in an *anti* configuration. The resulting coordination of a total of five of the six C=C double bonds in the hericene ligand with the two iron carbonyl moieties gives each iron atom the classical 18-electron configuration. The second (hericene)Fe_2_(CO)_5_ structure **2-5S-2**, lying only ~2.0 kcal/mol in energy above **2-5-S1**, has the same coordination mode as that in structure **2-5S-1**, but with the *syn* configuration of the two Fe(CO)*_n_* moieties on the same side of the hericene ligand.

The third (hericene)Fe_2_(CO)_5_ isomer **2-5S-3** is a *C_s_* symmetry structure lying 7.7 kcal/mol (B3LYP), 10.7 kcal/mol (ωB97XD), or 7.6 kcal/mol (M06-L) in energy above **2-5S-1**. In **2-5S-3**, a Fe_2_(CO)_4_(µ-CO) unit is located in a single hericene cavity with each iron atom fully bonded to a 1,3-diene unit flanking the cavity. The two iron atoms in **2-5S-3** form an Fe–Fe single bond of length ~2.7 Å bridged by one of the five CO groups. This bridging µ-CO group exhibits a ν(CO) frequency of 1902 cm^−1^ (M06-L) that is significantly lower than the terminal ν(CO) frequencies ranging from 2022 to 2095 cm^−1^. This bonding arrangement gives each iron atom in **2-5S-3** the favored 18-electron configuration. The higher energy of **2-5S-3** relative to **2-5S-1** and **2-5S-2** can be related to the strain associated with fitting two iron atoms into a single hericene cavity.

The remaining six (hericene)Fe_2_(CO)_5_ structures within 15 kcal/mol of energy of **2-5S-1** are various stereoisomers having an Fe(CO)_3_ moiety bonded fully to the 1,3-diene unit in one hericene cavity and an Fe(CO)_2_ moiety bonded fully to the 1,3-diene unit in another hericene cavity (Figure 6). Thus, the iron in the Fe(CO)_3_ moiety has the favored 18-electron configuration whereas the iron in the Fe(CO)_2_ moiety has only a 16-electron configuration. Structures **2-5S-4** and **2-5S-5** are low-spin singlet structures whereas structures **2-5T-6**, **2-5T-7**, **2-5T-8**, and **2-5T-9** are high-spin triplet structures. There do not appear to be major differences in the relative energies of singlet and spin state (hericene)[Fe(CO)_3_][Fe(CO)_2_] isomers of this type. Additional related triplet structures with different stereochemistry lie at still higher energies significantly more than 15 kcal/mol above **2-5S-1**.

### 2.3. Trinuclear (hericene)Fe_3_(CO)_n_ (n = 9, 8) Structures

#### 2.3.1. (hericene)Fe_3_(CO)_9_

**Figure 7:**
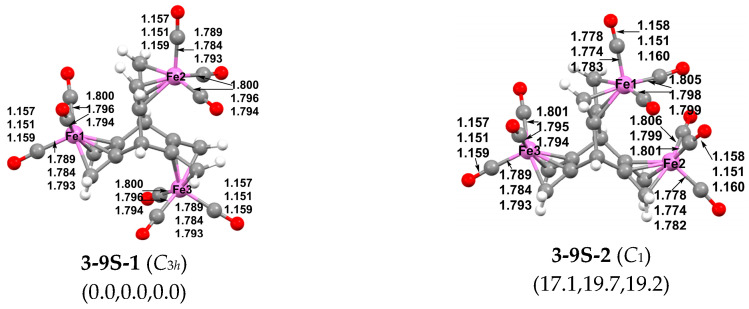
Two optimized energetically low-lying (hericene)Fe_3_(CO)_9_ structures.

The lowest energy structure for the trinuclear (hericene)Fe_3_(CO)_9_ is singlet **3-9S-1** with three-fold C*_3h_* symmetry, which is one of the products experimentally isolated from the reaction of Fe_2_(CO)_9_ with hericene (Figure 7) [9] In **3-9S-1**, the three Fe(CO)_3_ moieties are coordinated to the three hericene 1,3-diene units in a windmill type of structure having one Fe(CO)_3_ moiety in each of the three hericene cavities. Each iron atom in **3-9S-1** acquires the favored 18-electron configuration, consistent with the singlet spin state. The considerably higher energy singlet (hericene)Fe_3_(CO)_9_ isomer **3-9S-2**, lying 17.1 kcal/mol (B3LYP), 19.7 kcal/mol (ωB97XD), or 19.2 kcal/mol (M06-L) above **3-9S-1**, also has an Fe(CO)_3_ moiety bonded to each of the three hericene 1,3-diene units. However, in **3-9S-2**, two of the Fe(CO)_3_ moieties are squeezed into the same hericene cavity. The steric interference between the two Fe(CO)_3_ moieties crammed into the same hericene cavity accounts for the significantly higher energy of **3-9S-2** relative to **3-9S-1**. In **3-9S-2** the long Fe…Fe distance of 4.368 Å (B3LYP), 4.237 Å (ωB97XD), or 4.234 Å (M06-L) between the iron atoms of the two Fe(CO)_3_ moieties in the same hericene cavity suggests no direct iron–iron interaction. This is consistent with the favored 18-electron configurations of each of the iron atoms in **3-9S-2**.

#### 2.3.2. (hericene)Fe_3_(CO)_8_

**Figure 8:**
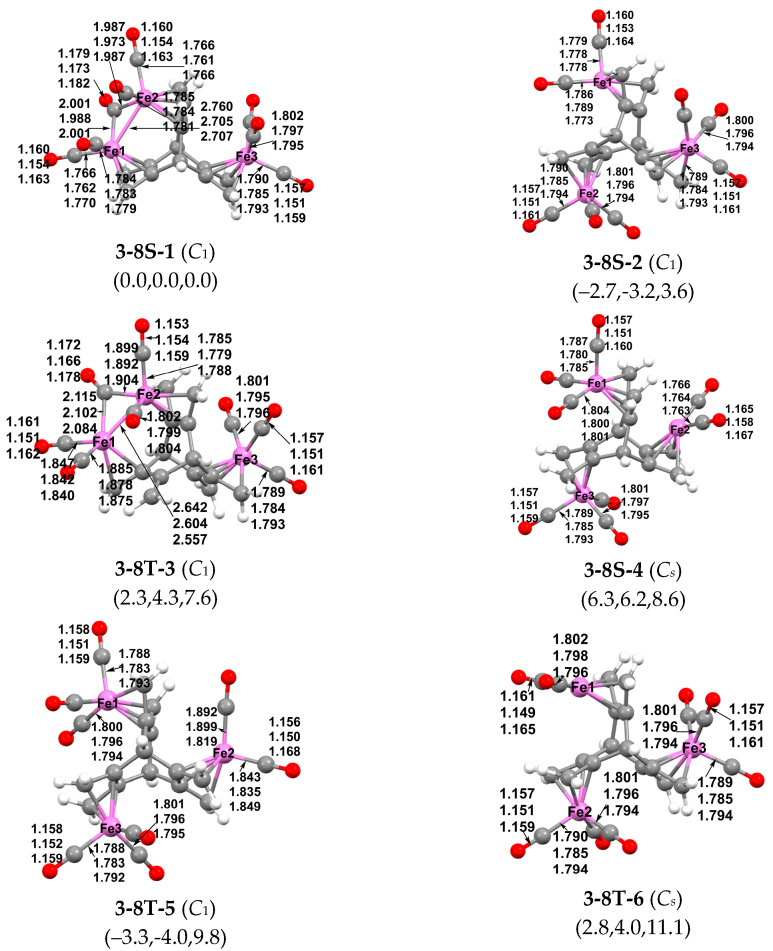
The six lowest-energy optimized (hericene)Fe_3_(CO)_8_ structures.

The lowest energy (hericene)Fe_3_(CO)_8_ structure predicted by the M06-L method is the singlet structure **3-8S-1**. In **3-8S-1**, an Fe_2_(CO)_5_(µ-CO) moiety is bonded to both 1,3-diene units in the same hericene cavity and an Fe(CO)_3_ moiety is bonded to the remaining hericene 1,3-diene unit in a second hericene cavity (Figure 8). The two iron atoms of the Fe_2_(CO)_4_(µ-CO) moiety form a single bond of length 2.760 Å (B3LYP), 2.705 Å (ωB97XD), or 2.707 Å (M06-L) bridged by one of the CO groups. This bridging CO group exhibits a ν(CO) frequency of 1900 cm^−1^ (M06-L) that is significantly lower than the terminal ν(CO) frequencies ranging from 2025 to 2113 cm^−1^. This bonding arrangement leads to the favored 18-electron configuration for each of the three iron atoms in **3-8S-1**. The triplet (hericene)Fe_3_(CO)_8_ structure **3-8T-3**, lying 2.4 kcal/mol (B3LYP), 4.3 kcal/mol (ωB97XD), or 7.6 kcal/mol (M06-L) in energy above **3-8S-1**, is closely related to the singlet structure **3-8S-1**. However, in **3-8T-3**, only one of the C=C double bonds of a hericene 1,3-diene unit, rather than both C=C bonds, is bonded to one of the iron atoms of the Fe_2_(CO)_4_(µ-CO) moiety. This leads an uncomplexed C=C double bond of length 1.344 Å (B3LYP), 1.338 Å (ωB97XD), or 1.343 Å (M06-L). The iron atom in **3-8T-3** bonded to only one C=C double bond has only a 16-electron configuration, consistent with the triplet state in a high-spin configuration.

The singlet (hericene)Fe_3_(CO)_8_ structure **3-8S-2** has essentially the same energy as **3-8S-1**, lying 3.6 kcal/mol (M06-L) above **3-8S-1** or 2.7 kcal/mol (B3LYP) or 3.2 kcal/mol (ωB97XD) below **3-8S-1** (Figure 8). Structure **3-8S-2** has an Fe(CO)*_n_* (*n* = 2 or 3) moiety in each of the three hericene cavities fully bonded to a 1,3-diene unit. The iron atoms in the two Fe(CO)_3_ moieties have the favored 18-electron configuration whereas the iron atom in the Fe(CO)_2_ moiety has only a 16-electron configuration. Thus, structure **3-8S-2** can be derived from the (hericene)Fe_3_(CO)_9_ structure **3-9S-1** (Figure 7) by removal of one of the carbonyl groups. Structure **3-8S-4**, lying 6.3 kcal/mol (B3LYP), 6.2 kcal/mol (ωB97XD), or 8.6 kcal/mol (M06-L) in energy above **3-8S-1**, is similar to **3-8S-2** except for the angle between the two terminal CO groups in the Fe(CO)_2_ moiety.

The singlet (hericene)Fe_3_(CO)_8_ structures **3-8S-2** and **3-8S-4** can be considered as low-spin versions of trinuclear structures, with two iron atoms having the favored 18-electron configuration, but the third iron atom having only a 16-electron configuration. The high-spin triplet versions of **3-8S-2** and **3-8S-4** are **3-8T-5** and **3-8T-6**, respectively (Figure 8). The apparent energy differences between the singlet and triplet electronic states of these structures depend on the DFT method and suggest that there are no major energy differences between the singlet and triplet versions of these structures.

### 2.4. Thermochemistry

Table 1 reports the energies of the single carbonyl dissociation reactions for the lowest-lying structures predicted by the M06-L method for the (hericene)Fe*_m_*(CO)*_n_* (*m* = 3, 2; *n* = 9, 6) compounds. These CO dissociation energies are found to be substantial, namely, 44.5 kcal/mol for (hericene)Fe_3_(CO)_9_ and 44.2 kcal/mol for (hericene)Fe_2_(CO)_6_, indicating the viabilities of such compounds toward CO loss. In addition, Table 1 reports the fragmentation energies of the binuclear and trinuclear (hericene)Fe*_m_*(CO)*_n_* derivatives by loss of Fe(CO)_3_ and Fe_2_(CO)_6_ moieties by the following reactions:

(hericene)Fe_3_(CO)_9_ → (hericene)Fe_2_(CO)_6_ + Fe(CO)_3_

(hericene)Fe_3_(CO)_9_ → (hericene)Fe(CO)_3_ + Fe_2_(CO)_6_

(hericene)Fe_2_(CO)_6_ → (hericene)Fe(CO)_3_ + Fe(CO)_3_

Such dissociation energies are predicted to be very high (57.5 to 94.5 kcal/mol) by all three DFT methods, suggesting difficulty in breaking down the binuclear and trinuclear structures.

## 3. Theoretical Methods

Density functional theory (DFT) is well established to be a useful and reliable method to predict the structures and energetics of many transition metal complexes [11,12,13]. In this study, computations were performed on the Gaussian 09 suite of programs [14] using three different DFT methods. The first method was B3LYP based on Becke’s three-parameter functional (B3) [15] combined with the Lee–Yang–Parr generalized gradient correlation functional (LYP) [16]. The second was the M06-L method that has been reported to perform very well for predicting geometries and vibrational frequencies [17]. The third method used the ωB97xD functional, which is the latest functional from Head-Gordon and coworkers and includes empirical dispersion [18]. The ωB97XD functional has been shown to perform well for transition metal compounds [19]. The M06-L method was used to predict ν(CO) frequencies without any scaling factors.

Double-ζ plus polarization (DZP) basis sets were used for these calculations. For carbon and oxygen, one set of pure spherical harmonic d functions with orbital exponents α_d_(C) = 0.75 and α_d_(O) = 0.85 was added to the standard Huzinaga–Dunning contracted DZ sets. This basis set is designated (9s5p1d/4s2p1d) [20,21]. For hydrogen, a set of p polarization functions α_p_(H) = 0.75 was added to the Huzinaga–Dunning DZ sets. For iron, in our loosely contracted DZP (14s11p6d/10s8p3d) basis sets, the Wachters’ primitive sets were used, but augmented by two sets of p functions and one set of d functions, contracted following Hood et al. [22,23].

The optimized (hericene)Fe_m_(CO)_n_ structures in this paper are designated as **m-nX-z**, where **m** represents the number of iron atoms, **n** is the number of carbonyl groups, **z** is the relative energy structure sequence number predicted according to the M06-L method, and **X** refers to the spin state with **S** and **T**, representing the singlet and triplet spin states, respectively.

## 4. Summary

The four species obtained from the reaction of Fe_2_(CO)_9_ with hericene, namely, the (hericene)Fe(CO)_3_ structure **1-3S-1**, the two isomeric (hericene)[Fe(CO)_3_]_2_ structures **2-6S-1** and **2-6S-2**, and the (hericene)[Fe(CO)_3_]_3_ structure **3-9S-1**, are the lowest-energy structures of those stoichiometries. These four structures gratifyingly correspond to the four experimentally known hericene iron carbonyl derivatives. Such structures all have exclusively (η^4^-1,3-diene)Fe(CO)_3_ subunits with only a single Fe(CO)_3_ moiety in any of the three cavities of the hericene structure. This leads to a windmill-like structure for the lowest-energy trinuclear complex (hericene)[Fe(CO)_3_]_3_. Isomeric binuclear and trinuclear (hericene)[Fe(CO)_3_]*_x_* (*x* = 2, 3) structures with two Fe(CO)_3_ moieties in a single hericene cavity are significantly higher-energy structures.

Loss of a CO group from the binuclear and trinuclear (hericene)[Fe(CO)_3_]*_x_* structures leads to significantly more complicated potential energy surfaces. For the (hericene)Fe_2_(CO)_5_ system, the lowest energy geometries are singlet structures with an Fe(CO)_3_ unit bonded to a 1,3-diene unit in one hericene cavity. This structure also features an Fe(CO)_2_ unit bonded to two C=C double bonds in one 1,3-diene unit and one C=C double bond in another 1,3-diene unit. Higher-energy (hericene)Fe_2_(CO)_5_ structures include a structure in which a single hericene cavity contains a Fe_2_(CO)_4_(µ-CO) moiety with a single bridging CO group in which each iron atom is bonded to a 1,3-diene unit. In addition, both singlet and triplet structures are found in which an Fe(CO)_3_ moiety and an Fe(CO)_2_ moiety located in separate hericene cavities are each bonded to a 1,3-diene unit. The lowest-energy (hericene)Fe_3_(CO)_8_ structures have two hericene cavities containing Fe(CO)_3_ moieties fully bonded to 1,3-diene units. In these structures, the third hericene cavity contains an Fe(CO)_2_ moiety fully bonded to a 1,3-diene unit, leading to only a 16-electron configuration for the latter iron atom. Such structures can be derived from the experimentally known [9] windmill-like (hericene)[Fe(CO)_3_]_3_ structures by the loss of a CO group.

## Figures and Tables

**Table 1:** Dissociation energies (kcal/mol) of CO, Fe(CO)_3_, and Fe_2_(CO)_6_ from the (hericene)Fe_3_(CO)_9_,(hericene)Fe_2_(CO)_6_, and (hericene)Fe(CO)_3_ derivatives.

	B3LYP	ωB97XD	M06-L
(hericene)Fe_3_(CO)_9_ → (hericene)Fe_3_(CO)_8_ + CO	43.7	47.5	44.5
(hericene)Fe_2_(CO)_6_ → (hericene)Fe_2_(CO)_5_ + CO	36.8	36.8	44.2
(hericene)Fe_3_(CO)_9_ → (hericene)Fe_2_(CO)_6_ + Fe(CO)_3_	57.5	76.5	77.2
(hericene)Fe_2_(CO)_6_ → (hericene)Fe(CO)_3_ + Fe(CO)_3_	59.9	77.9	78.8
(hericene)Fe_3_(CO)_9_ → hericene)Fe(CO)_3_ + Fe_2_(CO)_6_	61.2	94.5	89.2

## Data Availability

Data are contained within the article and Supplementary Materials.

## Supplementary Materials

The following supporting information can be downloaded at: https://www.mdpi.com/article/10.3390/molecules29225337/s1, Figure S1: Optimized (hericene)Fe*_m_*(CO)*_n_* (*m* = 1, *n* = 3; *m* = 2, *n* = 6, 5; *m* = 3, *m* = 9, 8) structures; Tables S1–S27: Coordinates of the optimized (hericene)Fe*_m_*(CO)*_n_* structures (*m* = 1, *n* = 3; *m* = 2, *n* = 6, 5; *m* = 3, *m* = 9, 8) calculated using the B3LYP, *w*B97XD, and M06-L methods; Tables S28–S54: Harmonic vibrational frequencies (in cm^−1^) and infrared intensities (in parentheses in km/mol) of the optimized (hericene)Fe*_m_*(CO)*_n_* structures (*m* = 1, *n* = 3; *m* = 2, *n* = 6, 5; *m* = 3, *m* = 9, 8) calculated using the B3LYP, *w*B97XD, and M06-L methods; Table S55: Harmonic vibrational frequencies (in cm^−1^) ν(CO) and infrared intensities (in parentheses, in km/mol) for the (hericene)Fe_3_(CO)*_n_*, (hericene)Fe_2_(CO)*_n_*, and (hericene)Fe(CO)_n_ derivatives; Table S56: Natural charges, Wiberg bond indices, Fe–Fe distances, and formal Fe–Fe bond orders for the optimized (hericene)Fe*_m_*(CO)*_n_* structures (*m* = 1, *n* = 3; *m* = 2, *n* = 6, 5; *m* = 3, *m* = 9, 8) using the M06-L method; Complete Gaussian09 reference (Reference [24]).
