# Supplementary material for: Iron Carbonyl Complexes of [2.2.2]Hericene as a Rigid Tris(1,3-diene) Ligand
Source: Molecules. 2024 Nov 13;29(22):5337. doi: 10.3390/molecules29225337 (PMC11596961; doi:10.3390/molecules29225337)
Supplement: Supplementary file 1 [file molecules-29-05337-s001.zip › molecules-3282873-supplementary.pdf]

Iron Carbonyl Complexes of [2.2.2]Hericene as a Rigid Tris(1,3-diene) Ligand

Jinfeng Luo,[a] Haoyu Chen [a] Huidong Li, [a,b]*Yongxiang Zheng, [a] Qunchao Fan, [a] R. Bruce King,[b]* and Henry F. Schaefer, III[b]

[a] *School of Science, Key Laboratory of High Performance Scientific Computation, Xihua University, Chengdu, China 610039*
[b]*Center for Computational Quantum Chemistry, University of Georgia, Athens, Georgia, USA 30602*

rbking@chem.uga.edu; huidongli@mail.xhu.edu.cn

Supporting Information

Figures S1: Optimized structures of the (**Herience)$\mathbf{Fe_m(CO)_n}$** compounds (m = 3,2,1; n = 9,8,6,5,3).

Tables S1 to S27: Optimized coordinates of the optimized structures of the (**Herience)$\mathbf{Fe_m(CO)_n}$** calculated with the B3LYP method, *w*B97XD method and M06-L method.

Tables S28 to S54: Harmonic vibrational frequencies (in cm^{-1}) and infrared intensities (in parentheses in km/mol) of the optimized structures of the (**Herience)$\mathbf{Fe_m(CO)_n}$** calucated with the B3LYP method,*w*B97XD method and M06-L method.

Tables S55: Harmonic vibrational frequencies (in cm-1) and infrared intensities (in parentheses, in km/mol) for the (Hericene)$Fe_3(CO)_n$, (Hericene)$Fe_2(CO)_n$, and (Hericene)$Fe(CO)_n$ derivatives.

Tables S56: Natural charges, Wiberg bond indices, Fe-Fe distances, and formal Fe-Fe bond orders for the (Hericene)$Fe_m(CO)_n$ Complexes using the M06-L method.

Complete Gaussian09 reference (Reference 23)

**(Hericene)Fe(CO)$_3$**	
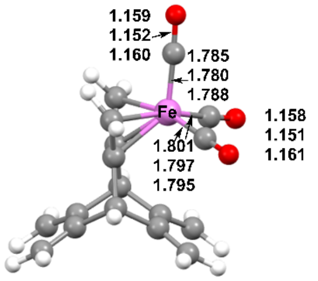<br>1-3S-1(C_s)<br>(0.0,0.0,0.0)	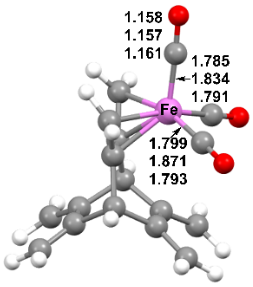<br>1-3T-2(Cs)<br>(52.9,33.2,51.4)

**(Hericene)Fe$_2$(CO)$_n$ (n=6,5)**		
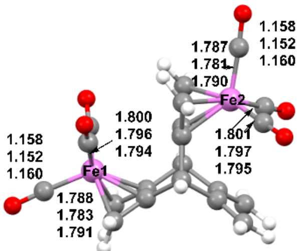<br>2-6S-1(C_s)<br>(0.0,0.0,0.0)	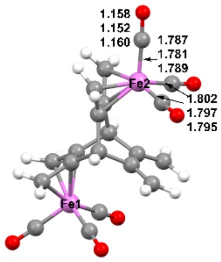<br>2-6S-2(C_{2v})<br>(1.7,1.8,2.0)	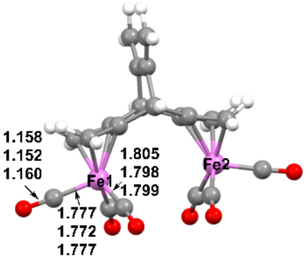<br>2-6S-3(C_{2v})<br>(13.2,15.7,15.8)
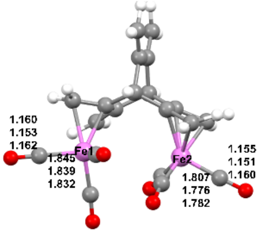<br>2-6T-4(C_1)<br>(28.9,33.5,34.1)	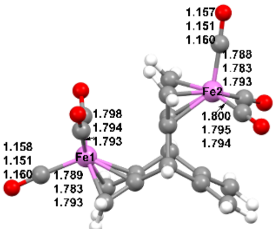<br>2-6T-5(C_s)<br>(55.3,57.7,54.5)	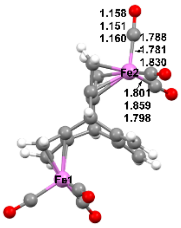<br>2-6T-6(C_{2v})<br>(56.2,27.1,62.5)
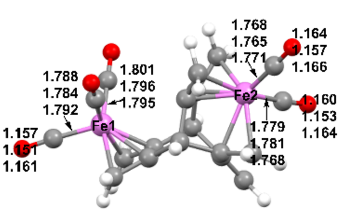<br>2-5S-1(C_1)<br>(0.0,0.0,0.0)	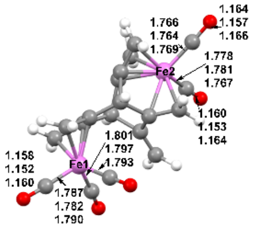<br>2-5S-2(C_1)<br>(2.1,2.0,2.0)	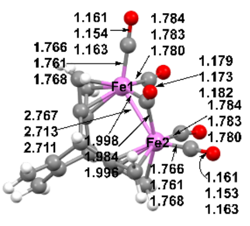<br>2-5S-3(C_s)<br>(7.7,10.7,7.6)

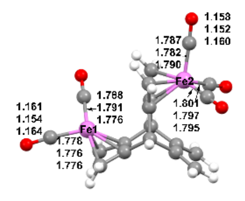 2-5S-4(C_1) (4.8,7.8,11.8)	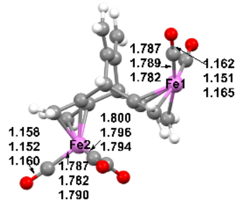 2-5S-5(C_s) (11.7,15.2,15.6)	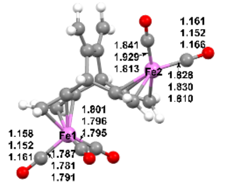 2-5T-6(C_1) (8.7,7.8,17.8)
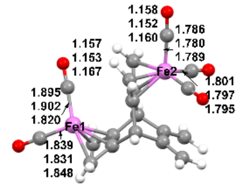 2-5T-7(C_1) (3.7,6.6,18.4)	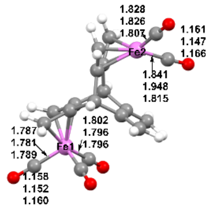 2-5T-8(C_1) (9.7,7.5,19.2)	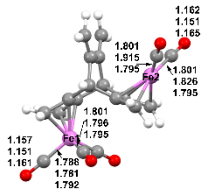 2-5T-9(Cs) (10.3,6.3,19.6)
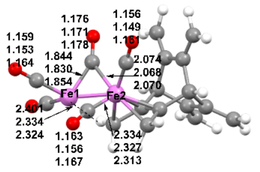 2-5S-10(C_1) (19.8,30.0,26.1)	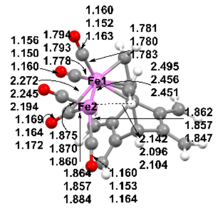 2-5T-11(C_1) (18.3,25.3,27.0)	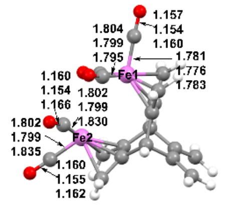 2-5T-12(C_1) (15.5,20.5,29.9)

(Hericene)$Fe_3(CO)_n$ (n=9,8)

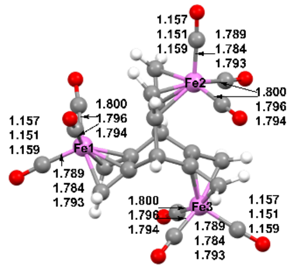 3-9S-1(C_{3h}) (0.0,0.0,0.0)	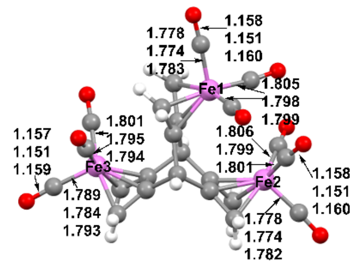 3-9S-2(C_1) (17.1,19.7,19.2)
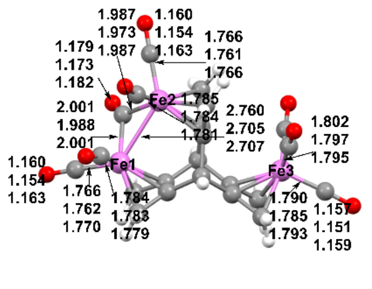 3-8S-1(C_1) (0.0,0.0,0.0)	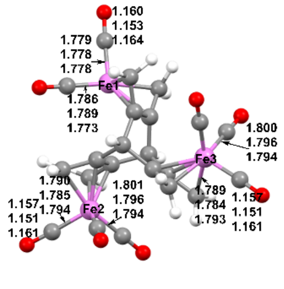 3-8S-2(C_1) (-2.7,-3.2,3.6)

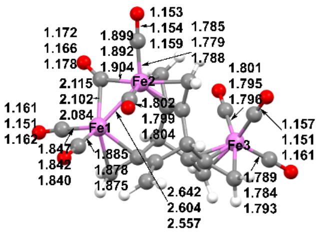

3-8T-3(C_1)

(2.3,4.3,7.6)

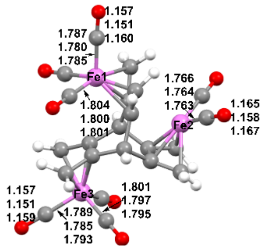

3-8S-4(C_s)

(6.3,6.2,8.6)

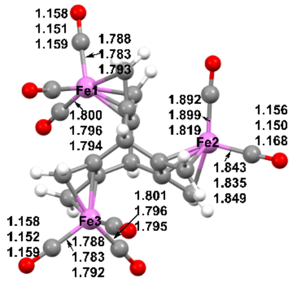

3-8T-5(C_1)

(-3.3,-4.0,9.8)

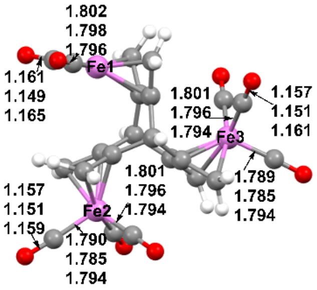

3-8T-6(C_s)

(2.8,4.0,11.1)

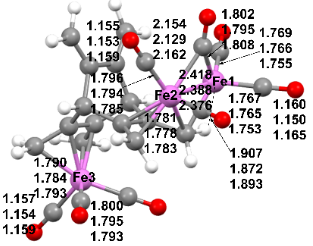

3-8S-7(C_1)

(11.0,16.7,15.8)

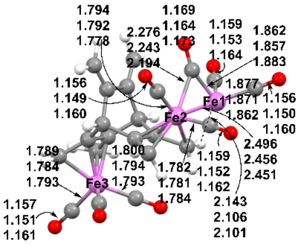

3-8T-8(C_1)

(9.1,12.9,17.4)

Figure S1. Optimized structures of the (**Hericene**)**Fe**$_m$**(CO)**$_n$ compounds (m = 3,2,1; n = 9,8,6,5,3).

Table S1:Optimized coordinates for the **(Hericene)Fe$_m$(CO)$_n$** structure **1-3S-1**.

	B3LYP				M06L				wB97XD		
	x	y	z		x	y	z		x	y	z
C	-1.339038	1.483152	0.743840	C	-1.312855	1.406440	0.737930	C	-1.321106	1.437795	0.743623
C	0.083560	1.479373	1.298672	C	0.098639	1.446537	1.294382	C	0.095971	1.452068	1.296709
C	0.083560	1.479373	-1.298672	C	0.098639	1.446537	-1.294382	C	0.095971	1.452068	-1.296709
C	-1.339038	1.483152	-0.743840	C	-1.312855	1.406440	-0.737930	C	-1.321106	1.437795	-0.743623
C	0.880527	0.317947	0.708885	C	0.915805	0.310314	0.708293	C	0.895926	0.300767	0.706390
C	0.880527	0.317947	-0.708885	C	0.915805	0.310314	-0.708293	C	0.895926	0.300767	-0.706390
C	0.775549	2.734597	-0.745769	C	0.749998	2.710117	-0.739984	C	0.768381	2.710058	-0.745701
C	0.775549	2.734597	0.745769	C	0.749998	2.710117	0.739984	C	0.768381	2.710058	0.745701
C	1.594384	-0.715563	1.388588	C	1.623385	-0.737727	1.366798	C	1.593011	-0.748677	1.376707
C	1.594384	-0.715563	-1.388588	C	1.623385	-0.737727	-1.366798	C	1.593011	-0.748677	-1.376707
C	-2.418569	1.518440	1.542674	C	-2.394507	1.400010	1.530023	C	-2.398809	1.448199	1.535047
C	-2.418569	1.518440	-1.542674	C	-2.394507	1.400010	-1.530023	C	-2.398809	1.448199	-1.535047
C	1.295639	3.681355	-1.544223	C	1.243907	3.673140	-1.530961	C	1.273220	3.662189	-1.537004
C	1.295639	3.681355	1.544223	C	1.243907	3.673140	1.530961	C	1.273220	3.662189	1.537004
H	-2.318338	1.526282	2.625701	H	-2.299889	1.402224	2.612248	H	-2.298695	1.451785	2.617036
H	1.243837	3.594808	2.627325	H	1.197331	3.590884	2.613094	H	1.221950	3.574584	2.618994
H	1.243837	3.594808	-2.627325	H	1.197331	3.590884	-2.613094	H	1.221950	3.574584	-2.618994
H	1.435355	-0.820435	2.461165	H	1.475328	-0.856473	2.438625	H	1.430823	-0.848407	2.448543
H	1.435355	-0.820435	-2.461165	H	1.475328	-0.856473	-2.438625	H	1.430823	-0.848407	-2.448543
H	0.088356	1.470190	2.392570	H	0.100283	1.438323	2.388225	H	0.101654	1.439051	2.389191
H	0.088356	1.470190	-2.392570	H	0.100283	1.438323	-2.388225	H	0.101654	1.439051	-2.389191
Fe	0.236574	-1.560933	0.000000	Fe	0.244018	-1.525405	0.000000	Fe	0.237698	-1.533697	0.000000
C	-0.977515	-1.853438	1.297579	C	-0.974162	-1.759990	1.297129	C	-0.978302	-1.787002	1.298014
C	-0.977515	-1.853438	-1.297579	C	-0.974162	-1.759990	-1.297129	C	-0.978302	-1.787002	-1.298014
C	1.092717	-3.127720	0.000000	C	1.046431	-3.123355	0.000000	C	1.059620	-3.112509	0.000000
O	-1.731449	-2.074854	-2.147561	O	-1.734210	-1.934667	-2.157528	O	-1.726410	-1.970972	-2.153262
O	1.682028	-4.125272	0.000000	O	1.615339	-4.135345	0.000000	O	1.643398	-4.106067	0.000000
O	-1.731449	-2.074854	2.147561	O	-1.734210	-1.934667	2.157528	O	-1.726410	-1.970972	2.153262
H	1.782968	4.568690	-1.145197	H	1.703982	4.570110	-1.123590	H	1.747653	4.553716	-1.134818
H	1.782968	4.568690	1.145197	H	1.703982	4.570110	1.123590	H	1.747653	4.553716	1.134818
H	2.601376	-0.974455	-1.064480	H	2.625004	-0.995957	-1.024281	H	2.604052	-1.001153	-1.061932
H	2.601376	-0.974455	1.064480	H	2.625004	-0.995957	1.024281	H	2.604052	-1.001153	1.061932
H	-2.318338	1.526282	-2.625701	H	-2.299889	1.402224	-2.612248	H	-2.298695	1.451785	-2.617036
H	-3.430906	1.534966	1.144515	H	-3.402494	1.376875	1.123707	H	-3.408438	1.445682	1.132806
H	-3.430906	1.534966	-1.144515	H	-3.402494	1.376875	-1.123707	H	-3.408438	1.445682	-1.132806

Table S2:Optimized coordinates for the **(Hericene)Fe$_m$(CO)$_n$** structure **1-3T-2**.

	B3LYP				M06L				wB97XD		
	x	y	z		x	y	z		x	y	z
C	-1.339038	1.483152	0.743840	C	-1.250365	1.434903	0.689416	C	-1.331688	1.253020	0.743908
C	0.083560	1.479373	1.298672	C	0.138789	1.409104	1.287096	C	0.055115	1.550110	1.294548
C	0.083560	1.479373	-1.298672	C	0.138789	1.409104	-1.287096	C	0.055115	1.550110	-1.294548
C	-1.339038	1.483152	-0.743840	C	-1.250365	1.434903	-0.689416	C	-1.331688	1.253020	-0.743908
C	0.880527	0.317947	0.708885	C	0.944670	0.259944	0.706835	C	1.054863	0.542136	0.739605
C	0.880527	0.317947	-0.708885	C	0.944670	0.259944	-0.706835	C	1.054863	0.542136	-0.739605
C	0.775549	2.734597	-0.745769	C	0.814995	2.666065	-0.738020	C	0.478496	2.908461	-0.744837
C	0.775549	2.734597	0.745769	C	0.814995	2.666065	0.738020	C	0.478496	2.908461	0.744837
C	1.594384	-0.715563	1.388588	C	1.628589	-0.802583	1.367406	C	1.861736	-0.213993	1.519381
C	1.594384	-0.715563	-1.388588	C	1.628589	-0.802583	-1.367406	C	1.861736	-0.213993	-1.519381
C	-2.418569	1.518440	1.542674	C	-2.421424	1.513140	1.515659	C	-2.387539	1.038022	1.536353
C	-2.418569	1.518440	-1.542674	C	-2.421424	1.513140	-1.515659	C	-2.387539	1.038022	-1.536353
C	1.295639	3.681355	-1.544223	C	1.310409	3.626446	-1.534421	C	0.794903	3.937761	-1.537786
C	1.295639	3.681355	1.544223	C	1.310409	3.626446	1.534421	C	0.794903	3.937761	1.537786
H	-2.318338	1.526282	2.625701	H	-2.346175	1.449373	2.596991	H	-2.287785	1.054614	2.618184
H	1.243837	3.594808	2.627325	H	1.270003	3.538099	2.616203	H	0.763950	3.840024	2.619712
H	1.243837	3.594808	-2.627325	H	1.270003	3.538099	-2.616203	H	0.763950	3.840024	-2.619712
H	1.435355	-0.820435	2.461165	H	1.475739	-0.920363	2.438658	H	1.758480	-0.191236	2.600389
H	1.435355	-0.820435	-2.461165	H	1.475739	-0.920363	-2.438658	H	1.758480	-0.191236	-2.600389
H	0.088356	1.470190	2.392570	H	0.125083	1.399683	2.380789	H	0.057703	1.545518	2.387165
H	0.088356	1.470190	-2.392570	H	0.125083	1.399683	-2.380789	H	0.057703	1.545518	-2.387165
Fe	0.236574	-1.560933	0.000000	Fe	0.227960	-1.553714	0.000000	Fe	0.169893	-1.555409	0.000000
C	-0.977515	-1.853438	1.297579	C	-1.005501	-1.705003	1.292548	C	-0.895436	-2.078184	1.447466
C	-0.977515	-1.853438	-1.297579	C	-1.005501	-1.705003	-1.292548	C	-0.895436	-2.078184	-1.447466
C	1.092717	-3.127720	0.000000	C	0.964579	-3.185858	0.000000	C	1.284576	-3.012762	0.000000
O	-1.731449	-2.074854	-2.147561	O	-1.785124	-1.786491	-2.150220	O	-1.533989	-2.457243	-2.331125
O	1.682028	-4.125272	0.000000	O	1.489545	-4.221252	0.000000	O	1.935773	-3.970273	0.000000
O	-1.731449	-2.074854	2.147561	O	-1.785124	-1.786491	2.150220	O	-1.533989	-2.457243	2.331125
H	1.782968	4.568690	-1.145197	H	1.766340	4.525930	-1.127983	H	1.090500	4.903924	-1.137097
H	1.782968	4.568690	1.145197	H	1.766340	4.525930	1.127983	H	1.090500	4.903924	1.137097
H	2.601376	-0.974455	-1.064480	H	2.625065	-1.082267	-1.025991	H	2.676398	-0.807447	-1.117647
H	2.601376	-0.974455	1.064480	H	2.625065	-1.082267	1.025991	H	2.676398	-0.807447	1.117647
H	-2.318338	1.526282	-2.625701	H	-2.346175	1.449373	-2.596991	H	-2.287785	1.054614	-2.618184
H	-3.430906	1.534966	1.144515	H	-3.411437	1.610242	1.080663	H	-3.375461	0.828241	1.135336
H	-3.430906	1.534966	-1.144515	H	-3.411437	1.610242	-1.080663	H	-3.375461	0.828241	-1.135336

Table S3:Optimized coordinates for the **(Hericene)Fe$_m$(CO)$_n$** structure **2-5S-1.**

	B3LYP				M06L				wB97XD		
	x	y	z		x	y	z		x	y	z
C	-0.587926	0.911345	-0.263764	C	0.534050	-0.896887	-0.275887	C	-0.559368	0.909901	-0.255952
C	-0.162347	0.067622	-1.448578	C	0.123890	-0.073564	-1.468159	C	-0.138311	0.094394	-1.453857
C	-0.177304	-1.330980	0.756611	C	0.155510	1.349308	0.717285	C	-0.160745	-1.347197	0.720274
C	-0.728152	0.099762	0.926819	C	0.670509	-0.080324	0.907291	C	-0.702577	0.077996	0.914843
C	1.159055	-0.625323	-1.174977	C	-1.178711	0.637296	-1.203668	C	1.174008	-0.608269	-1.190182
C	1.179411	-1.327621	0.059081	C	-1.190953	1.353515	0.021132	C	1.191088	-1.330856	0.026342
C	-1.082800	-1.971705	-0.313077	C	1.078235	1.942029	-0.350878	C	-1.064376	-1.954758	-0.359779
C	-1.353817	-0.922650	-1.333360	C	1.332014	0.875931	-1.343435	C	-1.340752	-0.869446	-1.340679
C	2.357830	-0.575462	-1.945439	C	-2.401027	0.563741	-1.931612	C	2.387881	-0.523716	-1.932055
C	2.395490	-1.954689	0.463586	C	-2.421091	1.951661	0.420405	C	2.416623	-1.937515	0.428781
C	-1.288882	2.111125	-0.421071	C	1.269956	-2.082469	-0.408722	C	-1.281532	2.098822	-0.390993
C	-1.581316	0.581257	1.948123	C	1.526347	-0.579217	1.920417	C	-1.568433	0.549613	1.930740
C	-1.515500	-3.241423	-0.314583	C	1.545808	3.197267	-0.370303	C	-1.501401	-3.214962	-0.404184
C	-2.545298	-0.674605	-1.989243	C	2.536255	0.577392	-1.968173	C	-2.538251	-0.589347	-1.970771
H	-1.318306	2.576388	-1.403370	H	1.309316	-2.559592	-1.384810	H	-1.310178	2.573769	-1.367884
H	-2.586913	0.049656	-2.799690	H	2.561283	-0.152130	-2.774569	H	-2.572705	0.148989	-2.767534
H	-1.274961	-3.920101	0.500466	H	1.324740	3.892457	0.434055	H	-1.261813	-3.918973	0.387692
H	2.396383	0.117694	-2.784629	H	-2.459543	-0.135792	-2.763217	H	2.423422	0.186950	-2.755675
H	2.456067	-2.329852	1.484211	H	-2.489939	2.342403	1.433669	H	2.467277	-2.330498	1.442370
H	-0.170688	0.621908	-2.390281	H	0.129539	-0.645551	-2.399032	H	-0.142194	0.666719	-2.382626
H	-0.165441	-1.894056	1.693648	H	0.153167	1.926923	1.645177	H	-0.152229	-1.926412	1.645438
Fe	-2.583483	0.388955	0.039069	Fe	2.502731	-0.373655	0.040958	Fe	-2.518411	0.378196	0.037264
C	-3.910811	1.543701	-0.132130	C	3.839543	-1.527563	-0.088419	C	-3.861576	1.514693	-0.102873
C	-3.627925	-0.864042	0.748013	C	3.503700	0.908753	0.732714	C	-3.534884	-0.905460	0.739141
O	-4.332043	-1.632778	1.257300	O	4.175558	1.715687	1.236004	O	-4.213623	-1.685664	1.248982
O	-4.760393	2.331735	-0.243640	O	4.689770	-2.320401	-0.179871	O	-4.713318	2.292124	-0.198859
Fe	2.721974	0.096374	0.038180	Fe	-2.667676	-0.096852	0.044139	Fe	2.678367	0.098215	0.043641
C	2.591463	1.817492	-0.476073	C	-2.454889	-1.803858	-0.465405	C	2.496334	1.816538	-0.447611
C	2.568653	0.535131	1.778078	C	-2.403066	-0.503334	1.772161	C	2.453060	0.494839	1.781600
C	4.488855	-0.144646	-0.098121	C	-4.450694	0.067659	-0.028562	C	4.448419	-0.095478	-0.058401
O	2.489203	0.797717	2.902265	O	-2.221539	-0.742101	2.893634	O	2.306187	0.718805	2.900622
O	5.626537	-0.332552	-0.201072	O	-5.598629	0.218977	-0.097749	O	5.583090	-0.268954	-0.149843
O	2.534491	2.918266	-0.828983	O	-2.315496	-2.898236	-0.827759	O	2.388626	2.908133	-0.795905
H	-2.135844	-3.628436	-1.119950	H	2.193208	3.545577	-1.170267	H	-2.122978	-3.565761	-1.223490
H	-3.362880	-1.388980	-1.940834	H	3.353142	1.293514	-1.945629	H	-3.352491	-1.307847	-1.945428
H	2.966729	-2.538459	-0.256386	H	-3.004887	2.507201	-0.312372	H	2.991850	-2.512616	-0.294166
H	2.941197	-1.482643	-2.092921	H	-2.992742	1.468070	-2.066430	H	2.971771	-1.425579	-2.105045
H	-1.834620	-0.084264	2.771452	H	1.783591	0.076072	2.749647	H	-1.819156	-0.130118	2.742350
H	-1.453287	2.779808	0.417831	H	1.403341	-2.744771	0.440914	H	-1.427443	2.762751	0.454476
H	-1.592614	1.637914	2.205646	H	1.494721	-1.636599	2.177158	H	-1.550928	1.597615	2.219710

Table S4:Optimized coordinates for the **(Hericene)Fe$_m$(CO)$_n$** structure **2-5S-2.**

	B3LYP				M06L				wB97XD		
	x	y	z		x	y	z		x	y	z
C	1.327796	-1.500583	0.412104	C	-1.312326	-1.523970	-0.431714	C	1.313943	-1.502480	0.461996
C	0.250965	-1.225272	-0.630552	C	-0.228589	-1.280768	0.596380	C	0.238469	-1.266786	-0.580679
C	0.142084	0.523062	1.305478	C	-0.113573	0.477234	-1.325477	C	0.124021	0.533829	1.302509
C	1.351962	-0.448667	1.411296	C	-1.324675	-0.474833	-1.429631	C	1.334621	-0.424609	1.427243
C	-1.062014	-1.254737	0.122287	C	1.075093	-1.304474	-0.150956	C	-1.071478	-1.267864	0.164966
C	-1.109378	-0.351794	1.214400	C	1.127592	-0.397004	-1.238154	C	-1.123139	-0.339075	1.228585
C	0.343449	1.217300	-0.045805	C	-0.311267	1.152535	0.027146	C	0.320551	1.185221	-0.064526
C	0.786460	0.174992	-1.008877	C	-0.758133	0.110276	0.972761	C	0.785453	0.114265	-0.985118
C	-2.180873	-2.114995	-0.103101	C	2.217009	-2.124027	0.087901	C	-2.205578	-2.105436	-0.058018
C	-2.285567	-0.332224	2.022382	C	2.330205	-0.372310	-2.004770	C	-2.318321	-0.287292	2.005457
C	2.446756	-2.293842	0.145367	C	-2.474541	-2.250673	-0.143124	C	2.455419	-2.262461	0.194603
C	2.560287	-0.261051	2.124253	C	-2.555827	-0.272744	-2.103287	C	2.560414	-0.204531	2.102522
C	0.197876	2.529414	-0.282169	C	-0.161108	2.458762	0.283524	C	0.163480	2.479033	-0.352030
C	1.735618	0.312588	-2.002711	C	-1.720276	0.244864	1.962828	C	1.742056	0.225230	-1.973988
H	2.445820	-2.920727	-0.742948	H	-2.487596	-2.875463	0.746167	H	2.450419	-2.905759	-0.681073
H	1.910048	-0.491124	-2.714441	H	-1.885046	-0.560999	2.674249	H	1.911038	-0.597933	-2.662979
H	-0.086445	3.219096	0.508496	H	0.120390	3.159796	-0.496160	H	-0.127792	3.193798	0.411754
H	-2.210000	-2.671983	-1.038691	H	2.253875	-2.684179	1.020172	H	-2.223896	-2.681367	-0.981413
H	-2.402553	0.488531	2.728991	H	2.464015	0.440505	-2.716149	H	-2.431919	0.551381	2.690087
H	0.283078	-1.925584	-1.468609	H	-0.267399	-1.982814	1.432417	H	0.269934	-1.987118	-1.399260
H	0.097392	1.228318	2.139801	H	-0.065890	1.194574	-2.148964	H	0.078175	1.261290	2.115199
Fe	2.825969	-0.127162	-0.019353	Fe	-2.744100	-0.118982	0.007470	Fe	2.758737	-0.131719	-0.009435
C	4.443042	-0.615523	-0.535321	C	-4.376581	-0.552442	0.532585	C	4.382694	-0.588760	-0.523582
C	3.298515	1.575670	0.177173	C	-3.131865	1.594771	-0.183176	C	3.190523	1.587816	0.162210
O	3.659940	2.664633	0.347218	O	-3.431459	2.706563	-0.353347	O	3.528339	2.677938	0.324279
O	5.502688	-0.972908	-0.859603	O	-5.443191	-0.884080	0.868660	O	5.439315	-0.930976	-0.849235
Fe	-2.810857	-0.102257	-0.021842	Fe	2.752219	-0.102610	0.031046	Fe	-2.763415	-0.101273	-0.026518
C	-3.010803	1.672724	0.210702	C	2.917672	1.660238	-0.250829	C	-2.947646	1.671944	0.189420
C	-2.733430	0.071917	-1.812629	C	2.496798	0.136212	1.792540	C	-2.582496	0.056226	-1.807447
C	-4.505798	-0.616862	0.213851	C	4.474924	-0.578386	-0.068027	C	-4.467416	-0.591224	0.149537
O	-2.693263	0.167503	-2.965584	O	2.300851	0.291619	2.926680	O	-2.447755	0.139211	-2.947596
O	-5.591531	-0.982932	0.380876	O	5.576612	-0.933241	-0.149777	O	-5.551242	-0.954702	0.289726
O	-3.184497	2.802202	0.390252	O	3.058765	2.790390	-0.473223	O	-3.105812	2.796931	0.371189
H	0.364172	2.950101	-1.271288	H	-0.336409	2.860681	1.277855	H	0.330359	2.855782	-1.357382
H	2.095433	1.296767	-2.290709	H	-2.040315	1.234627	2.276668	H	2.081737	1.203831	-2.300629
H	-2.716841	-1.272338	2.363228	H	2.771905	-1.316784	-2.321836	H	-2.751479	-1.214810	2.375784
H	-2.628801	-2.642094	0.737759	H	2.695311	-2.624025	-0.753047	H	-2.663024	-2.617113	0.786500
H	2.664150	0.624586	2.748695	H	-2.663323	0.608317	-2.732204	H	2.656010	0.698321	2.702172
H	3.121352	-2.602119	0.937778	H	-3.151864	-2.549019	-0.937221	H	3.129642	-2.560116	0.990823
H	3.141914	-1.117228	2.458979	H	-3.134570	-1.133414	-2.434431	H	3.136231	-1.047868	2.476332

Table S5:Optimized coordinates for the **(Hericene)Fe$_m$(CO)$_n$** structure **2-5S-3.**

	B3LYP				M06L				wB97XD		
	x	y	z		x	y	z		x	y	z
C	1.192051	1.250700	0.729608	C	0.744780	1.173659	1.239247	C	-1.173350	-1.242602	0.728280
C	1.816692	0.000011	1.349298	C	1.360619	1.799860	0.000000	C	-1.798421	0.000001	1.348689
C	1.925981	0.000000	-1.256500	C	-1.244848	1.891882	0.000000	C	-1.902682	0.000003	-1.253733
C	1.252605	1.241027	-0.701323	C	-0.681469	1.226539	1.230229	C	-1.231646	-1.233453	-0.695603
C	1.192056	-1.250687	0.729619	C	0.744780	1.173659	-1.239247	C	-1.173345	1.242603	0.728281
C	1.252610	-1.241025	-0.701312	C	-0.681469	1.226539	-1.230229	C	-1.231643	1.233456	-0.695602
C	3.335755	0.000006	-0.635771	C	-0.630482	3.293529	0.000000	C	-3.306791	0.000005	-0.637450
C	3.274972	0.000010	0.856565	C	0.850990	3.242360	0.000000	C	-3.248927	0.000004	0.855107
C	0.580164	-2.356153	1.364068	C	1.349766	0.541551	-2.353611	C	-0.532145	2.343571	1.344687
C	0.666427	-2.260778	-1.464083	C	-1.407440	0.610526	-2.264139	C	-0.613562	2.242901	-1.447440
C	0.580156	2.356171	1.364047	C	1.349766	0.541551	2.353611	C	-0.532154	-2.343574	1.344684
C	0.666414	2.260768	-1.464101	C	-1.407440	0.610526	2.264139	C	-0.613567	-2.242899	-1.447442
C	4.446228	0.000007	-1.390759	C	-1.383785	4.401918	0.000000	C	-4.413357	0.000007	-1.386861
C	4.323756	0.000012	1.695449	C	1.673932	4.300416	0.000000	C	-4.296849	0.000007	1.684759
H	0.407254	2.309337	2.437783	H	2.421680	0.358109	2.327697	H	-0.364998	-2.296690	2.418787
H	4.180958	0.000016	2.773757	H	2.752552	4.173691	0.000000	H	-4.155220	0.000007	2.762149
H	4.389376	0.000003	-2.476993	H	-2.468383	4.345649	0.000000	H	-4.354547	0.000007	-2.471911
H	0.407257	-2.309309	2.437802	H	2.421680	0.358109	-2.327697	H	-0.364989	2.296685	2.418789
H	0.560197	-2.110744	-2.536725	H	-2.479006	0.473971	-2.133114	H	-0.499139	2.082225	-2.517016
H	1.754874	0.000015	2.441095	H	2.452294	1.748667	0.000000	H	-1.732679	0.000000	2.438626
H	1.964464	-0.000005	-2.349213	H	-2.337755	1.919092	0.000000	H	-1.934048	0.000003	-2.345081
Fe	-0.756982	1.383517	-0.029554	Fe	-0.014224	-0.746105	1.355429	Fe	0.738918	-1.356490	-0.026368
C	-1.785620	2.625231	-0.749640	C	-0.760853	-1.832419	2.532967	C	1.795790	-2.566312	-0.747067
C	-1.836951	1.348016	1.390428	C	1.409352	-1.813363	1.309170	C	1.801711	-1.330519	1.405536
O	-2.522460	1.502451	2.313006	O	2.342457	-2.489899	1.465610	O	2.472674	-1.494741	2.329171
O	-2.443720	3.450042	-1.233622	O	-1.279759	-2.519972	3.314624	O	2.461956	-3.372871	-1.235468
Fe	-0.756975	-1.383521	-0.029547	Fe	-0.014224	-0.746105	-1.355429	Fe	0.738923	1.356486	-0.026368
C	-1.836955	-1.348002	1.390427	C	1.409352	-1.813363	-1.309170	C	1.801721	1.330518	1.405532
C	-1.465547	-0.000013	-1.285497	C	-1.366685	-1.310918	0.000000	C	1.405690	-0.000003	-1.312118
C	-1.785594	-2.625258	-0.749621	C	-0.760853	-1.832419	-2.532967	C	1.795796	2.566304	-0.747072
O	-1.973624	-0.000012	-2.349170	O	-2.507438	-1.619839	0.000000	O	1.847639	-0.000004	-2.398403
O	-2.443679	-3.450084	-1.233596	O	-1.279759	-2.519972	-3.314624	O	2.461963	3.372862	-1.235474
O	-2.522472	-1.502425	2.313000	O	2.342457	-2.489899	-1.465610	O	2.472688	1.494747	2.329162
H	5.441794	0.000013	-0.951568	H	-0.941315	5.395003	0.000000	H	-5.406793	0.000008	-0.945642
H	5.351101	0.000011	1.336584	H	1.297155	5.320379	0.000000	H	-5.321272	0.000009	1.320988
H	0.701246	-3.298002	-1.143432	H	-1.070918	0.678008	-3.295649	H	-0.672144	3.283657	-1.142113
H	0.709690	-3.363264	0.972705	H	0.944910	0.699623	-3.352484	H	-0.687841	3.351269	0.964891
H	0.560185	2.110727	-2.536742	H	-2.479006	0.473971	2.133114	H	-0.499142	-2.082222	-2.517017
H	0.709679	3.363279	0.972676	H	0.944910	0.699623	3.352484	H	-0.687853	-3.351271	0.964887
H	0.701218	3.297996	-1.143457	H	-1.070918	0.678008	3.295649	H	-0.672153	-3.283655	-1.142116

Table S6:Optimized coordinates for the **(Hericene)Fe$_m$(CO)$_n$** structure **2-5S-4.**

	B3LYP				M06L				wB97XD	
	x	y	z		x	y	z		x	y	z
C	0.567206	2.297316	-0.341841	C	0.615426	2.253661	-0.344726	C	0.581947	2.272272	-0.331759
C	-0.160623	1.219023	-1.155369	C	-0.156131	1.216510	-1.152200	C	-0.160090	1.216149	-1.148909
C	-0.244613	0.695894	1.386072	C	-0.240492	0.703596	1.387947	C	-0.247379	0.675616	1.383823
C	0.514780	1.999597	1.116987	C	0.562922	1.964748	1.103745	C	0.526194	1.965089	1.124617
C	0.412484	-0.154412	-0.816496	C	0.359817	-0.169645	-0.814024	C	0.399467	-0.158796	-0.819339
C	0.379975	-0.435706	0.571561	C	0.333205	-0.443871	0.575110	C	0.367519	-0.447894	0.562127
C	-1.638826	0.937889	0.785175	C	-1.619645	0.978663	0.792651	C	-1.632428	0.932044	0.784607
C	-1.592263	1.268589	-0.594139	C	-1.572817	1.303548	-0.586467	C	-1.584106	1.268693	-0.587442
C	0.958691	-1.136686	-1.699490	C	0.885020	-1.179171	-1.675173	C	0.951513	-1.139513	-1.697750
C	0.914034	-1.679179	1.028656	C	0.858279	-1.695699	1.011892	C	0.906656	-1.693800	1.001321
C	1.128184	3.371042	-0.920444	C	1.223719	3.298067	-0.923554	C	1.169437	3.331068	-0.896788
C	1.011170	2.760892	2.105052	C	1.107925	2.706110	2.077787	C	1.045339	2.704214	2.109240
C	-2.928947	0.818667	1.415978	C	-2.921317	0.854018	1.390912	C	-2.925895	0.793056	1.398451
C	-2.778003	1.447294	-1.352854	C	-2.773842	1.455318	-1.326308	C	-2.776139	1.436489	-1.340334
H	1.106764	3.507161	-1.999211	H	1.211009	3.432377	-2.001305	H	1.154957	3.471790	-1.974001
H	-2.692897	1.471363	-2.438734	H	-2.705935	1.486196	-2.412332	H	-2.682501	1.466116	-2.424778
H	-2.959349	0.379906	2.413186	H	-2.980626	0.414514	2.385386	H	-2.953960	0.347770	2.392144
H	1.150913	-0.843029	-2.730695	H	1.078595	-0.915882	-2.713369	H	1.141452	-0.839212	-2.726679
H	1.062426	-1.801983	2.100677	H	1.015450	-1.833962	2.079935	H	1.049783	-1.822614	2.072494
H	-0.131342	1.432173	-2.227525	H	-0.121083	1.428062	-2.224530	H	-0.129063	1.434164	-2.218530
H	-0.289484	0.464623	2.453988	H	-0.284192	0.476568	2.456759	H	-0.295257	0.433576	2.447626
Fe	-2.897483	-0.349358	-0.241802	Fe	-2.813242	-0.321492	-0.237260	Fe	-2.842657	-0.343216	-0.263077
C	-2.647899	-1.843117	0.709191	C	-2.515377	-1.774157	0.739757	C	-2.536309	-1.820443	0.701683
C	-4.616974	-0.611372	-0.611692	C	-4.518011	-0.647357	-0.614108	C	-4.566239	-0.616168	-0.593104
O	-5.764289	-0.705246	-0.761889	O	-5.665644	-0.772997	-0.766322	O	-5.710419	-0.701701	-0.716198
O	-2.509334	-2.822698	1.315715	O	-2.334484	-2.731871	1.379303	O	-2.366373	-2.762598	1.344510
Fe	2.346612	-0.682342	-0.166201	Fe	2.262925	-0.692676	-0.172253	Fe	2.294974	-0.676474	-0.171438
C	3.185776	-0.045848	1.295025	C	3.082204	-0.027296	1.279506	C	3.108948	-0.036238	1.297117
C	3.273777	-2.185560	-0.437496	C	3.209691	-2.188933	-0.434376	C	3.235345	-2.164119	-0.448328
C	3.238754	0.446301	-1.250390	C	3.117138	0.443492	-1.268305	C	3.162872	0.471983	-1.247174
O	3.843201	-3.177795	-0.617457	O	3.779441	-3.184377	-0.609598	O	3.793808	-3.154272	-0.633907
O	3.827033	1.137008	-1.968931	O	3.669917	1.151418	-2.004113	O	3.720688	1.176759	-1.965904
O	3.740629	0.322305	2.241412	O	3.617248	0.367442	2.231068	O	3.632678	0.333439	2.252657
H	-3.670244	1.607805	1.273074	H	-3.658267	1.642424	1.221568	H	-3.662644	1.586746	1.267069
H	-3.604715	2.034898	-0.956226	H	-3.593206	2.042621	-0.913937	H	-3.590815	2.042729	-0.948650
H	0.641259	-2.600437	0.516244	H	0.558006	-2.603863	0.489566	H	0.615623	-2.609281	0.489300
H	0.671159	-2.180959	-1.583373	H	0.571931	-2.212938	-1.526156	H	0.644837	-2.179232	-1.593305
H	0.917285	2.463811	3.147056	H	1.021890	2.416486	3.121070	H	0.952560	2.397846	3.147507
H	1.632680	4.140135	-0.339391	H	1.768307	4.036837	-0.341124	H	1.689203	4.081923	-0.307551
H	1.530180	3.696412	1.907284	H	1.668272	3.612387	1.862692	H	1.582609	3.628386	1.913132

Table S7:Optimized coordinates for the **(Hericene)Fe$_m$(CO)$_n$** structure **2-5S-5.**

	B3LYP				M06L				wB97XD		
	x	y	z		x	y	z		x	y	z
C	-1.047539	1.160254	-0.709957	C	1.011976	1.193775	0.709719	C	-1.033754	1.169249	-0.707468
C	0.317299	0.784871	-1.300241	C	-0.329299	0.775445	1.299148	C	0.319881	0.779621	-1.297929
C	0.317297	0.784788	1.300280	C	-0.329295	0.775364	-1.299198	C	0.319885	0.779630	1.297933
C	-1.047538	1.160210	0.710016	C	1.011978	1.193730	-0.709791	C	-1.033755	1.169248	0.707474
C	1.248375	1.872191	-0.744998	C	-1.297680	1.812705	0.739301	C	1.264933	1.845932	-0.745002
C	1.248373	1.872147	0.745111	C	-1.297678	1.812659	-0.739419	C	1.264932	1.845938	0.744993
C	0.720677	-0.564986	0.714147	C	-0.670198	-0.580784	-0.712319	C	0.700459	-0.569323	0.709323
C	0.720678	-0.564942	-0.714195	C	-0.670200	-0.580739	0.712352	C	0.700454	-0.569327	-0.709309
C	1.918678	2.716607	-1.544595	C	-2.009328	2.626242	1.530939	C	1.959302	2.667987	-1.536744
C	1.918661	2.716524	1.544761	C	-2.009322	2.626147	-1.531111	C	1.959294	2.668005	1.536728
C	-2.251662	1.512459	-1.388643	C	2.226370	1.544810	1.365887	C	-2.245804	1.506808	-1.376772
C	-2.251658	1.512379	1.388727	C	2.226374	1.544723	-1.365978	C	-2.245807	1.506806	1.376775
C	1.126552	-1.717482	1.418448	C	-1.034937	-1.772001	-1.383023	C	1.086697	-1.743819	1.396373
C	1.126562	-1.717391	-1.418565	C	-1.034940	-1.771915	1.383130	C	1.086683	-1.743829	-1.396356
H	-2.302253	1.329875	-2.461236	H	2.295822	1.368847	2.437603	H	-2.291605	1.322718	-2.448540
H	1.270303	-1.648019	-2.494687	H	-1.185562	-1.730330	2.459685	H	1.230180	-1.676260	-2.472470
H	1.270272	-1.648176	2.494577	H	-1.185557	-1.730483	-2.459581	H	1.230203	-1.676247	2.472485
H	1.850209	2.642238	-2.627404	H	-1.949515	2.553477	2.612945	H	1.894096	2.590844	-2.618473
H	1.850187	2.642088	2.627565	H	-1.949504	2.553314	-2.613112	H	1.894090	2.590871	2.618458
H	0.311146	0.776504	-2.393695	H	-0.325870	0.767510	2.392782	H	0.311693	0.767571	-2.389852
H	0.311137	0.776347	2.393734	H	-0.325862	0.767360	-2.392831	H	0.311704	0.767594	2.389857
Fe	2.598024	-1.173326	-0.000036	Fe	-2.494210	-1.208004	0.000035	Fe	2.529560	-1.199894	0.000002
C	3.654682	-0.507675	-1.277908	C	-3.495453	-0.483911	1.283929	C	3.541243	-0.482630	-1.289694
C	3.654701	-0.507810	1.277895	C	-3.495453	-0.483993	-1.283906	C	3.541241	-0.482622	1.289696
O	4.323460	-0.050074	2.110562	O	-4.116521	0.021763	-2.130751	O	4.160230	0.002756	2.134304
O	4.323404	-0.049848	-2.110556	O	-4.116521	0.021899	2.130742	O	4.160235	0.002740	-2.134306
Fe	-2.622367	-0.047660	0.000000	Fe	2.546932	-0.015140	0.000005	Fe	-2.565519	-0.031419	0.000001
C	-4.381618	0.268199	0.000011	C	4.316707	0.255045	-0.000002	C	-4.325082	0.250259	-0.000003
C	-2.514922	-1.291394	-1.297254	C	2.389748	-1.246345	1.295907	C	-2.418714	-1.265041	-1.296788
C	-2.514939	-1.291471	1.297181	C	2.389749	-1.246429	-1.295819	C	-2.418727	-1.265046	1.296784
O	-2.479513	-2.077562	-2.146145	O	2.307896	-2.025027	2.153528	O	-2.346058	-2.034196	-2.150060
O	-2.479549	-2.077690	2.146025	O	2.307906	-2.025163	-2.153393	O	-2.346085	-2.034215	2.150046
O	-5.514277	0.508792	0.000022	O	5.454437	0.482305	-0.000014	O	-5.452118	0.487958	-0.000007
H	0.857390	-2.713722	1.061147	H	-0.658738	-2.730029	-1.016940	H	0.730533	-2.717178	1.055041
H	0.857416	-2.713655	-1.061322	H	-0.658740	-2.729965	1.017107	H	0.730515	-2.717187	-1.055024
H	2.565235	3.495804	1.147014	H	-2.690066	3.369391	-1.124163	H	2.621614	3.430170	1.134870
H	2.565260	3.495855	-1.146797	H	-2.690071	3.369460	1.123942	H	2.621626	3.430151	-1.134892
H	-2.302248	1.329736	2.461310	H	2.295829	1.368693	-2.437682	H	-2.291608	1.322715	2.448543
H	-2.817632	2.383496	-1.061618	H	2.782371	2.415601	1.019998	H	-2.803957	2.385475	-1.058535
H	-2.817629	2.383434	1.061751	H	2.782374	2.415537	-1.020142	H	-2.803954	2.385479	1.058541

Table S8:Optimized coordinates for the **(Hericene)Fe$_m$(CO)$_n$** structure **2-5T-6.**

	B3LYP				M06L				wB97XD	
	x	y	z		x	y	z		x	y	z
C	0.708579	-0.555893	-0.558402	C	0.627651	-0.655325	-0.496710	C	0.784803	-0.566176	0.519300
C	0.176031	0.559472	-1.459430	C	0.172399	0.392668	-1.497270	C	0.375019	-0.659595	-0.947201
C	0.134176	1.223101	1.046259	C	0.137752	1.272141	0.939040	C	-0.075253	1.699318	0.055724
C	0.699918	-0.181679	0.836459	C	0.634960	-0.160490	0.847488	C	0.345274	0.709911	1.132211
C	-1.227240	0.936180	-0.976891	C	-1.203299	0.878239	-1.070314	C	-1.007540	-0.147130	-1.253057
C	-1.251197	1.291706	0.396519	C	-1.226205	1.346032	0.268338	C	-1.267584	1.127914	-0.702720
C	0.949977	2.229053	0.221194	C	1.006826	2.149787	0.043578	C	1.034671	1.738418	-0.996413
C	1.008287	1.824978	-1.212266	C	1.094848	1.597022	-1.325722	C	1.429889	0.360716	-1.385432
C	-2.449344	0.978075	-1.711336	C	-2.433874	0.876555	-1.787723	C	-2.057769	-0.800839	-1.963450
C	-2.496024	1.676998	0.978022	C	-2.477335	1.783018	0.791377	C	-2.569515	1.681195	-0.887640
C	1.234758	-1.771913	-1.025446	C	1.195033	-1.903629	-0.814261	C	1.515186	-1.581621	1.131117
C	1.241969	-0.950069	1.859136	C	1.153873	-0.902692	1.913682	C	0.291672	0.975449	2.442061
C	1.468483	3.343846	0.759832	C	1.549294	3.298586	0.468945	C	1.520710	2.869791	-1.518566
C	1.683781	2.447111	-2.192581	C	1.898560	2.036302	-2.305658	C	2.589742	-0.027688	-2.052317
H	1.313668	-1.934219	-2.098150	H	1.303534	-2.177078	-1.860801	H	1.480666	-2.593992	0.735453
H	1.656729	2.079320	-3.215904	H	1.895800	1.571652	-3.287398	H	2.614532	-0.962219	-2.608137
H	1.369207	3.556159	1.821848	H	1.437517	3.627894	1.497775	H	1.181597	3.839842	-1.166007
H	-2.462762	0.525861	-2.702124	H	-2.469572	0.348431	-2.738578	H	-1.911970	-1.849470	-2.216161
H	-2.544625	1.764497	2.062654	H	-2.546558	1.964664	1.862157	H	-2.820046	2.567253	-0.306973
H	0.188860	0.268290	-2.513447	H	0.186413	0.010366	-2.521592	H	0.562215	-1.652600	-1.363330
H	0.109674	1.497001	2.104475	H	0.116337	1.635205	1.970137	H	-0.288841	2.688553	0.466204
Fe	2.727315	-0.751591	0.123749	Fe	2.611698	-0.740434	0.233681	Fe	2.925847	-0.435956	0.012079
C	4.117971	-1.827993	-0.375151	C	4.011162	-1.663495	-0.448223	C	4.329709	-1.607254	-0.075782
C	3.814084	0.607964	0.724316	C	3.627440	0.590770	0.929068	C	3.359673	1.200454	0.937523
O	4.554680	1.403116	1.134142	O	4.318804	1.410012	1.388062	O	3.540107	2.201475	1.466893
O	5.023786	-2.476236	-0.699793	O	4.912915	-2.221918	-0.927373	O	5.177961	-2.382515	-0.161032
Fe	-2.680797	-0.210277	0.031282	Fe	-2.628673	-0.163048	0.024595	Fe	-2.692852	-0.230272	-0.059291
C	-2.418367	-1.723318	-0.909074	C	-2.322504	-1.731630	-0.791195	C	-2.282664	-1.841346	0.622383
C	-2.470540	-1.068208	1.600708	C	-2.367174	-0.875621	1.651136	C	-2.922114	0.486069	1.571497
C	-4.463140	-0.092019	-0.033833	C	-4.416978	-0.083068	-0.034451	C	-4.392968	-0.454895	-0.541228
O	-2.372268	-1.606087	2.620788	O	-2.213024	-1.315841	2.714356	O	-3.111563	0.973312	2.596595
O	-5.614403	0.020532	-0.085282	O	-5.571271	0.018443	-0.090308	O	-5.478588	-0.582085	-0.905113
O	-2.281831	-2.690012	-1.530960	O	-2.133367	-2.735665	-1.343185	O	-2.015299	-2.882821	1.033890
H	2.016420	4.070316	0.163626	H	2.138013	3.930616	-0.190868	H	2.265599	2.856124	-2.310145
H	2.288827	3.331415	-2.004197	H	2.588880	2.861490	-2.150101	H	3.302685	0.730908	-2.370990
H	-3.141553	2.376813	0.449265	H	-3.104283	2.437357	0.186559	H	-3.017943	1.667908	-1.879399
H	-3.108140	1.839205	-1.609455	H	-3.074445	1.756551	-1.741444	H	-2.625425	-0.244786	-2.706990
H	1.312599	-0.549486	2.866291	H	1.254232	-0.445461	2.892839	H	-0.026726	1.948746	2.804025
H	1.154510	-2.684673	-0.435180	H	1.066300	-2.750595	-0.139234	H	1.769767	-1.495775	2.185937
H	1.384446	-2.026773	1.760111	H	1.162158	-1.991862	1.886192	H	0.549422	0.226385	3.186109

Table S9: Optimized coordinates for the **(Hericene)Fe$_m$(CO)$_n$** structure **2-5T-7.**

	B3LYP				M06L				wB97XD		
	x	y	z		x	y	z		x	y	z
C	0.478199	2.161585	-0.745311	C	0.607176	2.242618	-0.427998	C	0.493895	2.129102	-0.745169
C	-0.237256	0.920762	-1.297084	C	-0.125854	1.169434	-1.223546	C	-0.234363	0.902870	-1.294206
C	-0.237254	0.920767	1.297084	C	-0.304146	0.749568	1.326526	C	-0.234361	0.902885	1.294211
C	0.478199	2.161589	0.745306	C	0.501597	2.006052	1.027212	C	0.493857	2.129133	0.745155
C	0.401251	-0.331594	-0.708187	C	0.385731	-0.198974	-0.814050	C	0.393553	-0.347800	-0.705820
C	0.401252	-0.331591	0.708191	C	0.306871	-0.421249	0.582230	C	0.393558	-0.347787	0.705837
C	-1.656332	1.064359	0.720259	C	-1.666482	1.026160	0.689925	C	-1.644786	1.058875	0.717418
C	-1.656332	1.064357	-0.720256	C	-1.565036	1.284087	-0.728824	C	-1.644782	1.058877	-0.717409
C	0.996932	-1.435221	-1.393399	C	0.957345	-1.232456	-1.614073	C	1.001512	-1.449222	-1.380921
C	0.996933	-1.435215	1.393406	C	0.838248	-1.642508	1.090573	C	1.001522	-1.449194	1.380957
C	0.981760	3.115444	-1.544453	C	1.225542	3.272499	-1.022225	C	1.018133	3.068769	-1.537184
C	0.981754	3.115454	1.544443	C	1.004117	2.785029	1.994822	C	1.017923	3.068907	1.537155
C	-2.838814	1.194534	1.473944	C	-2.897093	1.040473	1.350098	C	-2.829571	1.183128	1.467178
C	-2.838817	1.194532	-1.473939	C	-2.679723	1.456014	-1.545635	C	-2.829563	1.183134	-1.467174
H	0.923759	3.032688	-2.627320	H	1.248568	3.368398	-2.103904	H	0.963572	2.983240	-2.618940
H	-2.792789	1.024207	-2.548170	H	-2.544833	1.496777	-2.623238	H	-2.776597	1.010357	-2.539641
H	-2.792784	1.024210	2.548175	H	-2.946587	0.716303	2.386047	H	-2.776605	1.010345	2.539644
H	1.166293	-1.332622	-2.464697	H	1.188400	-1.006038	-2.653242	H	1.167294	-1.344640	-2.451747
H	1.166296	-1.332612	2.464704	H	0.959708	-1.730613	2.168588	H	1.167312	-1.344588	2.451779
H	-0.242062	0.911616	-2.390590	H	-0.048718	1.339862	-2.300905	H	-0.239609	0.890702	-2.386205
H	-0.242057	0.911625	2.390591	H	-0.386354	0.565740	2.401047	H	-0.239599	0.890731	2.386209
Fe	-3.083400	-0.364770	0.000002	Fe	-2.841551	-0.399229	-0.372282	Fe	-3.034287	-0.344907	-0.000005
C	-2.193431	-2.037701	0.000000	C	-2.370797	-2.041930	0.255212	C	-2.135257	-2.020466	0.000034
C	-4.895241	-0.679687	-0.000003	C	-4.662410	-0.518524	-0.078739	C	-4.835317	-0.675749	-0.000041
O	-6.037472	-0.862311	-0.000006	O	-5.804901	-0.622948	0.103134	O	-5.972401	-0.849703	-0.000072
O	-1.740648	-3.101586	-0.000001	O	-2.113999	-3.098704	0.679948	O	-1.681402	-3.075892	0.000071
Fe	2.372009	-0.634662	0.000001	Fe	2.270866	-0.664312	-0.081928	Fe	2.326724	-0.629740	0.000004
C	3.205045	0.297742	1.296639	C	3.033187	0.064729	1.370239	C	3.133881	0.316267	1.297320
C	3.355184	-2.125324	0.000007	C	3.240581	-2.158629	-0.251518	C	3.327403	-2.101999	0.000039
C	3.205044	0.297729	-1.296647	C	3.153130	0.435414	-1.192092	C	3.133888	0.316189	-1.297363
O	3.962255	-3.111847	0.000011	O	3.826223	-3.153387	-0.368058	O	3.926039	-3.086354	0.000064
O	3.759858	0.856539	-2.144949	O	3.726524	1.118397	-1.935981	O	3.659174	0.881801	-2.151024
O	3.759860	0.856562	2.144933	O	3.533071	0.500138	2.323433	O	3.659162	0.881938	2.150945
H	-3.639917	1.859332	1.153196	H	-3.689692	1.711400	1.028466	H	-3.618348	1.865887	1.155661
H	-3.639918	1.859333	-1.153192	H	-3.585903	1.921921	-1.166058	H	-3.618342	1.865890	-1.155653
H	0.760674	-2.450676	1.080410	H	0.578166	-2.577863	0.597403	H	0.753700	-2.463733	1.074624
H	0.760672	-2.450680	-1.080399	H	0.646216	-2.262572	-1.438586	H	0.753688	-2.463754	-1.074565
H	0.923753	3.032704	2.627311	H	0.875307	2.533429	3.043551	H	0.963307	2.983424	2.618913
H	1.472483	4.001053	-1.146278	H	1.743327	4.036891	-0.448582	H	1.522926	3.943455	-1.135603
H	1.472472	4.001064	1.146263	H	1.572939	3.683625	1.770249	H	1.522622	3.943642	1.135562

Table S10:Optimized coordinates for the (**Hericene)Fe$_m$(CO)$_n$** structure **2-5T-8.**

	B3LYP				M06L				wB97XD		
	x	y	z		x	y	z		x	y	z
C	-1.374508	-1.239843	0.160637	C	-1.343698	-1.374236	-0.094707	C	-1.412973	-0.961319	0.717236
C	-0.232448	-0.717817	1.052474	C	-0.226919	-0.992074	0.874759	C	-0.212326	-0.199695	1.292590
C	-0.132515	0.292626	-1.331010	C	-0.136334	0.393070	-1.308772	C	-0.212336	-0.199697	-1.292579
C	-1.329430	-0.661787	-1.168897	C	-1.312212	-0.584853	-1.294408	C	-1.412980	-0.961319	-0.717215
C	1.016216	-1.158172	0.282251	C	1.033984	-1.256243	0.068519	C	0.973035	-0.965637	0.706762
C	1.069415	-0.608092	-1.023963	C	1.081329	-0.505088	-1.132243	C	0.973029	-0.965638	-0.706759
C	-0.228066	1.386581	-0.269645	C	-0.257190	1.290008	-0.088741	C	-0.199893	1.216081	-0.743602
C	-0.310335	0.805541	1.096783	C	-0.374876	0.495618	1.149907	C	-0.199886	1.216084	0.743613
C	2.074597	-2.018449	0.702602	C	2.143628	-2.099484	0.364949	C	2.040558	-1.630025	1.378806
C	2.178657	-0.939974	-1.858799	C	2.237523	-0.648327	-1.953230	C	2.040545	-1.630027	-1.378811
C	-2.370820	-2.135190	0.578579	C	-2.424088	-2.231562	0.182702	C	-2.442620	-1.555644	1.471573
C	-2.288374	-0.861271	-2.146990	C	-2.318147	-0.651696	-2.261452	C	-2.442635	-1.555649	-1.471542
C	-0.225660	2.690759	-0.587315	C	-0.241381	2.626722	-0.176632	C	-0.194329	2.292428	-1.536383
C	-0.468801	1.482109	2.246465	C	-0.644442	0.970894	2.375202	C	-0.194313	2.292429	1.536396
H	-2.395908	-2.449085	1.619749	H	-2.486390	-2.694253	1.164280	H	-2.462062	-1.374373	2.543697
H	-0.518190	0.965557	3.202360	H	-0.709045	0.309248	3.234275	H	-0.189702	2.191094	2.618148
H	-0.167476	3.020500	-1.621929	H	-0.157513	3.128317	-1.136310	H	-0.189725	2.191098	-2.618136
H	2.126587	-2.281255	1.758401	H	2.204423	-2.530466	1.362413	H	2.134369	-1.465328	2.450689
H	2.309992	-0.371908	-2.778911	H	2.371392	0.055328	-2.772672	H	2.134347	-1.465335	-2.450695
H	-0.276128	-1.156404	2.053298	H	-0.268202	-1.580504	1.795340	H	-0.211731	-0.212347	2.384610
H	-0.088522	0.710836	-2.340351	H	-0.096663	0.971846	-2.235595	H	-0.211749	-0.212346	-2.384598
Fe	-3.279677	-0.316752	-0.113195	Fe	-3.152695	-0.289037	-0.212440	Fe	-3.262082	-0.256243	0.000008
C	-4.957637	-0.692413	0.507571	C	-4.673787	-0.586749	0.716871	C	-5.035222	-0.692605	-0.000005
C	-3.582047	1.492512	-0.274487	C	-3.428017	1.496724	-0.380881	C	-3.159577	1.658670	-0.000003
O	-3.852932	2.616335	-0.383209	O	-3.665657	2.632203	-0.493661	O	-3.215137	2.804308	-0.000002
O	-6.027067	-0.892771	0.910940	O	-5.628748	-0.762212	1.358974	O	-6.143371	-1.004450	-0.000033
Fe	2.865083	-0.183726	-0.003291	Fe	2.784790	-0.136950	0.003962	Fe	2.790178	-0.255839	-0.000006
C	3.126578	1.449406	-0.717645	C	2.935241	1.592505	-0.452203	C	3.015049	0.966297	-1.296933
C	3.009916	0.455352	1.675425	C	2.781434	0.249233	1.758268	C	3.015064	0.966296	1.296918
C	4.492791	-0.887021	-0.221792	C	4.475171	-0.676799	-0.226031	C	4.390662	-1.036522	-0.000007
O	3.136766	0.838437	2.759842	O	2.798664	0.471346	2.897439	O	3.182615	1.719472	2.151035
O	5.531761	-1.374635	-0.376745	O	5.553034	-1.070128	-0.399291	O	5.400079	-1.592002	-0.000015
O	3.331245	2.481359	-1.199267	O	3.062827	2.699181	-0.778011	O	3.182586	1.719485	-2.151042
H	-0.283983	3.468611	0.170669	H	-0.314020	3.259144	0.704178	H	-0.189342	3.302413	-1.135309
H	-0.553175	2.566332	2.270665	H	-0.801228	2.031212	2.555034	H	-0.189329	3.302416	1.135325
H	2.502730	-1.977282	-1.930605	H	2.616510	-1.648982	-2.159128	H	2.323036	-2.633030	-1.063640
H	2.423035	-2.804617	0.034282	H	2.547787	-2.736163	-0.421447	H	2.323043	-2.633030	1.063637
H	-2.254268	-0.296774	-3.074433	H	-2.327841	0.054169	-3.085547	H	-2.462083	-1.374364	-2.543664
H	-2.830434	-2.835169	-0.119218	H	-2.890741	-2.805449	-0.618471	H	-2.883430	-2.502137	1.163106
H	-2.978189	-1.705855	-2.121268	H	-2.927223	-1.546890	-2.380819	H	-2.883422	-2.502157	-1.163089

Table S11:Optimized coordinates for the **(Hericene)Fe$_m$(CO)$_n$** structure **2-5T-9.**

	B3LYP				M06L				wB97XD		
	x	y	z		x	y	z		x	y	z
C	-1.157601	1.186803	0.709732	C	-1.190934	1.168507	0.709306	C	-1.221624	1.165809	-0.707044
C	-0.861175	-0.195414	1.300249	C	-0.839013	-0.189395	1.298382	C	0.173600	0.963582	-1.293826
C	-0.861175	-0.195414	-1.300249	C	-0.839013	-0.189395	-1.298382	C	0.173601	0.963583	1.293826
C	-1.157601	1.186803	-0.709732	C	-1.190934	1.168507	-0.709306	C	-1.221623	1.165809	0.707048
C	-1.994200	-1.074423	0.746215	C	-1.909565	-1.125964	0.740731	C	0.955203	2.153756	-0.745527
C	-1.994200	-1.074423	-0.746215	C	-1.909565	-1.125964	-0.740731	C	0.955203	2.153757	0.745525
C	0.454373	-0.699195	-0.713424	C	0.489422	-0.628017	-0.712312	C	0.731337	-0.331105	0.715314
C	0.454373	-0.699195	0.713424	C	0.489422	-0.628017	0.712312	C	0.731337	-0.331105	-0.715315
C	-2.861611	-1.716839	1.545992	C	-2.731646	-1.828861	1.533896	C	1.527334	3.065945	-1.537608
C	-2.861611	-1.716839	-1.545992	C	-2.731646	-1.828861	-1.533896	C	1.527325	3.065952	1.537606
C	-1.432346	2.410838	1.389013	C	-1.465945	2.402227	1.366635	C	-2.468138	1.338393	-1.376956
C	-1.432346	2.410838	-1.389013	C	-1.465945	2.402227	-1.366635	C	-2.468136	1.338393	1.376962
C	1.541828	-1.256980	-1.432360	C	1.594978	-1.185785	-1.404044	C	1.250096	-1.402618	1.468738
C	1.541828	-1.256980	1.432360	C	1.594978	-1.185785	1.404044	C	1.250096	-1.402620	-1.468735
H	-1.245674	2.450015	2.461356	H	-1.284228	2.460527	2.438040	H	-2.488786	1.148759	-2.448615
H	1.440409	-1.392927	2.506589	H	1.513328	-1.329601	2.478295	H	1.365829	-1.264630	-2.541218
H	1.440409	-1.392927	-2.506589	H	1.513328	-1.329601	-2.478295	H	1.365828	-1.264622	2.541220
H	-2.786566	-1.647191	2.628783	H	-2.661341	-1.762870	2.615675	H	1.466184	2.983622	-2.619396
H	-2.786566	-1.647191	-2.628783	H	-2.661341	-1.762870	-2.615675	H	1.466176	2.983630	2.619394
H	-0.852714	-0.188702	2.393712	H	-0.830707	-0.184709	2.391802	H	0.165618	0.951551	-2.385822
H	-0.852714	-0.188702	-2.393712	H	-0.830707	-0.184709	-2.391802	H	0.165625	0.951551	2.385822
Fe	0.785625	-2.673219	0.000000	Fe	0.810036	-2.563450	0.000000	Fe	2.656869	-0.788030	0.000000
C	0.606799	-3.877829	1.327106	C	0.568350	-3.739241	1.334952	C	3.651296	0.848669	-0.000002
C	0.606799	-3.877829	-1.327106	C	0.568350	-3.739241	-1.334952	C	3.871381	-2.151148	0.000002
O	0.529271	-4.648435	-2.193354	O	0.429413	-4.483544	-2.220440	O	4.608404	-3.035346	-0.000003
O	0.529271	-4.648435	2.193354	O	0.429413	-4.483544	2.220440	O	4.338822	1.767190	-0.000001
Fe	0.148624	2.676799	0.000000	Fe	0.110521	2.620318	0.000000	Fe	-2.580026	-0.226312	0.000002
C	-0.043638	4.454420	0.000000	C	-0.030123	4.406380	0.000000	C	-4.360788	-0.182505	-0.000008
C	1.382395	2.479034	1.296626	C	1.327255	2.361493	1.293510	C	-2.271461	-1.430516	-1.296939
C	1.382395	2.479034	-1.296626	C	1.327255	2.361493	-1.293510	C	-2.271469	-1.430514	1.296946
O	2.163429	2.380719	2.145133	O	2.095558	2.197064	2.148434	O	-2.102024	-2.184765	-2.149684
O	2.163429	2.380719	-2.145133	O	2.095558	2.197064	-2.148434	O	-2.102035	-2.184764	2.149691
O	-0.205082	5.600954	0.000000	O	-0.173424	5.557572	0.000000	O	-5.509548	-0.095921	-0.000017
H	2.566077	-1.124809	-1.084501	H	2.608109	-1.002192	-1.045104	H	1.075104	-2.432795	1.162937
H	2.566077	-1.124809	1.084501	H	2.608109	-1.002192	1.045104	H	1.075105	-2.432796	-1.162930
H	-3.662831	-2.336229	-1.148486	H	-3.484771	-2.499381	-1.128254	H	2.076580	3.913665	1.136205
H	-3.662831	-2.336229	1.148486	H	-3.484771	-2.499381	1.128254	H	2.076590	3.913658	-1.136209
H	-1.245674	2.450015	-2.461356	H	-1.284228	2.460527	-2.438040	H	-2.488782	1.148759	2.448620
H	-2.265082	3.031980	1.062324	H	-2.300734	3.011573	1.021931	H	-3.137685	2.136136	-1.060133
H	-2.265082	3.031980	-1.062324	H	-2.300734	3.011573	-1.021931	H	-3.137684	2.136135	1.060139

Table S12:Optimized coordinates for the (**Hericene)Fe$_m$(CO)$_n$** structure **2-5S-10.**

	B3LYP				M06L				wB97XD		
	x	y	z		x	y	z		x	y	z
C	2.690315	0.667767	-1.210678	C	2.635686	-0.743458	1.127871	C	2.600968	0.618234	-1.242222
C	2.847127	0.880177	0.292682	C	2.825753	-0.845058	-0.373859	C	2.810496	0.883083	0.240657
C	1.846127	-1.456492	-0.227713	C	1.805014	1.433752	0.297350	C	1.787690	-1.463246	-0.160853
C	2.140024	-0.679835	-1.509718	C	2.073468	0.563611	1.512634	C	2.050327	-0.741645	-1.473825
C	1.522999	0.631834	1.009409	C	1.518586	-0.558623	-1.086343	C	1.509901	0.668061	0.997427
C	0.966997	-0.631733	0.708026	C	0.952177	0.679250	-0.702569	C	0.942522	-0.596767	0.756786
C	3.182062	-1.583963	0.521546	C	3.146065	1.616694	-0.408025	C	3.137108	-1.568213	0.551964
C	3.762821	-0.241086	0.809529	C	3.733293	0.311949	-0.784465	C	3.734759	-0.218600	0.763677
C	0.817782	1.502368	1.892161	C	0.796116	-1.377863	-1.994806	C	0.812326	1.574723	1.847937
C	-0.262870	-1.043345	1.366107	C	-0.289947	1.094815	-1.327526	C	-0.284990	-0.958625	1.442588
C	3.046024	1.605111	-2.104716	C	2.971936	-1.741169	1.957720	C	2.899075	1.524583	-2.178820
C	1.949736	-1.212538	-2.726809	C	1.846115	0.992274	2.761043	C	1.836320	-1.317565	-2.659737
C	3.714165	-2.773046	0.848942	C	3.684265	2.823675	-0.632431	C	3.670470	-2.738283	0.917630
C	4.927551	0.010308	1.429122	C	4.907144	0.120339	-1.402279	C	4.920601	0.043411	1.322315
H	3.451959	2.562782	-1.786766	H	3.383962	-2.672805	1.580503	H	3.304707	2.496041	-1.909561
H	5.270491	1.029733	1.591631	H	5.262070	-0.878560	-1.638997	H	5.274328	1.065260	1.430310
H	3.214609	-3.703892	0.589715	H	3.180273	3.732056	-0.315169	H	3.157581	-3.674302	0.713212
H	1.139865	2.541399	1.946507	H	1.116279	-2.409670	-2.123079	H	1.144501	2.611108	1.854098
H	-0.601800	-2.039136	1.063981	H	-0.655601	2.061770	-0.963970	H	-0.631186	-1.964274	1.179123
H	3.257852	1.868174	0.519952	H	3.248750	-1.811195	-0.662763	H	3.228284	1.876743	0.418499
H	1.414034	-2.438064	-0.443259	H	1.361187	2.392955	0.580109	H	1.340794	-2.446727	-0.325004
Fe	-0.493406	0.843814	0.392363	Fe	-0.452502	-0.802326	-0.427323	Fe	-0.475149	0.859186	0.397710
C	-0.236453	2.242222	-0.704441	C	-0.158476	-2.203124	0.636530	C	-0.203838	2.207771	-0.753769
C	-1.333298	-0.126180	-1.238124	C	-1.264064	0.116166	1.241657	C	-1.298048	-0.155768	-1.205390
O	-1.232815	-0.229984	-2.405002	O	-1.149433	0.183484	2.412500	O	-1.196228	-0.272337	-2.365713
O	-0.115485	3.155565	-1.402060	O	-0.021562	-3.120636	1.334044	O	-0.070150	3.086891	-1.481009
Fe	-2.333256	-0.593928	0.240121	Fe	-2.298815	0.577047	-0.226843	Fe	-2.282011	-0.601376	0.272366
C	-1.852599	1.527407	1.335700	C	-1.858998	-1.448666	-1.348498	C	-1.819108	1.607849	1.297551
C	-2.974933	-2.240270	0.141914	C	-3.022374	2.174597	-0.102040	C	-2.915032	-2.247503	0.175075
C	-3.887120	-0.033550	-0.377587	C	-3.809243	-0.027858	0.401201	C	-3.826915	-0.056493	-0.382786
O	-3.365592	-3.328377	0.022101	O	-3.487679	3.235637	0.034974	O	-3.298819	-3.330133	0.045564
O	-4.906347	0.356871	-0.768254	O	-4.812834	-0.450735	0.811484	O	-4.833315	0.319021	-0.800566
O	-2.610300	2.080268	2.023570	O	-2.630778	-1.984654	-2.040697	O	-2.582433	2.177845	1.951945
H	4.662089	-2.854862	1.377014	H	4.643276	2.939927	-1.131303	H	4.629936	-2.800173	1.424873
H	5.574943	-0.788225	1.785826	H	5.549208	0.951459	-1.683250	H	5.574544	-0.747035	1.681693
H	-0.330685	-0.903658	2.446313	H	-0.358646	1.008460	-2.414089	H	-0.335988	-0.789881	2.518405
H	0.429008	1.109911	2.831154	H	0.366528	-0.925905	-2.888275	H	0.452485	1.222088	2.813243
H	1.540771	-2.212936	-2.847649	H	1.419934	1.972845	2.951780	H	1.424439	-2.320491	-2.729285
H	2.935993	1.451098	-3.175912	H	2.828157	-1.665669	3.032313	H	2.736745	1.332967	-3.236008
H	2.179679	-0.665020	-3.638064	H	2.052059	0.369018	3.627047	H	2.044758	-0.804723	-3.594565

Table S13:Optimized coordinates for the **(Hericene)Fe$_m$(CO)$_n$** structure **2-5T-11.**

	B3LYP				M06L				wB97XD		
	x	y	z		x	y	z		x	y	z
C	2.425935	-0.197894	1.408890	C	2.199450	-0.209621	1.415758	C	2.299560	-0.183667	1.411533
C	2.730215	-0.949951	0.114631	C	2.628354	-0.979376	0.177141	C	2.664984	-0.955059	0.151025
C	1.680947	1.371349	-0.371503	C	1.631413	1.326169	-0.434670	C	1.618399	1.343184	-0.422059
C	1.823437	1.133040	1.130583	C	1.664417	1.120384	1.068413	C	1.707068	1.139501	1.082655
C	1.472474	-1.037611	-0.746757	C	1.445130	-1.086804	-0.768619	C	1.441994	-1.069868	-0.746435
C	0.899534	0.223835	-1.005012	C	0.892154	0.172378	-1.081361	C	0.869128	0.176976	-1.045295
C	3.088774	1.264256	-0.975645	C	3.069498	1.196917	-0.925598	C	3.041394	1.235493	-0.968505
C	3.691806	-0.070360	-0.697511	C	3.644305	-0.117003	-0.562940	C	3.646002	-0.085539	-0.634827
C	0.833331	-2.202227	-1.289489	C	0.801243	-2.246822	-1.306732	C	0.803158	-2.243361	-1.264521
C	-0.295127	0.318820	-1.813583	C	-0.294381	0.232803	-1.895340	C	-0.317301	0.238677	-1.864773
C	2.714593	-0.707210	2.617800	C	2.309458	-0.717229	2.651806	C	2.516141	-0.670476	2.637688
C	1.465493	2.055357	2.039123	C	1.280178	2.073678	1.929110	C	1.315038	2.072264	1.956281
C	3.658884	2.275134	-1.651959	C	3.693135	2.180747	-1.588814	C	3.627388	2.228409	-1.645295
C	4.906669	-0.494420	-1.082755	C	4.889480	-0.535672	-0.829594	C	4.876948	-0.496652	-0.955836
H	3.165857	-1.691309	2.721809	H	2.717003	-1.710061	2.819849	H	2.960046	-1.651957	2.780001
H	5.262841	-1.489804	-0.826641	H	5.229987	-1.519956	-0.521301	H	5.231494	-1.481113	-0.662421
H	3.138758	3.219287	-1.798145	H	3.191601	3.119617	-1.805456	H	3.103853	3.162133	-1.832910
H	1.174737	-3.174654	-0.935783	H	1.113861	-3.220591	-0.934011	H	1.144702	-3.204284	-0.882851
H	-0.635522	1.351635	-2.000708	H	-0.655618	1.256383	-2.109884	H	-0.657961	1.267542	-2.078305
H	3.161850	-1.935152	0.313635	H	3.039759	-1.959463	0.435609	H	3.093000	-1.932190	0.386935
H	1.231589	2.346348	-0.581075	H	1.198345	2.297052	-0.697870	H	1.164497	2.305978	-0.670151
Fe	-0.571857	-1.120355	-0.226788	Fe	-0.566551	-1.119537	-0.271922	Fe	-0.567678	-1.119151	-0.240925
C	-0.323690	-2.167947	1.207884	C	-0.346795	-2.172013	1.144558	C	-0.300004	-2.145081	1.204398
C	-1.676553	0.201616	1.253860	C	-1.465154	0.222944	1.212399	C	-1.591779	0.228413	1.233915
O	-1.771972	0.131895	2.416449	O	-1.497744	0.217435	2.384391	O	-1.664199	0.183795	2.394361
O	-0.175740	-2.882024	2.104472	O	-0.208116	-2.895672	2.041112	O	-0.128554	-2.852447	2.093465
Fe	-2.029605	0.893841	-0.440192	Fe	-1.928040	0.907464	-0.480801	Fe	-1.951726	0.897415	-0.460358
C	-1.954324	-2.042719	-0.867234	C	-2.038273	-1.931180	-0.866759	C	-1.967699	-2.039244	-0.843064
C	-1.902744	2.653871	0.155542	C	-1.698021	2.646477	0.098855	C	-1.769270	2.647773	0.132140
C	-3.880915	0.594956	-0.429826	C	-3.761102	0.650207	-0.303015	C	-3.802225	0.637651	-0.394112
O	-1.827893	3.735370	0.565637	O	-1.574975	3.728758	0.507934	O	-1.659037	3.718616	0.545771
O	-5.020204	0.399743	-0.390983	O	-4.896391	0.470299	-0.143023	O	-4.934412	0.453266	-0.303404
O	-2.773560	-2.716180	-1.335706	O	-2.938347	-2.523305	-1.304108	O	-2.802915	-2.692488	-1.294350
H	4.658862	2.195295	-2.073393	H	4.721052	2.080655	-1.928421	H	4.641623	2.144967	-2.027195
H	5.581698	0.135652	-1.658416	H	5.604540	0.090986	-1.356853	H	5.566631	0.131411	-1.513953
H	-0.365421	-0.275861	-2.723774	H	-0.370578	-0.398907	-2.779508	H	-0.378930	-0.371634	-2.763310
H	0.540484	-2.204760	-2.340209	H	0.525624	-2.251488	-2.362284	H	0.535864	-2.272705	-2.320644
H	1.043011	3.011323	1.738093	H	0.910632	3.034004	1.580957	H	0.904045	3.020001	1.619610
H	2.504169	-0.165278	3.537284	H	1.975808	-0.166018	3.527378	H	2.249081	-0.114897	3.532579
H	1.577610	1.884848	3.107620	H	1.315008	1.919667	3.004568	H	1.385967	1.919596	3.029725

Table S14:Optimized coordinates for the (**Hericene)Fe$_m$(CO)$_n$** structure **2-5T-12.**

	B3LYP				M06L				wB97XD		
	x	y	z		x	y	z		x	y	z
C	1.321438	1.275791	0.710754	C	-1.088275	1.067295	0.826208	C	1.123138	1.415500	0.707659
C	1.763513	-0.057117	1.301787	C	0.255115	1.489968	1.397075	C	1.735369	0.159136	1.299183
C	1.763519	-0.057132	-1.301785	C	0.070954	1.852621	-1.169495	C	1.735376	0.159133	-1.299183
C	1.321439	1.275782	-0.710769	C	-1.191180	1.297296	-0.565255	C	1.123138	1.415495	-0.707665
C	1.061034	-1.271004	0.706770	C	1.474633	0.966032	0.651091	C	1.197252	-1.128540	0.704241
C	1.061038	-1.271013	-0.706759	C	1.340875	1.131335	-0.773765	C	1.197258	-1.128542	-0.704241
C	3.199864	-0.202365	-0.747224	C	0.207506	3.190554	-0.424658	C	3.171841	0.200128	-0.747638
C	3.199860	-0.202359	0.747235	C	0.288647	2.984518	1.042535	C	3.171836	0.200130	0.747646
C	0.531067	-2.412205	1.385409	C	2.675886	0.525594	1.219018	C	0.800975	-2.326425	1.372937
C	0.531078	-2.412223	-1.385390	C	2.320179	0.701900	-1.666638	C	0.800986	-2.326424	-1.372945
C	0.999847	2.456231	1.431517	C	-2.285844	0.642766	1.483660	C	0.633779	2.537845	1.423908
C	0.999830	2.456212	-1.431543	C	-2.440940	1.077121	-1.204046	C	0.633752	2.537826	-1.423917
C	4.274323	-0.305564	-1.546728	C	0.241345	4.367715	-1.063976	C	4.248056	0.233896	-1.539175
C	4.274316	-0.305554	1.546744	C	0.381975	3.947227	1.969776	C	4.248047	0.233903	1.539189
H	0.856936	2.383364	2.507451	H	-2.206565	0.300151	2.513896	H	0.506168	2.438915	2.498888
H	4.173322	-0.297506	2.629637	H	0.440674	3.705786	3.026991	H	4.146431	0.229704	2.620996
H	4.173334	-0.297526	-2.629621	H	0.176002	4.422835	-2.146669	H	4.146445	0.229693	-2.620983
H	0.361868	-2.328722	2.458450	H	2.712572	0.298835	2.281283	H	0.625781	-2.262190	2.445547
H	0.361890	-2.328745	-2.458434	H	2.088567	0.689463	-2.730197	H	0.625800	-2.262184	-2.445555
H	1.750834	-0.055428	2.395017	H	0.327012	1.328084	2.475479	H	1.717694	0.158780	2.390880
H	1.750846	-0.055455	-2.395015	H	-0.003409	1.995894	-2.250856	H	1.717706	0.158773	-2.390880
Fe	-0.544677	2.088392	-0.000003	Fe	-1.871386	-0.630695	-0.121093	Fe	-0.797670	1.948176	0.000000
C	-1.732901	2.387321	-1.321907	C	-3.534123	-1.186202	-0.445339	C	-2.013596	2.101657	-1.316954
C	-1.732874	2.387271	1.321936	C	-1.540090	-1.884057	1.120927	C	-2.013561	2.101546	1.317004
O	-2.469258	2.678209	2.170626	O	-1.495470	-2.673159	1.968720	O	-2.772490	2.316097	2.159993
O	-2.469308	2.678277	-2.170570	O	-4.628191	-1.499640	-0.672066	O	-2.772541	2.316240	-2.159918
Fe	-0.895742	-1.700702	-0.000001	Fe	1.764201	-0.914183	-0.249738	Fe	-0.654832	-1.761206	-0.000009
C	-1.825515	-0.926961	1.338882	C	1.366364	-2.249912	0.935766	C	-1.642472	-1.099094	1.349907
C	-1.825477	-0.926939	-1.338899	C	-0.979282	-1.410761	-1.480821	C	-1.642457	-1.099042	-1.349912
C	-1.731702	-3.273707	-0.000014	C	3.445896	-1.596312	-0.519005	C	-1.317892	-3.409067	-0.000037
O	-2.442471	-0.546173	-2.240533	O	-0.442872	-1.905478	-2.387504	O	-2.263146	-0.800767	-2.271921
O	-2.235645	-4.316065	-0.000024	O	4.504070	-2.042023	-0.697642	O	-1.687511	-4.499618	-0.000056
O	-2.442546	-0.546216	2.240500	O	1.371844	-3.079863	1.755137	O	-2.263162	-0.800861	2.271928
H	5.282115	-0.399155	-1.147155	H	0.338851	5.307547	-0.526105	H	5.257064	0.267778	-1.136091
H	5.282109	-0.399152	1.147177	H	0.405012	5.000626	1.701230	H	5.257057	0.267785	1.136110
H	0.830946	-3.409560	-1.065264	H	3.376145	0.767174	-1.423498	H	1.233214	-3.275351	-1.058905
H	0.830936	-3.409545	1.065291	H	3.627252	0.768574	0.753531	H	1.233208	-3.275349	1.058898
H	0.856905	2.383327	-2.507474	H	-2.461840	1.069897	-2.292220	H	0.506132	2.438881	-2.498895
H	1.345266	3.431564	1.085342	H	-3.216976	1.167739	1.266229	H	0.859112	3.550239	1.086705
H	1.345246	3.431552	-1.085385	H	-3.349374	1.457362	-0.738994	H	0.859092	3.550227	-1.086738

Table S15:Optimized coordinates for the **(Hericene)Fe$_m$(CO)$_n$** structure **2-6S-1.**

		B3LYP				M06L				wB97XD	
	x	y	z		x	y	z		x	y	z
C	-2.214437	-0.734654	0.745357	C	-2.171948	-0.759368	0.739562	C	-2.193050	-0.739595	0.745272
C	-0.997491	0.019852	1.301091	C	-0.991405	0.028724	1.299566	C	-0.993289	0.029546	1.298766
C	-0.997491	0.019852	-1.301091	C	-0.991405	0.028724	-1.299566	C	-0.993289	0.029546	-1.298766
C	-2.214437	-0.734654	-0.745357	C	-2.171948	-0.759368	-0.739562	C	-2.193050	-0.739595	-0.745272
C	0.277889	-0.571864	0.707733	C	0.292308	-0.520235	0.707242	C	0.281504	-0.547813	0.705331
C	0.277889	-0.571864	-0.707733	C	0.292308	-0.520235	-0.707242	C	0.281504	-0.547813	-0.705331
C	-1.173480	1.425878	-0.709826	C	-1.197654	1.419930	-0.709519	C	-1.177174	1.426957	-0.707357
C	-1.173480	1.425878	0.709826	C	-1.197654	1.419930	0.709519	C	-1.177174	1.426957	0.707357
C	1.409101	-1.115652	1.389684	C	1.437376	-1.057615	1.365865	C	1.416043	-1.092913	1.376665
C	1.409101	-1.115652	-1.389684	C	1.437376	-1.057615	-1.365865	C	1.416043	-1.092913	-1.376665
C	-3.151996	-1.268477	1.543976	C	-3.089313	-1.332183	1.530346	C	-3.113232	-1.298362	1.536466
C	-3.151996	-1.268477	-1.543976	C	-3.089313	-1.332183	-1.530346	C	-3.113232	-1.298362	-1.536466
C	-1.357190	2.666869	-1.388403	C	-1.364768	2.672858	-1.365769	C	-1.335210	2.675338	-1.376378
C	-1.357190	2.666869	1.388403	C	-1.364768	2.672858	1.365769	C	-1.335210	2.675338	1.376378
H	-3.070841	-1.209489	2.626868	H	-3.010883	-1.281934	2.612459	H	-3.029361	-1.243237	2.618272
H	-1.169417	2.692381	2.460986	H	-1.180048	2.715956	2.437372	H	-1.146297	2.694584	2.448100
H	-1.169417	2.692381	-2.460986	H	-1.180048	2.715956	-2.437372	H	-1.146297	2.694584	-2.448100
H	1.321158	-1.285558	2.461948	H	1.372986	-1.229680	2.438720	H	1.328497	-1.258116	2.448883
H	1.321158	-1.285558	-2.461948	H	1.372986	-1.229680	-2.438720	H	1.328497	-1.258116	-2.448883
H	-0.988568	0.025446	2.394594	H	-0.983153	0.031451	2.393238	H	-0.980664	0.036472	2.390729
H	-0.988568	0.025446	-2.394594	H	-0.983153	0.031451	-2.393238	H	-0.980664	0.036472	-2.390729
Fe	0.239640	2.817180	0.000000	Fe	0.225705	2.753775	0.000000	Fe	0.232821	2.764441	0.000000
C	0.172486	4.603912	0.000000	C	0.230735	4.545079	0.000000	C	0.214365	4.547191	0.000000
C	1.455547	2.537477	1.297824	C	1.416703	2.407452	1.296273	C	1.430279	2.435749	1.297172
C	1.455547	2.537477	-1.297824	C	1.416703	2.407452	-1.296273	C	1.430279	2.435749	-1.297172
O	2.227553	2.390346	2.147660	O	2.170109	2.199757	2.155230	O	2.178220	2.245281	2.151136
O	2.227553	2.390346	-2.147660	O	2.170109	2.199757	-2.155230	O	2.178220	2.245281	-2.151136
O	0.091883	5.758843	0.000000	O	0.180985	5.703969	0.000000	O	0.145099	5.696689	0.000000
Fe	0.662256	-2.524462	0.000000	Fe	0.662521	-2.441630	0.000000	Fe	0.657784	-2.461590	0.000000
C	-0.237117	-3.391808	1.297278	C	-0.258464	-3.272604	1.297018	C	-0.251603	-3.307369	1.298110
C	-0.237117	-3.391808	-1.297278	C	-0.258464	-3.272604	-1.297018	C	-0.251603	-3.307369	-1.298110
C	2.190964	-3.449054	0.000000	C	2.175891	-3.396764	0.000000	C	2.175857	-3.393395	0.000000
O	-0.776429	-3.964452	-2.146251	O	-0.822101	-3.812616	-2.156616	O	-0.797461	-3.851438	-2.152876
O	3.200444	-4.016545	0.000000	O	3.183908	-3.971311	0.000000	O	3.186298	-3.946318	0.000000
O	-0.776429	-3.964452	2.146251	O	-0.822101	-3.812616	2.156616	O	-0.797461	-3.851438	2.152876
H	-2.141445	3.347783	-1.060802	H	-2.142371	3.352813	-1.019273	H	-2.123057	3.355499	-1.057752
H	-2.141445	3.347783	1.060802	H	-2.142371	3.352813	1.019273	H	-2.123057	3.355499	1.057752
H	2.411561	-0.834416	-1.070582	H	2.427872	-0.756347	-1.024469	H	2.413526	-0.790215	-1.062718
H	2.411561	-0.834416	1.070582	H	2.427872	-0.756347	1.024469	H	2.413526	-0.790215	1.062718
H	-3.070841	-1.209489	-2.626868	H	-3.010883	-1.281934	-2.612459	H	-3.029361	-1.243237	-2.618272
H	-4.020711	-1.788120	1.145312	H	-3.931873	-1.884592	1.122316	H	-3.968437	-1.834885	1.134127
H	-4.020711	-1.788120	-1.145312	H	-3.931873	-1.884592	-1.122316	H	-3.968437	-1.834885	-1.134127

Table S16:Optimized coordinates for the (**Hericene)Fe$_m$(CO)$_n$** structure **2-6S-2.**

	B3LYP				M06L				wB97XD		
	x	y	z		x	y	z		x	y	z
C	0.000000	0.743699	1.240315	C	0.000000	0.737836	1.156019	C	-0.000010	1.200281	-0.743437
C	0.000000	1.299280	-0.182792	C	0.000000	1.297371	-0.256348	C	-0.000001	-0.217332	-1.296877
C	0.000000	-1.299280	-0.182792	C	0.000000	-1.297371	-0.256348	C	0.000002	-0.217315	1.296881
C	0.000000	-0.743699	1.240315	C	0.000000	-0.737836	1.156019	C	0.000002	1.200291	0.743422
C	1.195036	0.709302	-0.949278	C	1.189588	0.708858	-1.011364	C	1.192409	-0.973366	-0.706848
C	1.195036	-0.709302	-0.949278	C	1.189588	-0.708858	-1.011364	C	1.192411	-0.973356	0.706863
C	-1.195036	-0.709302	-0.949278	C	-1.189588	-0.708858	-1.011364	C	-1.192405	-0.973359	0.706865
C	-1.195036	0.709302	-0.949278	C	-1.189588	0.708858	-1.011364	C	-1.192404	-0.973374	-0.706846
C	2.248496	1.390814	-1.629142	C	2.275646	1.367893	-1.657242	C	2.266576	-1.627331	-1.378817
C	2.248496	-1.390814	-1.629142	C	2.275646	-1.367893	-1.657242	C	2.266582	-1.627308	1.378841
C	0.000000	1.543402	2.318439	C	0.000000	1.530431	2.236114	C	-0.000031	2.276510	-1.535132
C	0.000000	-1.543402	2.318439	C	0.000000	-1.530431	2.236114	C	0.000027	2.276531	1.535102
C	-2.248496	-1.390814	-1.629142	C	-2.275646	-1.367893	-1.657242	C	-2.266575	-1.627317	1.378841
C	-2.248496	1.390814	-1.629142	C	-2.275646	1.367893	-1.657242	C	-2.266571	-1.627343	-1.378815
H	0.000000	2.626248	2.219164	H	0.000000	2.612592	2.142468	H	-0.000038	2.177472	-2.616895
H	-2.347404	2.463397	-1.467345	H	-2.385738	2.439650	-1.503588	H	-2.358973	-1.461824	-2.450632
H	-2.347404	-2.463397	-1.467345	H	-2.385738	-2.439650	-1.503588	H	-2.358979	-1.461776	2.450655
H	2.347404	2.463397	-1.467345	H	2.385738	2.439650	-1.503588	H	2.358983	-1.461804	-2.450633
H	2.347404	-2.463397	-1.467345	H	2.385738	-2.439650	-1.503588	H	2.358984	-1.461765	2.450654
H	0.000000	2.392839	-0.194434	H	0.000000	2.391082	-0.260921	H	-0.000001	-0.230060	-2.388905
H	0.000000	-2.392839	-0.194434	H	0.000000	-2.391082	-0.260921	H	0.000002	-0.230024	2.388910
Fe	-3.056315	0.000000	-0.251055	Fe	-2.987442	0.000000	-0.239883	Fe	-3.001977	-0.246365	0.000003
C	-3.319970	1.295914	0.972382	C	-3.163715	1.294579	0.991641	C	-3.217093	0.979266	-1.296407
C	-4.648148	0.000000	-1.062947	C	-4.627159	0.000000	-0.956365	C	-4.611255	-1.010065	0.000002
C	-3.319970	-1.295914	0.972382	C	-3.163715	-1.294579	0.991641	C	-3.217093	0.979276	1.296405
O	-5.662413	0.000000	-1.621933	O	-5.668115	0.000000	-1.469116	O	-5.626113	-1.555023	0.000005
O	-3.523894	-2.143450	1.733253	O	-3.303205	-2.150812	1.762800	O	-3.379376	1.734478	2.149315
O	-3.523894	2.143450	1.733253	O	-3.303205	2.150812	1.762800	O	-3.379382	1.734463	-2.149321
Fe	3.056315	0.000000	-0.251055	Fe	2.987442	0.000000	-0.239883	Fe	3.001980	-0.246354	0.000003
C	3.319970	1.295914	0.972382	C	3.163715	1.294579	0.991641	C	3.217083	0.979265	-1.296420
C	3.319970	-1.295914	0.972382	C	3.163715	-1.294579	0.991641	C	3.217103	0.979286	1.296401
C	4.648148	0.000000	-1.062947	C	4.627159	0.000000	-0.956365	C	4.611251	-1.010068	0.000001
O	3.523894	-2.143450	1.733253	O	3.303205	-2.150812	1.762800	O	3.379398	1.734491	2.149307
O	5.662413	0.000000	-1.621933	O	5.668115	0.000000	-1.469116	O	5.626098	-1.555046	-0.000004
O	3.523894	2.143450	1.733253	O	3.303205	2.150812	1.762800	O	3.379355	1.734447	-2.149351
H	-2.537653	-1.066585	-2.627909	H	-2.588115	-1.024654	-2.643160	H	-2.558468	-2.627579	1.063491
H	-2.537653	1.066585	-2.627909	H	-2.588115	1.024654	-2.643160	H	-2.558473	-2.627596	-1.063445
H	2.537653	-1.066585	-2.627909	H	2.588115	-1.024654	-2.643160	H	2.558480	-2.627571	1.063498
H	2.537653	1.066585	-2.627909	H	2.588115	1.024654	-2.643160	H	2.558481	-2.627585	-1.063452
H	0.000000	-2.626248	2.219164	H	0.000000	-2.612592	2.142468	H	0.000039	2.177508	2.616867
H	0.000000	1.145906	3.330991	H	0.000000	1.123967	3.244096	H	-0.000041	3.285888	-1.132848
H	0.000000	-1.145906	3.330991	H	0.000000	-1.123967	3.244096	H	0.000034	3.285903	1.132804

Table S17:Optimized coordinates for the **(Hericene)Fe$_m$(CO)$_n$** structure **2-6S-3.**

	B3LYP				M06L				wB97XD		
	x	y	z		x	y	z		x	y	z
C	1.295740	-1.167961	0.704478	C	-1.285269	1.165236	0.704642	C	1.227088	-1.122310	0.783080
C	0.000000	-1.699103	1.304053	C	0.000001	1.693098	1.302670	C	-0.089674	-1.679858	1.298484
C	0.000001	-1.699105	-1.304055	C	-0.000002	1.693098	-1.302670	C	0.089675	-1.679858	-1.298484
C	1.295741	-1.167961	-0.704480	C	-1.285271	1.165236	-0.704640	C	1.341521	-1.173135	-0.615891
C	-1.295741	-1.167963	0.704477	C	1.285270	1.165237	0.704639	C	-1.341520	-1.173136	0.615891
C	-1.295741	-1.167964	-0.704481	C	1.285268	1.165236	-0.704643	C	-1.227087	-1.122311	-0.783080
C	0.000005	-3.150018	-0.748362	C	-0.000002	3.130501	-0.742374	C	0.026983	-3.122891	-0.748186
C	-0.000002	-3.150017	0.748361	C	0.000000	3.130501	0.742374	C	-0.026980	-3.122892	0.748186
C	-2.530459	-0.888144	1.379480	C	2.524539	0.881146	1.358299	C	-2.635861	-0.942093	1.185351
C	-2.530457	-0.888146	-1.379487	C	2.524534	0.881143	-1.358308	C	-2.390363	-0.763347	-1.534800
C	2.530456	-0.888142	1.379484	C	-2.524536	0.881143	1.358306	C	2.390362	-0.763345	1.534801
C	2.530458	-0.888140	-1.379483	C	-2.524539	0.881142	-1.358301	C	2.635863	-0.942090	-1.185350
C	0.000020	-4.228272	-1.547268	C	-0.000003	4.211650	-1.532970	C	0.020736	-4.198302	-1.540202
C	-0.000014	-4.228270	1.547269	C	0.000000	4.211650	1.532969	C	-0.020731	-4.198303	1.540202
H	2.483895	-0.706201	2.453123	H	-2.501135	0.700562	2.431847	H	2.239644	-0.522197	2.586122
H	-0.000020	-4.126854	2.630154	H	0.000002	4.118290	2.614931	H	-0.063235	-4.096216	2.621120
H	0.000024	-4.126857	-2.630153	H	-0.000005	4.118289	-2.614932	H	0.063239	-4.096215	-2.621120
H	-2.483900	-0.706201	2.453118	H	2.501142	0.700566	2.431840	H	-2.689425	-0.832837	2.267468
H	-2.483897	-0.706204	-2.453125	H	2.501132	0.700562	-2.431848	H	-2.239647	-0.522200	-2.586121
H	-0.000001	-1.686526	2.397158	H	0.000003	1.684646	2.395477	H	-0.159952	-1.662174	2.387822
H	0.000002	-1.686529	-2.397160	H	-0.000004	1.684646	-2.395478	H	0.159953	-1.662173	-2.387822
Fe	2.183852	0.653388	0.000001	Fe	-2.117028	-0.644518	0.000002	Fe	2.118233	0.643142	0.030750
C	3.911437	1.068881	0.000003	C	-3.835819	-1.095712	0.000004	C	3.818888	1.108266	0.211876
C	1.679894	1.759940	-1.333623	C	-1.607881	-1.737663	-1.335136	C	1.782039	1.554506	-1.482018
C	1.679889	1.759931	1.333629	C	-1.607880	-1.737665	1.335139	C	1.423285	1.876450	1.139773
O	1.478036	2.475202	-2.219337	O	-1.424993	-2.451253	-2.231031	O	1.698678	2.084587	-2.499423
O	1.478024	2.475184	2.219350	O	-1.424992	-2.451261	2.231030	O	1.094602	2.714182	1.856372
O	5.048075	1.290673	0.000005	O	-4.973502	-1.324185	0.000012	O	4.939500	1.344056	0.334982
Fe	-2.183854	0.653385	-0.000002	Fe	2.117028	-0.644516	-0.000002	Fe	-2.118234	0.643140	-0.030749
C	-1.679895	1.759934	1.333623	C	1.607885	-1.737661	1.335138	C	-1.782037	1.554506	1.482018
C	-1.679888	1.759933	-1.333626	C	1.607878	-1.737665	-1.335137	C	-1.423290	1.876444	-1.139779
C	-3.911438	1.068881	-0.000004	C	3.835820	-1.095711	-0.000006	C	-3.818890	1.108264	-0.211870
O	-1.478022	2.475192	-2.219341	O	1.424978	-2.451261	-2.231026	O	-1.094608	2.714170	-1.856385
O	-5.048076	1.290677	-0.000003	O	4.973502	-1.324184	-0.000015	O	-4.939501	1.344056	-0.334973
O	-1.478035	2.475192	2.219340	O	1.425010	-2.451252	2.231035	O	-1.698676	2.084587	2.499423
H	0.000028	-5.240540	-1.147688	H	-0.000003	5.218810	-1.123085	H	-0.027820	-5.207310	-1.138467
H	-0.000020	-5.240539	1.147690	H	-0.000001	5.218810	1.123084	H	0.027827	-5.207310	1.138467
H	-3.428843	-1.422546	-1.069392	H	3.420408	1.403172	-1.019326	H	-3.321731	-1.295446	-1.343242
H	-3.428844	-1.422546	1.069386	H	3.420411	1.403175	1.019313	H	-3.489695	-1.471734	0.763702
H	2.483898	-0.706196	-2.453121	H	-2.501140	0.700562	-2.431841	H	2.689428	-0.832835	-2.267466
H	3.428844	-1.422541	1.069392	H	-3.420409	1.403172	1.019323	H	3.321732	-1.295441	1.343246
H	3.428844	-1.422541	-1.069390	H	-3.420411	1.403172	-1.019316	H	3.489696	-1.471732	-0.763699

Table S18: Optimized coordinates for the **(Hericene)Fe$_m$(CO)$_n$** structure **3-8S-1.**

	B3LYP				M06L				wB97XD		
	x	y	z		x	y	z		x	y	z
C	-0.470810	-1.662907	-0.719906	C	-0.482312	-1.675399	-0.722953	C	-0.474284	-1.666818	-0.720539
C	0.560903	-0.700699	-1.305002	C	0.551900	-0.728914	-1.303764	C	0.560433	-0.717868	-1.303322
C	0.518976	-0.649968	1.303551	C	0.522332	-0.721823	1.309676	C	0.522242	-0.680568	1.300708
C	-0.499147	-1.643302	0.714422	C	-0.499955	-1.684074	0.706131	C	-0.499686	-1.655167	0.706525
C	0.311009	0.690969	-0.750414	C	0.326689	0.644316	-0.719683	C	0.318445	0.664580	-0.740530
C	0.289259	0.727997	0.679720	C	0.303291	0.657200	0.706408	C	0.296495	0.693930	0.682888
C	1.848631	-1.191035	0.742928	C	1.838481	-1.249337	0.729976	C	1.845202	-1.213644	0.735652
C	1.870422	-1.216456	-0.678310	C	1.853506	-1.253136	-0.690838	C	1.864422	-1.231936	-0.680772
C	0.042100	1.832868	-1.516730	C	0.076088	1.827601	-1.433317	C	0.042291	1.814826	-1.490405
C	0.053601	1.966830	1.316617	C	0.077610	1.906861	1.330671	C	0.051984	1.938333	1.306286
C	-1.356444	-2.454765	-1.459023	C	-1.425490	-2.434072	-1.434636	C	-1.398890	-2.420952	-1.452140
C	-1.404646	-2.499222	1.378002	C	-1.444606	-2.539905	1.323755	C	-1.442772	-2.490281	1.347750
C	2.997230	-1.657601	1.445788	C	3.015996	-1.692169	1.396853	C	3.011161	-1.654201	1.424326
C	3.036910	-1.709686	-1.333357	C	3.042668	-1.697676	-1.335712	C	3.046909	-1.689370	-1.330574
H	-1.432842	-2.288924	-2.531764	H	-1.538946	-2.251955	-2.501264	H	-1.479152	-2.233528	-2.520318
H	-1.527410	-2.394981	2.454490	H	-1.581540	-2.472776	2.400713	H	-1.558509	-2.392831	2.425114
H	3.122250	-1.553840	-2.407845	H	3.135946	-1.530571	-2.406795	H	3.123820	-1.524297	-2.403522
H	3.472654	-2.588411	1.140143	H	3.505605	-2.603491	1.055102	H	3.490041	-2.583572	1.121530
H	-0.129437	1.713544	-2.584649	H	-0.103127	1.757513	-2.504343	H	-0.134361	1.697174	-2.556907
H	-0.098795	1.977087	2.394264	H	-0.080042	1.928769	2.406729	H	-0.095016	1.946221	2.384098
H	0.581375	-0.723295	-2.397598	H	0.561944	-0.728913	-2.396792	H	0.578569	-0.733451	-2.394371
H	0.499515	-0.629326	2.396324	H	0.510132	-0.715867	2.402337	H	0.502926	-0.663085	2.391806
Fe	-1.545523	1.450824	-0.042001	Fe	-1.487412	1.411621	-0.024336	Fe	-1.496335	1.415085	-0.038864
C	-2.557121	1.764218	1.395217	C	-2.503359	1.773257	1.393570	C	-2.488871	1.753277	1.404654
C	-2.148467	2.950828	-0.753194	C	-2.115168	2.877226	-0.784048	C	-2.113813	2.900036	-0.755984
O	-3.143499	2.131114	2.325579	O	-3.087231	2.175901	2.314536	O	-3.056590	2.137093	2.331838
O	-2.528353	3.936938	-1.232460	O	-2.508541	3.835336	-1.312571	O	-2.493870	3.874819	-1.242963
Fe	-2.408140	-1.170049	-0.004703	Fe	-2.361551	-1.150772	0.008699	Fe	-2.362718	-1.147658	-0.002090
C	-3.789461	-2.028245	-0.694033	C	-3.814926	-1.896056	-0.672202	C	-3.775541	-1.949918	-0.683155
C	-3.400030	-0.795538	1.430581	C	-3.274497	-0.729299	1.476393	C	-3.322553	-0.773471	1.453724
C	-2.684271	0.365869	-1.256871	C	-2.512024	0.325006	-1.334951	C	-2.598897	0.353375	-1.283858
O	-4.081727	-0.724462	2.365671	O	-3.908589	-0.638543	2.447194	O	-3.982870	-0.706748	2.396731
O	-3.200622	0.513636	-2.306837	O	-2.854152	0.421221	-2.462345	O	-3.054414	0.485549	-2.357051
O	-4.683184	-2.605613	-1.157526	O	-4.745897	-2.406028	-1.146559	O	-4.680692	-2.497131	-1.145004
Fe	3.552476	-0.173168	0.032736	Fe	3.467742	-0.165478	0.030418	Fe	3.488517	-0.171403	0.031686
C	3.600063	1.052361	-1.285989	C	3.418091	1.077627	-1.262741	C	3.472607	1.057554	-1.277973
C	3.561065	1.101448	1.306340	C	3.392062	1.075604	1.325294	C	3.439820	1.087196	1.313259
C	5.270779	-0.672245	0.067728	C	5.214983	-0.566901	0.047630	C	5.217909	-0.611106	0.059210
O	3.592995	1.902432	2.140400	O	3.352556	1.856721	2.182825	O	3.420363	1.868844	2.157282
O	6.371520	-1.028069	0.089737	O	6.332355	-0.875527	0.057970	O	6.371520	-1.028069	0.089737
O	3.655722	1.823648	-2.146706	O	3.395583	1.865107	-2.115453	O	3.655722	1.823648	-2.146706
H	0.473980	2.886368	0.914351	H	0.545586	2.804940	0.927727	H	0.473980	2.886368	0.914351
H	0.402294	2.812021	-1.214584	H	0.489899	2.774772	-1.094889	H	0.402294	2.812021	-1.214584
H	-1.606598	-3.498043	0.996009	H	-1.627495	-3.530529	0.908701	H	-1.606598	-3.498043	0.996009
H	-1.646803	-3.445106	-1.120715	H	-1.699585	-3.431931	-1.101830	H	-1.646803	-3.445106	-1.120715
H	3.054089	-1.461835	2.515546	H	3.090790	-1.520765	2.468692	H	3.054089	-1.461835	2.515546
H	3.500481	-2.630355	-0.982062	H	3.525772	-2.607948	-0.982226	H	3.500481	-2.630355	-0.982062

Table S19: Optimized coordinates for the (Hericene)$Fe_m(CO)_n$ structure 3-8S-2.

	B3LYP				M06L				wB97XD		
	x	y	z		x	y	z		x	y	z
C	0.591841	-1.190541	-0.680994	C	0.598681	-1.181580	-0.676927	C	0.476140	-1.234636	-0.676623
C	0.036135	0.069314	-1.357804	C	0.037694	0.061964	-1.355386	C	0.035255	0.059718	-1.357581
C	-0.030191	0.210740	1.236013	C	-0.036175	0.200874	1.241139	C	-0.025059	0.213462	1.230482
C	0.555227	-1.113628	0.732588	C	0.560073	-1.106180	0.736016	C	0.444393	-1.150718	0.731953
C	-1.386524	0.270594	-0.814594	C	-1.376271	0.250830	-0.813356	C	-1.358950	0.380733	-0.818338
C	-1.427290	0.330671	0.601703	C	-1.421516	0.304551	0.603130	C	-1.397743	0.446321	0.592923
C	0.783725	1.350887	0.600247	C	0.755626	1.342045	0.604683	C	0.882860	1.269833	0.593831
C	0.860588	1.248986	-0.811387	C	0.837253	1.243076	-0.805188	C	0.951459	1.159841	-0.811672
C	-2.606808	0.420984	-1.539397	C	-2.611999	0.364040	-1.514935	C	-2.569197	0.614466	-1.534079
C	-2.689759	0.508831	1.241270	C	-2.700200	0.432871	1.217799	C	-2.648788	0.716326	1.218845
C	1.113021	-2.372733	-1.287238	C	1.166282	-2.352560	-1.258384	C	0.910372	-2.457124	-1.266697
C	1.039978	-2.221800	1.487958	C	1.090623	-2.207306	1.467117	C	0.849547	-2.292672	1.480757
C	1.390262	2.490711	1.239520	C	1.326773	2.513952	1.211460	C	1.581407	2.360368	1.218265
C	1.472146	2.254742	-1.599410	C	1.412085	2.290793	-1.566324	C	1.654023	2.114248	-1.589122
H	1.313300	-2.349619	-2.357489	H	1.366743	-2.345226	-2.328050	H	1.107579	-2.453330	-2.336926
H	1.186030	-2.082723	2.558198	H	1.234326	-2.086178	2.538999	H	1.000319	-2.160796	2.550407
H	1.302572	2.240012	-2.675128	H	1.268405	2.279851	-2.645102	H	1.485995	2.107133	-2.664538
H	2.411845	2.771644	0.974405	H	2.336692	2.815011	0.920976	H	2.627093	2.534799	0.959917
H	-2.599510	0.192674	-2.604302	H	-2.618268	0.147404	-2.581296	H	-2.575618	0.385233	-2.597987
H	-2.737517	0.357478	2.318711	H	-2.763105	0.284492	2.293968	H	-2.706284	0.576851	2.296526
H	0.066254	0.010244	-2.448967	H	0.071362	0.001338	-2.446616	H	0.062684	-0.006438	-2.446822
H	-0.058376	0.273470	2.326993	H	-0.066982	0.263519	2.332234	H	-0.049944	0.281740	2.319553
Fe	0.137596	3.143446	-0.211749	Fe	0.020353	3.060897	-0.213334	Fe	0.386124	3.069712	-0.237212
C	-1.115219	3.605553	0.973575	C	-1.236441	3.416717	0.986018	C	-0.838655	3.578364	0.963275
C	0.682821	4.797450	-0.576975	C	0.456448	4.746500	-0.571888	C	1.086238	4.673577	-0.549266
O	-1.924461	3.935078	1.737515	O	-2.056890	3.666483	1.775843	O	-1.598202	3.923879	1.759277
O	1.130541	5.855900	-0.738580	O	0.846503	5.832114	-0.727645	O	1.641834	5.676423	-0.676860
Fe	-2.788945	-1.126723	-0.100388	Fe	-2.685480	-1.190420	-0.106388	Fe	-2.823929	-0.887645	-0.103056
C	-2.475386	-2.384314	-1.350644	C	-2.269167	-2.402885	-1.361875	C	-2.563794	-2.156074	-1.347996
C	-2.538246	-2.299334	1.242253	C	-2.337053	-2.336719	1.228351	C	-2.627044	-2.057583	1.244691
C	-4.577541	-1.076323	-0.144665	C	-4.477392	-1.260558	-0.148070	C	-4.601441	-0.733798	-0.150019
O	-2.414570	-3.044149	2.119417	O	-2.133277	-3.063445	2.110684	O	-2.523505	-2.785719	2.129839
O	-5.732429	-1.008500	-0.174511	O	-5.636386	-1.255192	-0.176173	O	-5.741653	-0.579057	-0.182264
O	-2.308277	-3.183876	-2.170649	O	-2.013939	-3.169524	-2.195871	O	-2.415572	-2.948055	-2.169801
Fe	2.509050	-1.589387	0.096728	Fe	2.500533	-1.477321	0.097136	Fe	2.319875	-1.754080	0.100506
C	3.358621	-0.625722	1.359036	C	3.258888	-0.444512	1.353376	C	3.207654	-0.827413	1.357421
C	3.393613	-3.139826	0.220857	C	3.492669	-2.965990	0.221323	C	3.106848	-3.350182	0.230309
C	3.438949	-0.800849	-1.228114	C	3.347962	-0.616430	-1.229264	C	3.277454	-1.017171	-1.228131
O	3.933734	-4.160282	0.301966	O	4.090158	-3.956345	0.302954	O	3.562958	-4.403857	0.315282
O	4.047248	-0.330321	-2.093066	O	3.889586	-0.081300	-2.105911	O	3.883437	-0.574230	-2.100549
O	3.911431	-0.034747	2.186543	O	3.735365	0.210920	2.185088	O	3.764199	-0.253346	2.185186
H	-3.374972	1.271966	0.875767	H	-3.399179	1.175443	0.833663	H	-3.262804	1.537690	0.853858
H	-3.304562	1.209506	-1.259906	H	-3.324184	1.130724	-1.208215	H	-3.194390	1.463822	-1.262554
H	0.741732	-3.232031	1.212010	H	0.819963	-3.219457	1.166572	H	0.458599	-3.272559	1.212436
H	0.792965	-3.347317	-0.921387	H	0.868157	-3.326994	-0.870402	H	0.500478	-3.397684	-0.902356
H	1.165900	2.651648	2.293745	H	1.111909	2.694715	2.263480	H	1.370937	2.542323	2.271254
H	2.428567	2.683141	-1.303905	H	2.355600	2.732662	-1.247648	H	2.655712	2.425961	-1.299199

Table S20: Optimized coordinates for the **(Hericene)Fe$_m$(CO)$_n$** structure **3-8T-3.**

	B3LYP				M06L				wB97XD		
	x	y	z		x	y	z		x	y	z
C	0.573012	-1.725656	-0.001360	C	-0.597892	-1.669743	-0.180963	C	-0.575331	-1.545472	-0.704637
C	-0.399330	-0.922564	0.892081	C	0.406557	-0.840963	-0.993966	C	0.453694	-0.583312	-1.297947
C	-0.728188	-0.362907	-1.628297	C	0.710374	-0.512253	1.568365	C	0.453702	-0.583313	1.297944
C	0.326081	-1.460882	-1.449013	C	-0.361994	-1.550606	1.274254	C	-0.575323	-1.545475	0.704641
C	-0.207159	0.559769	0.592037	C	0.273711	0.607561	-0.570791	C	0.357911	0.811744	-0.706079
C	-0.356118	0.854084	-0.790653	C	0.394882	0.774049	0.834650	C	0.357913	0.811743	0.706075
C	-1.970583	-1.009422	-0.995245	C	1.934669	-1.133239	0.897862	C	1.744711	-1.190662	0.707849
C	-1.785642	-1.329191	0.376039	C	1.761965	-1.332516	-0.497567	C	1.744706	-1.190660	-0.707860
C	0.148010	1.608293	1.481976	C	-0.034294	1.756844	-1.344290	C	0.327565	2.035602	-1.422432
C	-0.127368	2.182083	-1.237712	C	0.157080	2.065304	1.371867	C	0.327547	2.035606	1.422422
C	1.301258	-2.795211	0.484974	C	-1.394263	-2.643935	-0.757106	C	-1.413296	-2.485523	-1.374574
C	0.845914	-2.128229	-2.493964	C	-0.916797	-2.304805	2.237543	C	-1.413281	-2.485521	1.374591
C	-3.214340	-1.339211	-1.608682	C	3.181870	-1.515361	1.467367	C	2.874632	-1.737612	1.379128
C	-2.852374	-1.967798	1.074238	C	2.850348	-1.902117	-1.218140	C	2.874624	-1.737599	-1.379153
H	1.215077	-3.089638	1.527357	H	-1.334133	-2.834129	-1.824544	H	-1.546435	-2.358563	-2.447497
H	0.579288	-1.865236	-3.514615	H	-0.675172	-2.144506	3.283628	H	-1.546406	-2.358556	2.447515
H	-2.781556	-2.038050	2.158788	H	2.807351	-1.886539	-2.305365	H	2.951415	-1.566091	-2.451092
H	-3.654146	-2.320842	-1.439432	H	3.614582	-2.478264	1.198677	H	3.269183	-2.700493	1.058948
H	0.434448	1.344573	2.498562	H	-0.294065	1.621868	-2.392083	H	0.175703	1.992647	-2.497818
H	-0.048426	2.355137	-2.309970	H	0.049410	2.159530	2.450870	H	0.175672	1.992646	2.497807
H	-0.262219	-1.157631	1.951462	H	0.270759	-0.979790	-2.070858	H	0.452442	-0.582841	-2.389379
H	-0.881005	-0.114778	-2.681977	H	0.850992	-0.360956	2.641985	H	0.452457	-0.582841	2.389376
Fe	1.549860	1.486122	-0.124186	Fe	-1.462251	1.449880	0.176248	Fe	-1.215720	2.037396	-0.000003
C	2.457410	1.080490	-1.627135	C	-2.328902	0.827560	1.630384	C	-2.276301	2.659791	1.313149
C	2.064889	3.189450	0.020827	C	-2.047659	3.139207	0.195103	C	-2.276334	2.659666	-1.313194
O	2.983948	0.958890	-2.653030	O	-2.737056	0.619527	2.704188	O	-2.890644	3.162149	2.151514
O	2.367266	4.302562	0.103817	O	-2.382017	4.248874	0.216660	O	-2.890679	3.161983	-2.151582
Fe	2.567660	-0.907792	0.335584	Fe	-2.584876	-0.789577	-0.334546	Fe	-2.527065	-1.392235	0.000012
C	3.833998	-1.632058	-0.858643	C	-3.553565	-1.381065	-1.783026	C	-3.788346	-2.645374	0.000039
C	2.829595	1.007185	1.194641	C	-3.748578	-1.604479	0.889531	C	-3.182670	-0.401078	1.352126
C	3.399023	-1.591923	1.836373	C	-2.634705	1.069114	-1.274959	C	-3.182705	-0.401129	-1.352122
O	3.536573	1.377235	2.054329	O	-4.506267	-2.122277	1.599470	O	-3.643917	0.105439	2.276262
O	3.921988	-2.086750	2.746354	O	-3.166227	1.497029	-2.234844	O	-3.643972	0.105343	-2.276272
O	4.647459	-2.108300	-1.530790	O	-4.154156	-1.808083	-2.681408	O	-4.558709	-3.500656	0.000060
Fe	-3.536884	-0.186751	0.144451	Fe	3.466672	-0.205277	-0.148569	Fe	3.465330	-0.284377	-0.000007
C	-3.383133	0.728328	1.688175	C	3.276884	0.826423	-1.605083	C	3.557930	0.954322	-1.297587
C	-3.693973	1.327229	-0.817074	C	3.544302	1.218724	0.940967	C	3.557909	0.954306	1.297589
C	-5.250989	-0.690636	0.244321	C	5.200308	-0.643635	-0.274477	C	5.147364	-0.879090	0.000004
O	-3.819234	2.288423	-1.449506	O	3.599805	2.126027	1.663266	O	3.643697	1.720116	2.152335
O	-6.349935	-1.049074	0.298251	O	6.308888	-0.975741	-0.344598	O	6.212540	-1.316075	0.000014
O	-3.310178	1.295009	2.694359	O	3.162504	1.467914	-2.565603	O	3.643741	1.720150	-2.152314
H	-0.540628	3.021507	-0.682158	H	0.594709	2.936605	0.887005	H	0.922494	2.886045	1.086578
H	-0.338375	2.578015	1.396566	H	0.481886	2.691962	-1.129058	H	0.922487	2.886047	-1.086562
H	1.557281	-2.941115	-2.369236	H	-1.642333	-3.083164	2.013649	H	-1.396002	-3.527568	1.058143
H	1.736965	-3.535434	-0.179964	H	-1.842410	-3.432751	-0.159445	H	-1.396000	-3.527570	-1.058126
H	-3.420403	-0.926556	-2.595312	H	3.396493	-1.198142	2.485887	H	2.951436	-1.566112	2.451067
H	-3.375866	-2.802318	0.610162	H	3.364157	-2.768971	-0.804035	H	3.269184	-2.700479	-1.058984

Table S21: Optimized coordinates for the **(Hericene)Fe$_m$(CO)$_n$** structure **3-8S-4.**

		B3LYP				M06L					
	x	y	z		x	y	z		x	y	z
C	-0.656813	1.189370	0.709132	C	-0.649360	1.184514	0.709097	C	-0.633661	1.198099	0.706629
C	0.224224	0.095717	1.305428	C	0.255325	0.129556	1.309037	C	0.244202	0.113741	1.303896
C	0.224224	0.095717	-1.305428	C	0.255325	0.129556	-1.309037	C	0.244202	0.113741	-1.303896
C	-0.656813	1.189370	-0.709132	C	-0.649360	1.184514	-0.709097	C	-0.633661	1.198099	-0.706629
C	-0.225012	-1.255823	0.714464	C	-0.122085	-1.234607	0.720814	C	-0.189424	-1.237353	0.714297
C	-0.225012	-1.255823	-0.714464	C	-0.122085	-1.234607	-0.720814	C	-0.189424	-1.237353	-0.714297
C	1.638279	0.221931	-0.724400	C	1.655209	0.260489	-0.723726	C	1.651773	0.222609	-0.722289
C	1.638279	0.221931	0.724400	C	1.655209	0.260489	0.723726	C	1.651773	0.222609	0.722289
C	-0.671259	-2.436024	1.396655	C	-0.626227	-2.411282	1.377728	C	-0.682031	-2.408029	1.386397
C	-0.671259	-2.436024	-1.396655	C	-0.626227	-2.411282	-1.377728	C	-0.682031	-2.408029	-1.386397
C	-1.420166	2.184392	1.389761	C	-1.412184	2.194151	1.364889	C	-1.383669	2.209039	1.375740
C	-1.420166	2.184392	-1.389761	C	-1.412184	2.194151	-1.364889	C	-1.383669	2.209039	-1.375740
C	2.829362	0.011757	-1.425793	C	2.845462	-0.083136	-1.384723	C	2.831490	-0.086534	-1.406788
C	2.829362	0.011757	1.425793	C	2.845462	-0.083136	1.384723	C	2.831490	-0.086534	1.406788
H	-1.265222	2.298505	2.461656	H	-1.274883	2.319613	2.437120	H	-1.229917	2.317350	2.447598
H	-1.265222	2.298505	-2.461656	H	-1.274883	2.319613	-2.437120	H	-1.229917	2.317350	-2.447598
H	2.785080	-0.167661	2.497708	H	2.810878	-0.272551	2.454559	H	2.773760	-0.266104	2.477390
H	3.781564	0.383533	-1.058254	H	3.806362	0.257256	-1.008425	H	3.798432	0.254586	-1.049982
H	-0.861464	-2.354356	2.465854	H	-0.825041	-2.336933	2.445200	H	-0.871066	-2.315034	2.454007
H	-0.861464	-2.354356	-2.465854	H	-0.825041	-2.336933	-2.445200	H	-0.871066	-2.315034	-2.454007
H	0.219784	0.094316	2.398369	H	0.252340	0.129738	2.402045	H	0.239242	0.111944	2.395059
H	0.219784	0.094316	-2.398369	H	0.252340	0.129738	-2.402045	H	0.239242	0.111944	-2.395059
Fe	2.330847	-1.582408	0.000000	Fe	2.151299	-1.572693	0.000000	Fe	2.190333	-1.590551	0.000000
C	2.962690	-2.638373	1.266371	C	2.661831	-2.694537	1.260858	C	2.746353	-2.687482	1.264279
C	2.962690	-2.638373	-1.266371	C	2.661831	-2.694537	-1.260858	C	2.746353	-2.687482	-1.264279
O	3.438094	-3.279329	2.114692	O	3.041768	-3.388264	2.119508	O	3.164604	-3.358338	2.110530
O	3.438094	-3.279329	-2.114692	O	3.041768	-3.388264	-2.119508	O	3.164604	-3.358338	-2.110530
Fe	-2.132324	-1.843943	0.000000	Fe	-2.037166	-1.762104	0.000000	Fe	-2.074551	-1.767550	0.000000
C	-3.092865	-1.040066	1.298161	C	-2.944099	-0.905264	1.298230	C	-2.992131	-0.922940	1.298379
C	-3.092865	-1.040066	-1.298161	C	-2.944099	-0.905264	-1.298230	C	-2.992131	-0.922940	-1.298379
C	-2.890793	-3.461681	0.000000	C	-2.868156	-3.342121	0.000000	C	-2.885983	-3.351842	0.000000
O	-3.721749	-0.560639	-2.142698	O	-3.535249	-0.379808	-2.148090	O	-3.578834	-0.414721	-2.147310
O	-3.349963	-4.523628	0.000000	O	-3.366457	-4.389153	0.000000	O	-3.358927	-4.400924	0.000000
O	-3.721749	-0.560639	2.142698	O	-3.535249	-0.379808	2.148090	O	-3.578834	-0.414721	2.147310
Fe	-0.104253	3.093255	0.000000	Fe	-0.067159	3.043993	0.000000	Fe	-0.061097	3.053544	0.000000
C	-1.030846	4.624170	0.000000	C	-0.932122	4.614807	0.000000	C	-0.939043	4.607323	0.000000
C	1.097788	3.438269	1.296576	C	1.148791	3.305245	1.294140	C	1.151126	3.337072	1.296101
C	1.097788	3.438269	-1.296576	C	1.148791	3.305245	-1.294140	C	1.151126	3.337072	-1.296101
O	1.848353	3.675651	2.144637	O	1.919055	3.457255	2.149278	O	1.904841	3.511416	2.147701
O	1.848353	3.675651	-2.144637	O	1.919055	3.457255	-2.149278	O	1.904841	3.511416	-2.147701
O	-1.659847	5.595433	0.000000	O	-1.534130	5.605507	0.000000	O	-1.552804	5.581019	0.000000
H	-0.277145	-3.404903	-1.095861	H	-0.231314	-3.380140	-1.073529	H	-0.283754	-3.379688	-1.101677
H	-0.277145	-3.404903	1.095861	H	-0.231314	-3.380140	1.073529	H	-0.283754	-3.379688	1.101677
H	-2.438898	2.393753	-1.067675	H	-2.426393	2.395739	-1.020996	H	-2.404653	2.413187	-1.058585
H	-2.438898	2.393753	1.067675	H	-2.426393	2.395739	1.020996	H	-2.404653	2.413187	1.058585
H	2.785080	-0.167661	-2.497708	H	2.810878	-0.272551	-2.454559	H	2.773760	-0.266104	-2.477390
H	3.781564	0.383533	1.058254	H	3.806362	0.257256	1.008425	H	3.798432	0.254586	1.049982

Table S22: Optimized coordinates for the **(Hericene)Fe$_m$(CO)$_n$** structure **3-8T-5.**

	B3LYP				M06L				wB97XD		
	x	y	z		x	y	z		x	y	z
C	0.322128	-1.206959	-0.708120	C	0.589650	-1.190849	-0.660791	C	0.233691	-1.216153	-0.705690
C	0.008492	0.175916	-1.297777	C	0.010110	0.024586	-1.371602	C	0.009275	0.176621	-1.294809
C	0.008491	0.175920	1.297776	C	-0.023632	0.254198	1.217432	C	0.009268	0.176607	1.294824
C	0.322128	-1.206958	0.708124	C	0.576261	-1.064224	0.749682	C	0.233689	-1.216159	0.705691
C	-1.346163	0.591557	-0.708788	C	-1.395056	0.225331	-0.812486	C	-1.310303	0.672036	-0.706524
C	-1.346163	0.591561	0.708785	C	-1.417052	0.326767	0.601736	C	-1.310307	0.672026	0.706540
C	1.065853	1.133649	0.718678	C	0.784727	1.365461	0.545115	C	1.121407	1.057958	0.715982
C	1.065853	1.133648	-0.718682	C	0.829579	1.220689	-0.890768	C	1.121411	1.057964	-0.715953
C	-2.542950	0.955476	-1.395908	C	-2.644469	0.294309	-1.494488	C	-2.494794	1.087372	-1.382629
C	-2.542950	0.955487	1.395902	C	-2.685299	0.444447	1.238678	C	-2.494805	1.087346	1.382642
C	0.587285	-2.431778	-1.390359	C	1.155628	-2.377082	-1.211178	C	0.435017	-2.457142	-1.376572
C	0.587288	-2.431774	1.390364	C	1.129780	-2.133721	1.510814	C	0.435014	-2.457154	1.376563
C	1.942502	1.932884	1.477375	C	1.428153	2.420667	1.195800	C	2.051131	1.799587	1.467482
C	1.942507	1.932880	-1.477377	C	1.408500	2.169006	-1.727431	C	2.051142	1.799600	-1.467439
H	0.772920	-2.386147	-2.462606	H	1.336334	-2.405816	-2.283965	H	0.617859	-2.421086	-2.448812
H	0.772925	-2.386142	2.462611	H	1.292078	-1.971336	2.574565	H	0.617852	-2.421108	2.448804
H	1.781098	2.008132	-2.551204	H	1.283759	2.074412	-2.802706	H	1.894643	1.879967	-2.540493
H	2.974645	2.086227	1.164617	H	2.359753	2.821974	0.803227	H	3.093046	1.863565	1.157613
H	-2.590254	0.761391	-2.466781	H	-2.665649	0.041093	-2.552539	H	-2.548871	0.897848	-2.453101
H	-2.590255	0.761407	2.466777	H	-2.721811	0.321453	2.319306	H	-2.548890	0.897802	2.453111
H	0.008250	0.175869	-2.390935	H	0.023465	-0.075468	-2.460284	H	0.008678	0.175401	-2.386404
H	0.008249	0.175877	2.390934	H	-0.034976	0.352558	2.305950	H	0.008669	0.175374	2.386419
Fe	0.965454	3.158141	-0.000009	Fe	-0.025735	3.121504	-0.349770	Fe	1.138642	3.036677	0.000021
C	-0.859008	3.660010	-0.000025	C	-1.632767	3.460488	0.431000	C	-0.660892	3.643151	0.000048
C	1.963499	4.707436	0.000015	C	0.738057	4.777240	-0.044061	C	2.213233	4.523621	-0.000036
O	-1.940981	4.068745	-0.000031	O	-2.645554	3.725778	0.948816	O	-1.711673	4.108258	0.000064
O	2.602921	5.670546	0.000037	O	1.185690	5.830183	0.154173	O	2.914644	5.434564	-0.000094
Fe	-2.956620	-0.583025	0.000003	Fe	-2.665521	-1.217114	-0.038399	Fe	-2.932844	-0.410935	-0.000008
C	-2.900103	-1.829351	-1.297958	C	-2.260037	-2.460632	-1.266188	C	-2.901326	-1.651758	-1.298098
C	-2.900104	-1.829340	1.297975	C	-2.281828	-2.321438	1.322264	C	-2.901332	-1.651791	1.298051
C	-4.706255	-0.212263	0.000000	C	-4.456118	-1.308545	-0.046604	C	-4.664682	0.013714	-0.000006
O	-2.903169	-2.615074	2.148120	O	-2.056547	-3.022300	2.220404	O	-2.907302	-2.422255	2.153218
O	-5.830836	0.062053	-0.000001	O	-5.615343	-1.318219	-0.055662	O	-5.768114	0.342618	-0.000002
O	-2.903167	-2.615093	-2.148095	O	-2.014181	-3.249135	-2.082696	O	-2.907289	-2.422199	-2.153285
Fe	2.132595	-2.035830	0.000000	Fe	2.511576	-1.452780	0.089706	Fe	1.962552	-2.114645	-0.000001
C	3.190572	-1.374076	1.298224	C	3.314017	-0.401852	1.303093	C	3.032532	-1.486134	1.298517
C	2.669947	-3.741081	0.000002	C	3.496341	-2.942721	0.235150	C	2.428354	-3.835267	0.000010
C	3.190571	-1.374087	-1.298230	C	3.341177	-0.637016	-1.276439	C	3.032524	-1.486140	-1.298528
O	2.982340	-4.855818	0.000002	O	4.090235	-3.933910	0.330797	O	2.672758	-4.960578	0.000021
O	3.878679	-0.992637	-2.147303	O	3.878927	-0.136190	-2.175526	O	3.711824	-1.120648	-2.152816
O	3.878679	-0.992617	2.147293	O	3.834091	0.253755	2.107981	O	3.711850	-1.120647	2.152793
H	-3.095707	1.839396	1.083725	H	-3.409723	1.158263	0.850274	H	-2.994122	2.003873	1.075142
H	-3.095709	1.839386	-1.083736	H	-3.358231	1.065207	-1.202968	H	-2.994109	2.003895	-1.075112
H	0.082344	-3.342277	1.070977	H	0.855841	-3.157326	1.255426	H	-0.134314	-3.329955	1.061062
H	0.082342	-3.342280	-1.070969	H	0.870569	-3.338858	-0.784106	H	-0.134313	-3.329945	-1.061082
H	1.781089	2.008142	2.551201	H	1.274599	2.557436	2.262773	H	1.894627	1.879942	2.540536
H	2.974652	2.086212	-1.164619	H	2.272810	2.744187	-1.403605	H	3.093056	1.863568	-1.157567

Table S23: Optimized coordinates for the **(Hericene)Fe$_m$(CO)$_n$** structure **3-8T-6.**

	B3LYP				M06L				wB97XD		
	x	y	z		x	y	z		x	y	z
C	-0.700316	1.119044	0.708508	C	-0.727185	1.096408	0.708295	C	-0.692026	1.125392	0.706018
C	0.179598	0.013410	1.301838	C	0.185862	0.034496	1.303279	C	0.190666	0.032831	1.299236
C	0.179598	0.013410	-1.301838	C	0.185862	0.034496	-1.303279	C	0.190666	0.032831	-1.299236
C	-0.700316	1.119044	-0.708508	C	-0.727185	1.096408	-0.708295	C	-0.692026	1.125392	-0.706018
C	-0.321349	-1.315036	0.709664	C	-0.256057	-1.302438	0.709848	C	-0.295802	-1.292956	0.707601
C	-0.321349	-1.315036	-0.709664	C	-0.256057	-1.302438	-0.709848	C	-0.295802	-1.292956	-0.707601
C	1.587731	0.191255	-0.714432	C	1.576010	0.253709	-0.713548	C	1.591601	0.211929	-0.712119
C	1.587731	0.191255	0.714432	C	1.576010	0.253709	0.713548	C	1.591601	0.211929	0.712119
C	-0.743918	-2.495047	1.391654	C	-0.661802	-2.499592	1.367590	C	-0.722214	-2.475986	1.378102
C	-0.743918	-2.495047	-1.391654	C	-0.661802	-2.499592	-1.367590	C	-0.722214	-2.475986	-1.378102
C	-1.478581	2.101206	1.390138	C	-1.518800	2.081760	1.365628	C	-1.464630	2.117404	1.376407
C	-1.478581	2.101206	-1.390138	C	-1.518800	2.081760	-1.365628	C	-1.464630	2.117404	-1.376407
C	2.806820	0.230448	-1.432520	C	2.814113	0.266178	-1.402021	C	2.812967	0.202919	-1.422879
C	2.806820	0.230448	1.432520	C	2.814113	0.266178	1.402021	C	2.812967	0.202919	1.422879
H	-1.326288	2.216115	2.462344	H	-1.386582	2.209342	2.438246	H	-1.314042	2.227614	2.448511
H	-1.326288	2.216115	-2.462344	H	-1.386582	2.209342	-2.438246	H	-1.314042	2.227614	-2.448511
H	2.785926	0.061852	2.506580	H	2.811411	0.099878	2.475931	H	2.780443	0.035278	2.495993
H	3.642191	0.836231	-1.082203	H	3.630152	0.892463	-1.040312	H	3.658848	0.797348	-1.080459
H	-0.923783	-2.425025	2.463515	H	-0.844590	-2.455231	2.439357	H	-0.898123	-2.406424	2.449761
H	-0.923783	-2.425025	-2.463515	H	-0.844590	-2.455231	-2.439357	H	-0.898123	-2.406424	-2.449761
H	0.179347	0.013355	2.394968	H	0.185458	0.034970	2.396497	H	0.190271	0.032862	2.390706
H	0.179347	0.013355	-2.394968	H	0.185458	0.034970	-2.396497	H	0.190271	0.032862	-2.390706
Fe	2.819229	-1.380600	0.000000	Fe	2.736942	-1.322707	0.000000	Fe	2.729128	-1.378039	0.000000
C	3.246481	-2.525384	1.323991	C	3.057857	-2.485482	1.330494	C	3.056716	-2.546660	1.326897
C	3.246481	-2.525384	-1.323991	C	3.057857	-2.485482	-1.330494	C	3.056716	-2.546660	-1.326897
O	3.554114	-3.240335	2.186543	O	3.273799	-3.218131	2.210068	O	3.286699	-3.270347	2.197305
O	3.554114	-3.240335	-2.186543	O	3.273799	-3.218131	-2.210068	O	3.286699	-3.270347	-2.197305
Fe	-2.222745	-1.890105	0.000000	Fe	-2.122836	-1.871919	0.000000	Fe	-2.158340	-1.846176	0.000000
C	-3.173820	-1.081838	1.297843	C	-3.036738	-1.033316	1.296221	C	-3.076751	-1.010416	1.297538
C	-3.173820	-1.081838	-1.297843	C	-3.036738	-1.033316	-1.296221	C	-3.076751	-1.010416	-1.297538
C	-2.993794	-3.505011	0.000000	C	-2.923473	-3.477006	0.000000	C	-2.954375	-3.443604	0.000000
O	-3.792819	-0.596612	-2.146970	O	-3.617559	-0.509142	-2.154341	O	-3.655974	-0.500095	-2.151095
O	-3.459567	-4.564306	0.000000	O	-3.394448	-4.536259	0.000000	O	-3.417224	-4.497376	0.000000
O	-3.792819	-0.596612	2.146970	O	-3.617559	-0.509142	2.154341	O	-3.655974	-0.500095	2.151095
Fe	-0.182113	3.035925	0.000000	Fe	-0.204451	2.976516	0.000000	Fe	-0.165850	2.997228	0.000000
C	-1.143394	4.545231	0.000000	C	-1.128849	4.513490	0.000000	C	-1.090734	4.523374	0.000000
C	1.008653	3.413661	1.296991	C	0.997567	3.293692	1.294001	C	1.033941	3.323925	1.296303
C	1.008653	3.413661	-1.296991	C	0.997567	3.293692	-1.294001	C	1.033941	3.323925	-1.296303
O	1.749405	3.679631	2.145568	O	1.756981	3.494800	2.149021	O	1.778030	3.534860	2.148519
O	1.749405	3.679631	-2.145568	O	1.756981	3.494800	-2.149021	O	1.778030	3.534860	-2.148519
O	-1.795595	5.501304	0.000000	O	-1.770697	5.479072	0.000000	O	-1.734755	5.477554	0.000000
H	-0.364451	-3.464070	-1.070775	H	-0.255674	-3.451305	-1.023875	H	-0.324866	-3.438606	-1.060812
H	-0.364451	-3.464070	1.070775	H	-0.255674	-3.451305	1.023875	H	-0.324866	-3.438606	1.060812
H	-2.500944	2.293248	-1.068168	H	-2.538879	2.251903	-1.021122	H	-2.490329	2.297091	-1.059064
H	-2.500944	2.293248	1.068168	H	-2.538879	2.251903	1.021122	H	-2.490329	2.297091	1.059064
H	2.785926	0.061852	-2.506580	H	2.811411	0.099878	-2.475931	H	2.780443	0.035278	-2.495993
H	3.642191	0.836231	1.082203	H	3.630152	0.892463	1.040312	H	3.658848	0.797348	1.080459

Table S24: Optimized coordinates for the (Herience)$Fe_m(CO)_n$ structure 3-8S-7.

	B3LYP				M06L				wB97XD		
	x	y	z		x	y	z		x	y	z
C	2.052851	0.120553	-1.352225	C	2.003289	-0.411113	1.314588	C	2.020557	-0.292572	1.328887
C	0.535521	0.349539	-1.388613	C	0.501737	-0.662040	1.242798	C	0.512177	-0.530570	1.309019
C	1.182732	-1.891256	-0.238833	C	1.145108	1.776983	0.617029	C	1.167390	1.832590	0.456754
C	2.407334	-1.102376	-0.725641	C	2.355785	0.920503	0.974927	C	2.379246	0.994184	0.864613
C	0.027332	-0.852033	-2.197970	C	-0.071308	0.336652	2.243035	C	-0.029848	0.555437	2.236716
C	0.397526	-2.135199	-1.536904	C	0.295065	1.723555	1.884313	C	0.349193	1.910840	1.747415
C	0.390728	-0.963674	0.677964	C	0.421036	1.056006	-0.503946	C	0.403537	1.023088	-0.577560
C	0.002672	0.236348	0.038820	C	0.031693	-0.258312	-0.143814	C	0.008567	-0.240029	-0.097638
C	-0.629561	-0.710736	-3.359936	C	-0.791888	-0.043553	3.307043	C	-0.731132	0.277269	3.339159
C	0.149641	-3.370423	-1.999290	C	-0.018826	2.825263	2.578917	C	0.070216	3.071826	2.345957
C	3.092711	0.942319	-1.877617	C	3.058386	-1.307527	1.646370	C	3.063308	-1.174593	1.734028
C	3.787837	-1.452298	-0.650554	C	3.739464	1.257520	0.991532	C	3.763202	1.330835	0.827358
C	0.010878	-1.184387	2.030260	C	0.068957	1.530191	-1.793791	C	0.029526	1.393943	-1.897286
C	-0.774371	1.208222	0.777845	C	-0.728506	-1.024935	-1.096476	C	-0.755390	-1.101022	-0.973063
H	2.835826	1.955693	-2.182912	H	2.823322	-2.367201	1.722914	H	2.797980	-2.215650	1.907999
H	4.067228	-2.287460	-0.009681	H	4.038579	2.208732	0.555807	H	4.042560	2.239066	0.296673
H	-1.033289	2.130823	0.192140	H	-1.017743	-2.052180	-0.739389	H	-1.030434	-2.088134	-0.503857
H	0.120304	-0.381822	2.757752	H	0.197981	0.874858	-2.654553	H	0.173664	0.684401	-2.709942
H	-0.842922	0.271961	-3.773806	H	-0.999158	-1.090751	3.505796	H	-0.945146	-0.748760	3.624977
H	0.467528	-4.253545	-1.450074	H	0.290333	3.810852	2.243419	H	0.391801	4.016082	1.915571
H	0.270402	1.298657	-1.863032	H	0.229891	-1.693409	1.492715	H	0.244068	-1.532955	1.652021
H	1.459202	-2.828616	0.251802	H	1.418678	2.801544	0.349483	H	1.448834	2.821686	0.089244
Fe	-2.629091	1.191924	-0.267808	Fe	-2.525883	-1.192017	0.015139	Fe	-2.548476	-1.194028	0.129637
C	-3.103739	1.686956	-1.898971	C	-2.857748	-2.070199	1.498202	C	-2.974938	-1.888608	1.696654
C	-3.694419	2.431800	0.402087	C	-3.694178	-2.219805	-0.791231	C	-3.693446	-2.301369	-0.629616
O	-3.362822	1.993539	-2.988352	O	-3.042381	-2.644148	2.496571	O	-3.213175	-2.327434	2.737544
O	-4.443141	3.178860	0.879499	O	-4.518131	-2.836246	-1.336742	O	-4.483411	-2.957560	-1.154924
Fe	3.472430	0.482657	0.159977	Fe	3.393829	-0.416619	-0.224828	Fe	3.405264	-0.454900	-0.197528
C	5.200529	0.921240	0.005582	C	5.122749	-0.891687	-0.207122	C	5.124608	-0.926934	-0.124551
C	2.779841	2.054891	0.695823	C	2.654645	-1.810230	-1.077337	C	2.670411	-1.927759	-0.912621
C	3.459796	-0.176301	1.835080	C	3.344591	0.612034	-1.693683	C	3.367181	0.423078	-1.763217
O	2.355122	3.080760	1.023848	O	2.175766	-2.727169	-1.605935	O	2.204714	-2.888513	-1.343699
O	3.487970	-0.609867	2.907864	O	3.335443	1.296774	-2.631413	O	3.370276	1.008071	-2.754490
O	6.318342	1.189512	-0.126766	O	6.241765	-1.188178	-0.145437	O	6.234719	-1.214190	-0.024571
Fe	-1.704132	-0.716886	0.894066	Fe	-1.628949	0.864472	-0.767452	Fe	-1.646137	0.798140	-0.826393
C	-2.112598	-2.427896	0.531522	C	-2.030890	2.462830	-0.081263	C	-2.026035	2.470000	-0.297268
C	-2.626595	-0.630293	2.414947	C	-2.590500	1.069360	-2.254621	C	-2.601525	0.860364	-2.325068
C	-3.520227	-0.372967	-0.212847	C	-3.387445	0.354105	0.381747	C	-3.394464	0.378545	0.314586
O	-3.173363	-0.572841	3.432880	O	-3.169593	1.194703	-3.251297	O	-3.162116	0.886574	-3.329219
O	-4.486589	-1.015100	-0.417251	O	-4.301314	0.961470	0.813537	O	-4.327769	1.003658	0.652899
O	-2.406270	-3.529043	0.344405	O	-2.321432	3.515035	0.310770	O	-2.287147	3.550422	-0.009454
H	-0.386910	-3.540015	-2.930305	H	-0.611838	2.778641	3.488466	H	-0.495888	3.119424	3.272291
H	-0.979493	-1.567906	-3.931128	H	-1.198218	0.679942	4.008962	H	-1.114613	1.059108	3.989089
H	4.418507	-1.340928	-1.531340	H	4.350415	0.942092	1.836558	H	4.386543	1.120176	1.694382
H	3.888844	0.491039	-2.467738	H	3.843849	-0.973831	2.323304	H	3.849197	-0.806299	2.390735
H	0.152424	-2.180931	2.445702	H	0.212774	2.586388	-2.011646	H	0.175648	2.431878	-2.189374
H	-0.469536	1.514253	1.778350	H	-0.421072	-1.088969	-2.139947	H	-0.423919	-1.299013	-1.990657

Table S25: Optimized coordinates for the **(Hericene)Fe$_m$(CO)$_n$** structure **3-8T-8.**

	B3LYP				M06L				wB97XD		
	x	y	z		x	y	z		x	y	z
C	2.105550	-0.288389	1.296125	C	2.054772	-0.804554	1.084548	C	2.062964	-0.626035	1.174259
C	0.589815	-0.531965	1.304010	C	0.557329	-1.040575	0.921335	C	0.557227	-0.867584	1.082815
C	1.218099	1.833952	0.432090	C	1.163517	1.476964	1.108516	C	1.184112	1.641420	0.881317
C	2.449023	1.003659	0.820236	C	2.385710	0.570616	1.192824	C	2.405035	0.741888	1.066430
C	0.071348	0.562242	2.249734	C	-0.027914	-0.420996	2.186029	C	0.003423	-0.066023	2.260467
C	0.431382	1.919025	1.749206	C	0.289669	1.020134	2.276145	C	0.354543	1.376824	2.139330
C	0.409875	1.022715	-0.576300	C	0.439838	1.137512	-0.181707	C	0.414309	1.122776	-0.322320
C	0.065117	-0.255728	-0.100747	C	0.095758	-0.225283	-0.268305	C	0.066968	-0.230588	-0.205442
C	-0.580565	0.276878	3.387627	C	-0.698873	-1.144354	3.092344	C	-0.671432	-0.635119	3.262749
C	0.162057	3.089200	2.348717	C	-0.112974	1.855915	3.242715	C	0.024993	2.345169	2.998448
C	3.153050	-1.158879	1.718303	C	3.123369	-1.744777	1.121826	C	3.116704	-1.570461	1.341091
C	3.826541	1.369943	0.783492	C	3.763451	0.904190	1.329188	C	3.784445	1.093124	1.127046
C	-0.041811	1.413712	-1.881466	C	0.018591	1.997712	-1.246131	C	-0.019907	1.829850	-1.490151
C	-0.714894	-1.146733	-0.930695	C	-0.676970	-0.680612	-1.395608	C	-0.692088	-0.864542	-1.255994
H	2.904522	-2.203252	1.901958	H	2.905522	-2.776526	0.853130	H	2.864986	-2.624018	1.235155
H	4.097465	2.277658	0.245957	H	4.048536	1.949074	1.223856	H	4.052989	2.112068	0.854644
H	-0.889801	-2.142314	-0.489128	H	-0.884367	-1.767340	-1.402904	H	-0.868681	-1.941924	-1.090284
H	0.104489	0.728901	-2.717615	H	0.185544	1.672039	-2.274517	H	0.157038	1.382762	-2.468253
H	-0.787770	-0.750159	3.678909	H	-0.875654	-2.206499	2.950718	H	-0.869688	-1.703104	3.272250
H	0.475022	4.033418	1.909285	H	0.164604	2.905864	3.233141	H	0.329565	3.373756	2.826692
H	0.331920	-1.531970	1.663380	H	0.303735	-2.101868	0.830353	H	0.299623	-1.926858	1.153570
H	1.485202	2.824479	0.053678	H	1.417616	2.538520	1.177959	H	1.452125	2.695377	0.781990
Fe	-2.815011	-1.312682	-0.041366	Fe	-2.712514	-1.182626	-0.548116	Fe	-2.720349	-1.254625	-0.357151
C	-2.827862	-2.280136	1.549707	C	-2.666860	-2.645192	0.579795	C	-2.708296	-2.547897	0.975372
C	-4.536589	-1.628602	-0.719521	C	-4.476567	-1.168263	-1.142632	C	-4.456208	-1.394117	-1.042409
O	-2.855233	-2.849088	2.559085	O	-2.672707	-3.544309	1.318274	O	-2.721070	-3.319149	1.832567
O	-5.608496	-1.795879	-1.118944	O	-5.587743	-1.126989	-1.473184	O	-5.538215	-1.436937	-1.430415
Fe	3.521089	-0.458549	-0.251081	Fe	3.450458	-0.297473	-0.362861	Fe	3.452532	-0.366023	-0.332072
C	5.255589	-0.888314	-0.161906	C	5.188720	-0.719065	-0.486722	C	5.180898	-0.805849	-0.379494
C	2.845074	-1.966689	-0.961723	C	2.739264	-1.356303	-1.622393	C	2.747291	-1.616364	-1.408476
C	3.478865	0.393331	-1.836604	C	3.373947	1.148240	-1.422687	C	3.385720	0.890094	-1.613725
O	2.432717	-2.954968	-1.402328	O	2.278225	-2.063045	-2.421169	O	2.302236	-2.443092	-2.075262
O	3.486266	0.950013	-2.851382	O	3.343057	2.099028	-2.088521	O	3.366936	1.713433	-2.417830
O	6.378231	-1.154523	-0.070098	O	6.313950	-0.997563	-0.515842	O	6.297172	-1.086444	-0.354247
Fe	-1.678832	0.777743	-0.796214	Fe	-1.606543	1.003888	-0.486640	Fe	-1.630869	0.932042	-0.604767
C	-2.089593	2.473896	-0.382481	C	-1.994737	2.420446	0.515299	C	-2.015687	2.477067	0.217971
C	-2.750500	0.695442	-2.217013	C	-2.750267	1.395743	-1.798412	C	-2.712748	1.209333	-1.992108
C	-3.200537	0.290051	0.824344	C	-2.979372	-0.005005	0.896271	C	-3.093359	0.087312	0.870496
O	-3.358448	0.693159	-3.203774	O	-3.424057	1.706965	-2.692835	O	-3.325974	1.431722	-2.941632
O	-3.748692	0.984630	1.587779	O	-3.470182	0.313060	1.912671	O	-3.623147	0.566987	1.788929
O	-2.357145	3.576707	-0.163857	O	-2.240714	3.370279	1.134978	O	-2.249657	3.496746	0.692933
H	-0.387511	3.143227	3.285990	H	-0.753174	1.522102	4.055373	H	-0.567922	2.147203	3.887297
H	-0.934934	1.056725	4.058146	H	-1.103690	-0.693111	3.994590	H	-1.053537	-0.055303	4.098464
H	4.462902	1.157265	1.641205	H	4.377259	0.342675	2.032637	H	4.409338	0.669229	1.910942
H	3.947087	-0.774591	2.356785	H	3.899802	-1.634466	1.877968	H	3.894977	-1.377356	2.077066
H	0.072775	2.460506	-2.160938	H	0.134790	3.071190	-1.108207	H	0.099865	2.911958	-1.489063
H	-0.457567	-1.241268	-1.985050	H	-0.412006	-0.334535	-2.394463	H	-0.414544	-0.687832	-2.293087

Table S26: Optimized coordinates for the **(Hericene)Fe$_m$(CO)$_n$** structure **3-9S-1.**

	B3LYP				M06L				wB97XD		
	x	y	z		x	y	z		x	y	z
C	-1.132316	-0.852521	0.708253	C	-0.877160	1.098114	0.708047	C	-0.860609	1.116199	0.705908
C	0.000000	0.000000	1.302253	C	0.000000	0.000000	1.303744	C	0.000000	0.000000	1.299799
C	0.000000	0.000000	-1.302253	C	0.000000	0.000000	-1.303744	C	0.000000	0.000000	-1.299799
C	-1.132316	-0.852521	-0.708253	C	-0.877160	1.098114	-0.708047	C	-0.860609	1.116199	-0.705908
C	1.304462	-0.554354	0.708253	C	-0.512414	-1.308699	0.708047	C	-0.536353	-1.303409	0.705908
C	1.304462	-0.554354	-0.708253	C	-0.512414	-1.308699	-0.708047	C	-0.536353	-1.303409	-0.705908
C	-0.172147	1.406874	-0.708253	C	1.389574	0.210586	-0.708047	C	1.396961	0.187209	-0.705908
C	-0.172147	1.406874	0.708253	C	1.389574	0.210586	0.708047	C	1.396961	0.187209	0.705908
C	2.463524	-1.030225	1.389824	C	-0.988529	-2.479473	1.364870	C	-1.016495	-2.465332	1.375808
C	2.463524	-1.030225	-1.389824	C	-0.988529	-2.479473	-1.364870	C	-1.016495	-2.465332	-1.375808
C	-2.123963	-1.618362	1.389824	C	-1.653022	2.095828	1.364870	C	-1.626792	2.112977	1.375808
C	-2.123963	-1.618362	-1.389824	C	-1.653022	2.095828	-1.364870	C	-1.626792	2.112977	-1.375808
C	-0.339561	2.648587	-1.389824	C	2.641551	0.383645	-1.364870	C	2.643287	0.352355	-1.375808
C	-0.339561	2.648587	1.389824	C	2.641551	0.383645	1.364870	C	2.643287	0.352355	1.375808
H	-2.236742	-1.464926	2.462119	H	-1.520255	2.221141	2.437694	H	-1.476378	2.221908	2.448077
H	-2.236742	-1.464926	-2.462119	H	-1.520255	2.221141	-2.437694	H	-1.476378	2.221908	-2.448077
H	-0.150292	2.669539	2.462119	H	2.683692	0.206009	2.437694	H	2.662417	0.167627	2.448077
H	-1.121178	3.335237	-1.068265	H	3.312635	1.169979	-1.018919	H	3.316591	1.146706	-1.058231
H	2.387034	-1.204612	2.462119	H	-1.163437	-2.427150	2.437694	H	-1.186040	-2.389535	2.448077
H	2.387034	-1.204612	-2.462119	H	-1.163437	-2.427150	-2.437694	H	-1.186040	-2.389535	-2.448077
H	0.000000	0.000000	2.395420	H	0.000000	0.000000	2.397233	H	0.000000	0.000000	2.391353
H	0.000000	0.000000	-2.395420	H	0.000000	0.000000	-2.397233	H	0.000000	0.000000	-2.391353
Fe	1.251552	2.795172	0.000000	Fe	2.737475	-1.203432	0.000000	Fe	2.749304	-1.212030	0.000000
C	1.184553	4.583203	0.000000	C	4.530457	-1.186858	0.000000	C	4.533041	-1.171360	0.000000
C	2.468989	2.526373	1.298676	C	2.415463	-2.398796	1.298040	C	2.445216	-2.413916	1.298880
C	2.468989	2.526373	-1.298676	C	2.415463	-2.398796	-1.298040	C	2.445216	-2.413916	-1.298880
O	3.242398	2.391128	2.149221	O	2.227464	-3.154951	2.159213	O	2.274348	-3.164374	2.154738
O	3.242398	2.391128	-2.149221	O	2.227464	-3.154951	-2.159213	O	2.274348	-3.164374	-2.154738
O	1.105330	5.737741	0.000000	O	5.688111	-1.124047	0.000000	O	5.681168	-1.088454	0.000000
Fe	1.794914	-2.481462	0.000000	Fe	-2.410940	-1.769007	0.000000	Fe	-2.424301	-1.774952	0.000000
C	0.953409	-3.401394	1.298676	C	-3.285150	-0.892454	1.298040	C	-3.313121	-0.910661	1.298880
C	0.953409	-3.401394	-1.298676	C	-3.285150	-0.892454	-1.298040	C	-3.313121	-0.910661	-1.298880
C	3.376893	-3.317454	0.000000	C	-3.293078	-3.330062	0.000000	C	-3.280948	-3.340048	0.000000
O	0.449578	-4.003563	-2.149221	O	-3.846000	-0.351565	-2.159213	O	-3.877603	-0.387456	-2.154738
O	4.416365	-3.826114	0.000000	O	-3.817509	-4.364025	0.000000	O	-3.783213	-4.375809	0.000000
O	0.449578	-4.003563	2.149221	O	-3.846000	-0.351565	2.159213	O	-3.877603	-0.387456	2.154738
Fe	-3.046467	-0.313710	0.000000	Fe	-0.326535	2.972439	0.000000	Fe	-0.325003	2.986982	0.000000
C	-4.561446	-1.265748	0.000000	C	-1.237379	4.516920	0.000000	C	-1.252093	4.511409	0.000000
C	-3.422398	0.875021	1.298676	C	0.869687	3.291250	1.298040	C	0.867905	3.324577	1.298880
C	-3.422398	0.875021	-1.298676	C	0.869687	3.291250	-1.298040	C	0.867905	3.324577	-1.298880
O	-3.691976	1.612435	2.149221	O	1.618536	3.506516	2.159213	O	1.603254	3.551831	2.154738
O	-3.691976	1.612435	-2.149221	O	1.618536	3.506516	-2.159213	O	1.603254	3.551831	-2.154738
O	-5.521694	-1.911627	0.000000	O	-1.870602	5.488072	0.000000	O	-1.897955	5.464263	0.000000
H	3.448989	-0.696650	-1.068265	H	-0.643086	-3.453815	-1.018919	H	-0.665219	-3.445605	-1.058231
H	3.448989	-0.696650	1.068265	H	-0.643086	-3.453815	1.018919	H	-0.665219	-3.445605	1.058231
H	-2.327811	-2.638587	-1.068265	H	-2.669549	2.283837	-1.018919	H	-2.651372	2.298899	-1.058231
H	-2.327811	-2.638587	1.068265	H	-2.669549	2.283837	1.018919	H	-2.651372	2.298899	1.058231
H	-0.150292	2.669539	-2.462119	H	2.683692	0.206009	-2.437694	H	2.662417	0.167627	-2.448077
H	-1.121178	3.335237	1.068265	H	3.312635	1.169979	1.018919	H	3.316591	1.146706	1.058231

Table S27: Optimized coordinates for the **(Hericene)Fe$_m$(CO)$_n$** structure **3-9S-2.**

	B3LYP				M06L				wB97XD		
	x	y	z		x	y	z		x	y	z
C	0.442422	0.777041	0.702792	C	-0.393572	0.658778	-0.796071	C	0.392897	0.704352	0.768792
C	0.390894	-0.623377	1.302417	C	-0.377454	-0.791922	-1.245469	C	0.366121	-0.734426	1.260716
C	0.390892	-0.623383	-1.302416	C	-0.454354	-0.508214	1.343626	C	0.422221	-0.529232	-1.329731
C	0.442419	0.777038	-0.702798	C	-0.518929	0.805087	0.597852	C	0.484206	0.809756	-0.625883
C	-0.681464	-1.548838	0.704075	C	0.640252	-1.664260	-0.523258	C	-0.667039	-1.622668	0.574390
C	-0.681464	-1.548843	-0.704068	C	0.657376	-1.449671	0.870130	C	-0.679723	-1.467388	-0.821491
C	1.641488	-1.343250	-0.710054	C	-1.672541	-1.290827	0.797193	C	1.649768	-1.295810	-0.770514
C	1.641488	-1.343247	0.710058	C	-1.644591	-1.415672	-0.616565	C	1.629675	-1.385693	0.642080
C	-1.463589	-2.541969	1.380981	C	1.381645	-2.779493	-1.024299	C	-1.415961	-2.710033	1.129056
C	-1.463586	-2.541979	-1.380968	C	1.525699	-2.273405	1.646305	C	-1.521253	-2.335911	-1.582636
C	0.673485	2.020256	1.381355	C	-0.471709	1.845111	-1.587772	C	0.516764	1.903041	1.539606
C	0.673477	2.020251	-1.381367	C	-0.844601	2.107238	1.092645	C	0.793641	2.096411	-1.175363
C	2.686137	-2.030613	-1.391752	C	-2.764698	-1.888189	1.486986	C	2.724234	-1.917817	-1.466233
C	2.686137	-2.030608	1.391757	C	-2.710283	-2.122836	-1.238108	C	2.685795	-2.088180	1.286294
H	0.486143	2.048037	2.454575	H	-0.164114	1.775138	-2.630135	H	0.233477	1.843029	2.589351
H	2.785286	-1.872674	2.464782	H	-2.798783	-2.066072	-2.321088	H	2.763001	-1.993269	2.367666
H	2.785284	-1.872683	-2.464777	H	-2.898157	-1.648655	2.539972	H	2.833366	-1.691250	-2.525084
H	-1.605305	-2.422668	2.455015	H	1.483364	-2.883947	-2.102917	H	-1.516945	-2.743555	2.212607
H	-1.605301	-2.422686	-2.455003	H	1.712242	-1.977307	2.677597	H	-1.687304	-2.069562	-2.625444
H	0.389993	-0.624695	2.395344	H	-0.347841	-0.906935	-2.331948	H	0.344741	-0.816074	2.348776
H	0.389991	-0.624706	-2.395343	H	-0.480983	-0.390809	2.430132	H	0.440663	-0.446749	-2.417854
Fe	-0.869701	2.326721	-0.000003	Fe	0.891564	2.192490	-0.035111	Fe	-0.867881	2.242475	0.031198
C	-0.548906	4.075779	0.000012	C	0.696849	3.951687	-0.246380	C	-0.609961	3.986224	0.231337
C	-2.082571	2.326982	-1.336404	C	1.749680	2.179099	1.542868	C	-1.815289	2.312609	-1.495145
C	-2.082572	2.326960	1.336398	C	2.384926	2.038410	-1.025892	C	-2.290879	2.112121	1.122993
O	-2.811521	2.446072	-2.225859	O	2.178193	2.217168	2.620522	O	-2.310193	2.448989	-2.524450
O	-2.811522	2.446031	2.225855	O	3.374708	2.080481	-1.630612	O	-3.201709	2.161929	1.823860
O	-0.291985	5.204492	0.000025	O	0.506860	5.087479	-0.383043	O	-0.375645	5.105356	0.365655
Fe	-2.713948	-1.579674	0.000002	Fe	2.637616	-1.512618	0.035303	Fe	-2.650002	-1.533575	-0.030816
C	-3.519662	-0.667273	1.334236	C	3.173228	-0.917403	-1.573010	C	-3.297452	-0.898015	1.521683
C	-3.519646	-0.667259	-1.334233	C	3.545525	-0.253293	0.947708	C	-3.515280	-0.347829	-1.071462
C	-3.816886	-2.974113	-0.000011	C	3.828885	-2.819380	0.255478	C	-3.800366	-2.869185	-0.231631
O	-4.097543	-0.198586	-2.218579	O	4.256656	0.487695	1.487398	O	-4.173416	0.326194	-1.731131
O	-4.494790	-3.912505	-0.000020	O	4.554930	-3.711891	0.401929	O	-4.493979	-3.777903	-0.368309
O	-4.097572	-0.198613	2.218580	O	3.487928	-0.663753	-2.660106	O	-3.712853	-0.637836	2.561955
H	2.974154	-3.028548	-1.063166	H	-3.066574	-2.900474	1.217888	H	3.009070	-2.935431	-1.203644
H	2.974156	-3.028543	1.063175	H	-3.034233	-3.071937	-0.811526	H	2.984181	-3.064584	0.908157
H	-1.364518	-3.582988	-1.070561	H	1.487001	-3.353772	1.499147	H	-1.447455	-3.410752	-1.417811
H	-1.364522	-3.582980	1.070581	H	1.303344	-3.729249	-0.492949	H	-1.307835	-3.695485	0.675914
H	0.486131	2.048027	-2.454587	H	-0.819480	2.269109	2.168832	H	0.726012	2.207195	-2.256251
H	1.522649	2.632021	1.077145	H	-1.305417	2.528986	-1.424080	H	1.372409	2.553951	1.363428
H	1.522642	2.632019	-1.077163	H	-1.628469	2.669524	0.582096	H	1.618290	2.659485	-0.739051
Fe	3.507538	-0.654686	0.000001	Fe	-3.455461	-0.591215	-0.009044	Fe	3.450104	-0.624480	0.004612
C	5.088870	-1.491927	0.000000	C	-5.089733	-1.327495	-0.029516	C	5.052150	-1.408762	0.009557
C	3.805742	0.554138	-1.300969	C	-3.724615	0.699951	1.206897	C	3.738279	0.641911	-1.235014
C	3.805744	0.554142	1.300966	C	-3.593419	0.554931	-1.383747	C	3.649776	0.529503	1.366135
O	4.039836	1.299835	-2.154442	O	-3.935326	1.498510	2.022821	O	3.957341	1.413186	-2.060725
O	4.039837	1.299841	2.154437	O	-3.712678	1.275174	-2.286653	O	3.805806	1.237542	2.260093
O	6.093885	-2.065789	-0.000001	O	-6.123770	-1.851876	-0.041816	O	6.059980	-1.965174	0.011075

Table S28:Harmonic vibrational frequencies (in cm^{-1}) and infrared intensities (in parentheses in km/mol) for the structure **1-3S-1**

B3LYP			M06L			wB97XD		
42(0)	583(15)	1259(0)	41(0)	584(8)	1250(0)	41(0)	604(25)	1267(0)
64(0)	625(108)	1276(11)	63(0)	624(120)	1274(13)	61(0)	639(103)	1295(8)
65(0)	635(94)	1285(7)	64(0)	634(81)	1295(14)	69(0)	662(114)	1315(10)
71(0)	690(1)	1333(3)	71(0)	693(1)	1346(3)	73(0)	700(1)	1358(5)
77(0)	693(1)	1402(1)	71(0)	696(2)	1397(3)	76(0)	707(1)	1421(3)
85(0)	703(0)	1437(15)	81(0)	709(0)	1415(9)	88(0)	717(0)	1445(17)
91(0)	705(18)	1442(5)	95(0)	711(20)	1420(4)	95(0)	719(20)	1450(2)
94(0)	746(3)	1464(7)	95(0)	748(5)	1440(8)	99(0)	757(1)	1472(9)
124(1)	753(2)	1469(19)	118(1)	753(1)	1447(15)	125(1)	768(4)	1477(24)
131(0)	763(3)	1499(15)	124(0)	770(3)	1490(10)	132(0)	772(4)	1523(14)
157(0)	775(0)	1518(5)	154(0)	775(0)	1507(4)	161(0)	787(1)	1539(7)
213(0)	796(1)	1526(9)	215(0)	799(0)	1523(9)	226(0)	812(1)	1549(14)
281(1)	810(0)	1684(13)	278(2)	806(0)	1691(14)	286(1)	835(0)	1732(13)
321(0)	819(1)	1690(8)	296(1)	816(3)	1696(10)	321(0)	843(1)	1738(8)
337(0)	846(7)	1703(0)	314(1)	848(5)	1711(1)	339(1)	862(8)	1746(0)
340(0)	926(0)	1716(11)	351(0)	883(1)	1724(11)	354(1)	939(0)	1757(12)
344(1)	928(0)	2055(911)	359(1)	888(0)	2045(630)	374(1)	942(0)	2113(1008)
365(3)	933(42)	2056(648)	361(3)	891(38)	2052(896)	377(2)	947(43)	2119(693)
397(2)	937(163)	2107(890)	400(0)	895(121)	2108(794)	410(0)	951(154)	2169(883)
404(0)	940(0)	3124(13)	413(2)	924(1)	3102(51)	423(1)	953(0)	3150(18)
413(2)	944(1)	3125(0)	415(1)	936(0)	3103(0)	430(3)	966(1)	3152(15)
425(2)	954(0)	3131(14)	425(2)	942(0)	3107(41)	437(1)	968(0)	3154(1)
457(5)	960(0)	3133(10)	457(6)	948(0)	3115(21)	464(6)	978(1)	3155(15)
464(1)	966(0)	3155(1)	468(1)	966(0)	3141(39)	481(0)	988(0)	3181(2)
476(11)	983(0)	3156(1)	476(4)	976(1)	3142(15)	488(0)	995(1)	3181(13)
479(0)	984(1)	3158(9)	476(0)	984(0)	3144(4)	496(0)	1001(1)	3182(2)
486(1)	1010(0)	3159(4)	485(0)	1003(0)	3145(9)	497(8)	1024(0)	3182(4)
493(12)	1162(0)	3212(2)	491(10)	1163(0)	3200(5)	502(13)	1180(0)	3235(3)
511(0)	1187(0)	3214(5)	510(19)	1186(0)	3205(13)	526(0)	1207(0)	3238(6)
514(4)	1217(17)	3245(1)	512(1)	1220(7)	3237(12)	527(11)	1240(7)	3274(0)
526(15)	1232(4)	3246(6)	527(2)	1227(1)	3237(5)	536(9)	1243(4)	3275(8)
580(65)	1240(0)	3248(5)	580(72)	1234(0)	3239(1)	597(1)	1248(0)	3275(6)
581(9)	1242(2)	3249(3)	584(4)	1241(6)	3239(23)	601(90)	1258(6)	3275(3)

Table S29:Harmonic vibrational frequencies (in cm^{-1}) and infrared intensities (in parentheses in km/mol) for the structure **1-3T-2**

B3LYP			M06L			wB97XD		
45(0)	557(0)	1248(0)	44(0)	552(1)	1231(1)	-126(0)	514(7)	1275(0)
65(0)	578(31)	1252(3)	64(0)	557(36)	1257(15)	24(0)	528(2)	1301(15)
71(0)	579(76)	1276(15)	72(0)	580(67)	1306(10)	46(0)	548(0)	1310(4)
78(0)	612(62)	1298(1)	74(0)	587(9)	1325(3)	55(0)	698(0)	1353(0)
86(0)	624(114)	1322(2)	78(0)	592(2)	1331(2)	58(0)	701(0)	1442(32)
92(0)	632(0)	1400(0)	82(0)	625(111)	1396(0)	66(1)	702(3)	1450(1)
93(0)	635(98)	1438(7)	90(0)	634(81)	1412(3)	68(0)	718(2)	1452(0)
109(1)	682(1)	1451(0)	107(1)	685(2)	1428(0)	75(1)	722(6)	1473(2)
127(0)	696(17)	1468(8)	122(0)	701(1)	1441(4)	77(0)	741(0)	1481(27)
129(0)	716(0)	1489(9)	141(0)	705(15)	1471(9)	103(0)	750(3)	1482(28)
157(0)	727(0)	1502(11)	162(1)	735(1)	1489(9)	112(2)	764(0)	1660(48)
209(1)	756(0)	1516(6)	216(0)	754(1)	1504(3)	116(0)	773(0)	1689(2)
220(1)	769(0)	1528(9)	265(1)	768(1)	1525(12)	131(1)	786(0)	1730(11)
255(0)	798(1)	1610(7)	267(0)	801(0)	1610(45)	189(4)	825(5)	1737(6)
271(0)	809(0)	1683(18)	293(1)	806(2)	1685(47)	293(2)	837(13)	1745(1)
302(0)	823(1)	1700(6)	297(0)	816(1)	1702(13)	309(3)	909(1)	1757(9)
327(0)	827(2)	2053(897)	314(0)	843(2)	2039(588)	321(0)	920(36)	2061(1101)
337(0)	915(0)	2054(638)	348(0)	867(0)	2050(911)	340(1)	944(0)	2063(1146)
341(2)	924(109)	2105(873)	357(1)	873(90)	2105(755)	345(1)	946(1)	2136(803)
349(3)	926(1)	3124(13)	360(3)	921(0)	3102(50)	346(0)	950(47)	3152(17)
389(3)	933(1)	3126(1)	404(0)	925(2)	3104(6)	354(4)	953(0)	3154(0)
404(0)	950(0)	3130(13)	411(1)	936(0)	3105(36)	359(3)	954(156)	3182(2)
412(0)	953(0)	3133(11)	411(1)	946(1)	3115(22)	389(15)	965(1)	3183(11)
425(1)	962(2)	3156(1)	425(1)	950(1)	3141(25)	402(0)	980(0)	3184(1)
455(4)	971(3)	3159(3)	456(4)	968(2)	3145(20)	403(1)	991(0)	3184(4)
466(0)	972(0)	3170(5)	471(0)	969(0)	3145(3)	423(37)	995(0)	3193(0)
476(7)	1009(0)	3179(7)	475(2)	1001(0)	3150(16)	475(0)	1002(1)	3195(4)
485(0)	1045(17)	3211(2)	482(34)	1066(13)	3199(5)	478(1)	1203(0)	3275(3)
486(26)	1169(0)	3213(5)	485(3)	1159(1)	3205(13)	484(20)	1211(0)	3277(5)
497(2)	1196(2)	3246(3)	492(1)	1196(3)	3238(7)	491(1)	1249(5)	3277(4)
510(0)	1220(1)	3248(3)	508(15)	1202(9)	3240(13)	493(13)	1254(12)	3278(3)
512(4)	1222(3)	3281(3)	509(1)	1222(4)	3260(8)	511(18)	1258(0)	3289(3)
525(14)	1225(4)	3286(4)	523(8)	1224(0)	3260(7)	512(7)	1275(2)	3290(2)

Table S30: Harmonic vibrational frequencies (in cm^{-1}) and infrared intensities (in parentheses in km/mol) for the structure **2-6S-1**.

B3LYP			M06L			wB97XD		
36(0)	510(1)	1211(15)	36(0)	510(0)	1206(5)	37(0)	524(0)	1227(5)
42(0)	512(0)	1218(0)	45(0)	511(33)	1211(0)	46(0)	525(0)	1230(0)
47(0)	522(27)	1229(0)	51(0)	511(1)	1220(0)	52(0)	529(37)	1236(0)
66(0)	524(0)	1229(2)	64(0)	524(2)	1224(5)	68(0)	535(5)	1242(6)
71(0)	525(11)	1243(0)	66(0)	528(0)	1231(0)	73(0)	536(0)	1250(0)
74(0)	574(1)	1265(5)	72(0)	577(2)	1267(10)	75(0)	593(5)	1288(8)
76(0)	579(8)	1287(14)	73(0)	579(1)	1305(16)	77(0)	598(10)	1327(14)
79(0)	580(137)	1324(2)	74(0)	580(137)	1340(2)	81(0)	600(147)	1350(2)
86(0)	592(14)	1391(0)	83(0)	594(3)	1387(1)	88(0)	610(15)	1409(1)
88(0)	595(7)	1406(0)	84(0)	600(11)	1400(1)	92(0)	613(24)	1426(1)
91(0)	622(244)	1439(8)	90(0)	622(254)	1415(6)	95(0)	637(232)	1447(8)
93(0)	626(63)	1467(12)	91(1)	627(67)	1440(12)	97(0)	641(83)	1475(16)
96(0)	635(72)	1491(24)	95(0)	634(69)	1485(17)	99(0)	657(91)	1514(26)
123(0)	639(37)	1504(11)	119(0)	641(23)	1492(5)	125(0)	667(61)	1525(8)
125(0)	693(1)	1515(0)	120(0)	695(1)	1502(1)	127(0)	705(1)	1536(1)
154(0)	701(33)	1519(9)	150(0)	702(36)	1508(5)	157(0)	716(35)	1539(11)
161(0)	717(0)	1526(12)	160(0)	721(0)	1518(9)	165(0)	739(0)	1546(17)
184(0)	747(4)	1528(6)	188(0)	749(5)	1525(7)	195(0)	764(4)	1550(9)
248(0)	760(1)	1690(11)	253(1)	759(0)	1697(12)	266(0)	772(0)	1739(10)
275(0)	761(0)	1711(5)	273(1)	767(0)	1719(5)	279(1)	775(2)	1754(5)
327(1)	802(0)	2055(211)	298(2)	801(1)	2044(254)	330(1)	825(1)	2116(515)
338(0)	807(1)	2057(1053)	353(0)	806(0)	2046(978)	350(1)	830(0)	2117(1551)
339(0)	810(0)	2057(409)	354(0)	808(2)	2054(438)	354(0)	833(0)	2118(181)
341(0)	814(1)	2059(1423)	356(0)	810(4)	2056(1371)	358(0)	838(1)	2120(1169)
353(4)	822(0)	2107(1457)	370(2)	825(1)	2107(1272)	376(3)	848(1)	2168(1392)
380(4)	839(2)	2111(367)	383(4)	839(2)	2112(349)	397(2)	856(2)	2172(389)
392(1)	928(0)	3126(6)	405(0)	884(1)	3102(39)	407(2)	942(1)	3150(12)
405(0)	932(1)	3128(1)	408(2)	891(78)	3104(0)	419(0)	948(1)	3152(18)
406(3)	936(100)	3131(16)	415(0)	924(2)	3106(25)	428(1)	950(96)	3155(1)
415(0)	947(1)	3133(6)	425(5)	925(1)	3108(41)	428(0)	968(1)	3156(8)
424(3)	953(1)	3135(5)	428(0)	938(0)	3115(13)	435(4)	972(2)	3157(21)
429(0)	955(1)	3135(10)	428(1)	941(3)	3118(20)	439(0)	974(2)	3158(0)
456(4)	957(3)	3157(1)	456(4)	943(0)	3143(20)	463(5)	979(1)	3183(1)
463(4)	963(1)	3160(4)	465(4)	955(1)	3147(6)	474(4)	987(4)	3183(6)
465(0)	968(3)	3213(2)	469(1)	960(1)	3200(3)	482(0)	988(1)	3235(2)
466(1)	982(0)	3214(2)	470(0)	980(0)	3202(4)	485(0)	1001(0)	3237(3)
476(14)	1004(0)	3215(3)	476(5)	996(0)	3205(11)	497(9)	1017(0)	3238(5)
480(11)	1014(0)	3215(5)	480(6)	1006(0)	3208(12)	501(0)	1027(0)	3240(5)
493(0)	1147(0)	3247(2)	489(0)	1145(0)	3239(7)	503(7)	1162(0)	3277(3)
510(2)	1169(0)	3250(3)	509(9)	1169(0)	3241(9)	522(5)	1188(0)	3277(4)

Table S31: Harmonic vibrational frequencies (in cm^{-1}) and infrared intensities (in parentheses in km/mol) for the structure **2-6S-2**.

	B3LYP			M06L			wB97XD	
35(0)	509(0)	1216(2)	34(0)	508(30)	1204(0)	36(0)	525(0)	1222(2)
48(0)	511(0)	1220(0)	49(0)	509(7)	1209(0)	53(0)	526(20)	1229(0)
50(0)	513(7)	1224(0)	51(0)	510(0)	1217(1)	55(0)	526(0)	1234(0)
57(0)	518(1)	1242(2)	54(0)	512(1)	1238(1)	60(0)	530(5)	1259(1)
72(0)	523(2)	1252(0)	66(0)	527(0)	1242(0)	73(0)	538(16)	1261(0)
76(0)	526(26)	1260(14)	72(0)	528(5)	1280(21)	76(0)	548(1)	1305(5)
81(0)	578(0)	1277(6)	77(0)	578(0)	1285(4)	84(0)	599(0)	1305(11)
84(0)	579(152)	1326(5)	79(0)	579(143)	1340(6)	86(0)	600(173)	1351(10)
85(0)	611(23)	1392(0)	80(0)	613(15)	1387(1)	89(0)	628(13)	1410(0)
87(0)	620(282)	1395(0)	84(0)	620(305)	1391(0)	89(0)	636(218)	1418(0)
96(0)	622(0)	1442(9)	88(0)	627(74)	1418(7)	97(0)	641(110)	1449(10)
99(0)	625(70)	1469(13)	100(1)	629(0)	1446(10)	106(0)	651(146)	1476(14)
102(0)	629(43)	1490(32)	104(0)	629(20)	1482(19)	108(0)	655(0)	1514(34)
127(1)	652(13)	1496(0)	126(1)	652(9)	1488(3)	132(1)	690(7)	1522(1)
133(0)	693(0)	1511(0)	130(0)	694(0)	1497(0)	138(0)	702(0)	1533(0)
147(0)	693(29)	1519(13)	143(0)	697(35)	1508(10)	152(0)	708(30)	1540(17)
166(0)	706(2)	1523(0)	164(0)	706(2)	1514(4)	171(0)	719(2)	1544(2)
171(0)	749(5)	1526(17)	177(0)	747(5)	1520(11)	186(0)	770(7)	1550(22)
237(1)	759(0)	1690(7)	237(2)	761(0)	1697(7)	250(0)	771(0)	1738(6)
276(0)	768(0)	1713(3)	273(1)	775(0)	1721(2)	282(0)	778(0)	1755(2)
326(0)	787(0)	2057(550)	308(0)	780(0)	2047(0)	324(0)	812(0)	2115(836)
329(0)	809(1)	2057(0)	347(0)	806(5)	2049(1202)	350(1)	834(2)	2117(1122)
337(0)	813(0)	2059(1172)	347(0)	807(0)	2053(601)	352(0)	834(0)	2120(0)
338(0)	820(0)	2059(1241)	350(0)	817(0)	2056(1083)	361(0)	843(1)	2123(1329)
358(4)	823(1)	2104(1660)	378(3)	825(1)	2105(1491)	391(3)	848(1)	2166(1443)
380(2)	829(0)	2115(290)	385(3)	830(0)	2114(241)	406(0)	848(0)	2176(417)
388(6)	927(0)	3126(6)	404(0)	883(0)	3103(38)	415(4)	939(0)	3149(4)
404(0)	937(95)	3128(4)	404(5)	892(75)	3105(0)	422(0)	948(94)	3150(31)
412(0)	937(1)	3130(4)	414(0)	920(0)	3105(11)	427(0)	953(2)	3154(4)
423(3)	939(0)	3131(19)	425(4)	920(1)	3106(70)	438(3)	962(0)	3155(0)
429(0)	950(0)	3134(0)	428(0)	932(0)	3116(0)	441(0)	971(1)	3156(30)
442(1)	952(0)	3134(17)	454(0)	934(0)	3116(39)	463(2)	973(1)	3158(1)
455(0)	958(0)	3158(0)	457(10)	952(0)	3143(11)	464(8)	982(0)	3184(1)
456(9)	961(0)	3161(2)	467(0)	956(0)	3147(4)	482(0)	988(0)	3184(3)
463(0)	974(0)	3212(0)	468(1)	965(0)	3200(0)	482(0)	990(0)	3235(0)
464(1)	976(0)	3212(3)	469(0)	975(1)	3201(8)	482(0)	990(1)	3235(5)
476(14)	1004(0)	3214(0)	475(5)	996(0)	3206(0)	497(6)	1018(0)	3238(0)
478(12)	1007(0)	3214(9)	478(7)	999(0)	3206(24)	498(12)	1022(0)	3238(11)
492(0)	1140(0)	3247(2)	487(0)	1137(0)	3238(7)	499(0)	1156(0)	3276(2)
500(0)	1174(0)	3250(3)	499(0)	1173(0)	3241(7)	511(0)	1194(0)	3277(3)

Table S32: Harmonic vibrational frequencies (in cm^{-1}) and infrared intensities (in parentheses in km/mol) for the structure **2-6S-3**.

B3LYP			M06L			wB97XD		
35(0)	509(0)	1216(2)	-12(0)	516(4)	1165(23)	18(0)	528(14)	1193(27)
48(0)	511(0)	1220(0)	40(0)	517(0)	1200(0)	43(0)	536(0)	1221(1)
50(0)	513(7)	1224(0)	41(0)	520(34)	1212(1)	48(0)	536(15)	1225(0)
57(0)	518(1)	1242(2)	56(0)	524(2)	1232(6)	61(0)	538(2)	1242(3)
72(0)	523(2)	1252(0)	60(0)	533(5)	1234(0)	62(0)	548(3)	1252(0)
76(0)	526(26)	1260(14)	60(0)	577(0)	1245(13)	65(0)	595(80)	1261(6)
81(0)	578(0)	1277(6)	72(0)	578(115)	1327(2)	83(0)	598(3)	1333(6)
84(0)	579(152)	1326(5)	77(0)	579(19)	1332(16)	84(0)	602(79)	1354(11)
85(0)	611(23)	1392(0)	78(0)	591(0)	1379(0)	85(0)	602(1)	1397(0)
87(0)	620(282)	1395(0)	81(0)	592(6)	1411(6)	87(0)	611(19)	1436(0)
96(0)	622(0)	1442(9)	81(0)	618(110)	1411(0)	91(0)	629(193)	1440(9)
99(0)	625(70)	1469(13)	104(0)	625(90)	1440(10)	110(0)	646(10)	1470(17)
102(0)	629(43)	1490(32)	113(0)	632(151)	1494(18)	122(1)	649(45)	1525(19)
127(1)	652(13)	1496(0)	134(1)	635(2)	1498(0)	143(0)	658(169)	1529(0)
133(0)	693(0)	1511(0)	137(0)	666(7)	1501(4)	146(0)	680(8)	1530(11)
147(0)	693(29)	1519(13)	141(0)	717(0)	1504(0)	155(0)	731(32)	1534(0)
166(0)	706(2)	1523(0)	175(0)	719(36)	1523(0)	183(0)	734(0)	1549(1)
171(0)	749(5)	1526(17)	180(3)	745(5)	1531(6)	190(1)	753(5)	1560(15)
237(1)	759(0)	1690(7)	231(0)	755(7)	1695(25)	246(0)	770(7)	1738(16)
276(0)	768(0)	1713(3)	267(1)	761(0)	1711(10)	272(1)	776(0)	1750(10)
326(0)	787(0)	2057(550)	308(3)	811(0)	2033(0)	327(2)	824(0)	2106(20)
329(0)	809(1)	2057(0)	351(0)	811(1)	2050(1289)	350(0)	836(0)	2111(1710)
337(0)	813(0)	2059(1172)	354(0)	814(0)	2064(47)	351(0)	839(0)	2121(6)
338(0)	820(0)	2059(1241)	367(1)	820(3)	2073(1179)	381(0)	846(1)	2141(1278)
358(4)	823(1)	2104(1660)	370(0)	827(0)	2103(578)	381(2)	852(1)	2161(338)
380(2)	829(0)	2115(290)	377(2)	849(3)	2124(1314)	389(2)	865(4)	2184(1558)
388(6)	927(0)	3126(6)	408(0)	887(0)	3093(63)	416(1)	921(1)	3139(29)
404(0)	937(95)	3128(4)	412(0)	893(83)	3094(16)	428(0)	944(0)	3139(9)
412(0)	937(1)	3130(4)	413(1)	904(1)	3106(0)	430(0)	950(100)	3146(0)
423(3)	939(0)	3131(19)	424(0)	935(1)	3106(43)	437(0)	956(4)	3147(23)
429(0)	950(0)	3134(0)	439(3)	938(0)	3110(41)	447(2)	977(4)	3160(15)
442(1)	952(0)	3134(17)	440(1)	938(5)	3112(2)	450(3)	987(0)	3162(1)
455(0)	958(0)	3158(0)	458(5)	949(0)	3141(31)	463(7)	994(1)	3181(10)
456(9)	961(0)	3161(2)	465(0)	955(2)	3145(6)	468(1)	997(5)	3181(2)
463(0)	974(0)	3212(0)	466(3)	973(2)	3189(3)	481(0)	1002(1)	3224(3)
464(1)	976(0)	3212(3)	467(1)	998(0)	3190(4)	482(1)	1015(0)	3224(3)
476(14)	1004(0)	3214(0)	473(5)	1003(0)	3195(0)	487(8)	1023(0)	3228(0)
478(12)	1007(0)	3214(9)	483(9)	1018(0)	3195(28)	511(14)	1036(0)	3228(13)
492(0)	1140(0)	3247(2)	496(0)	1147(0)	3238(7)	511(0)	1167(0)	3275(3)
500(0)	1174(0)	3250(3)	510(0)	1154(0)	3241(11)	527(0)	1168(0)	3275(4)

Table S33: Harmonic vibrational frequencies (in cm^{-1}) and infrared intensities (in parentheses in km/mol) for the structure **2-5S-1**.

B3LYP			M06L			wB97XD		
38(0)	531(11)	1205(0)	41(0)	534(20)	1191(0)	41(0)	544(21)	1212(1)
48(0)	544(2)	1219(1)	51(0)	547(27)	1216(1)	52(0)	561(9)	1232(1)
55(0)	553(46)	1227(0)	54(0)	554(13)	1219(1)	56(0)	567(53)	1234(0)
68(0)	577(73)	1240(1)	67(0)	580(78)	1232(0)	70(0)	589(45)	1250(0)
76(0)	580(26)	1249(4)	73(0)	586(13)	1246(7)	77(0)	600(75)	1268(6)
80(0)	595(31)	1305(6)	76(0)	600(34)	1321(7)	81(0)	610(44)	1342(1)
85(0)	602(5)	1323(4)	82(0)	608(10)	1337(7)	87(0)	618(23)	1350(10)
91(0)	619(19)	1397(1)	85(0)	621(30)	1388(1)	92(0)	637(37)	1411(2)
99(0)	624(172)	1420(2)	94(0)	624(175)	1402(2)	100(0)	638(155)	1432(4)
102(1)	636(78)	1425(7)	104(1)	637(83)	1413(2)	106(1)	657(47)	1441(4)
130(0)	639(23)	1453(3)	127(0)	641(6)	1437(3)	133(0)	668(74)	1462(3)
140(2)	700(7)	1461(14)	142(2)	698(9)	1454(8)	143(1)	712(5)	1478(17)
156(0)	716(1)	1495(15)	154(0)	716(1)	1490(11)	160(0)	737(1)	1521(14)
178(1)	730(1)	1522(5)	179(0)	725(1)	1509(13)	184(1)	743(1)	1543(8)
187(7)	751(5)	1529(14)	201(9)	748(5)	1512(9)	197(10)	766(3)	1546(27)
221(5)	754(5)	1530(10)	248(5)	755(8)	1521(6)	236(7)	768(10)	1549(5)
256(4)	767(4)	1551(21)	256(5)	765(6)	1533(16)	267(2)	792(4)	1571(22)
281(1)	777(3)	1580(14)	279(1)	775(1)	1556(11)	288(0)	802(4)	1602(15)
292(4)	788(5)	1698(39)	302(8)	780(4)	1703(48)	310(7)	812(3)	1749(40)
299(4)	805(2)	2027(846)	323(2)	801(3)	2028(692)	325(1)	824(1)	2084(1058)
308(2)	807(1)	2058(695)	330(2)	807(1)	2047(676)	337(2)	830(2)	2118(1280)
339(2)	817(1)	2060(1393)	355(1)	815(3)	2056(1225)	356(0)	839(2)	2122(741)
343(1)	834(1)	2064(726)	360(2)	835(2)	2065(911)	369(2)	853(1)	2130(850)
360(1)	905(6)	2110(813)	366(2)	853(48)	2111(711)	379(2)	923(57)	2172(797)
398(10)	910(60)	3129(5)	403(0)	866(4)	3109(28)	418(1)	926(2)	3153(16)
407(0)	912(8)	3132(2)	414(1)	889(5)	3112(35)	421(7)	935(6)	3158(9)
412(1)	919(1)	3135(11)	419(0)	914(3)	3112(11)	427(1)	939(5)	3163(8)
415(2)	929(4)	3137(9)	422(7)	922(1)	3120(19)	432(3)	955(4)	3166(13)
429(1)	942(1)	3147(6)	431(5)	930(1)	3123(23)	441(1)	958(1)	3166(0)
457(6)	951(0)	3157(1)	458(7)	938(0)	3135(15)	464(11)	964(1)	3180(3)
460(6)	955(1)	3159(1)	465(6)	946(0)	3144(10)	470(4)	978(1)	3182(2)
466(0)	957(2)	3166(1)	470(0)	951(1)	3147(9)	484(0)	980(1)	3186(3)
470(1)	981(0)	3216(2)	473(1)	977(0)	3205(4)	486(6)	998(0)	3239(3)
478(12)	987(0)	3219(4)	479(10)	983(0)	3211(11)	492(5)	1002(0)	3242(5)
486(5)	1012(0)	3231(2)	487(5)	1005(0)	3215(7)	502(7)	1024(0)	3252(3)
494(6)	1155(0)	3250(3)	494(2)	1151(1)	3235(5)	513(6)	1173(0)	3275(1)
511(1)	1160(1)	3252(0)	510(20)	1158(1)	3240(5)	523(1)	1176(1)	3279(2)
520(17)	1187(7)	3256(1)	510(4)	1182(4)	3245(9)	529(21)	1206(4)	3279(3)

Table S34: Harmonic vibrational frequencies (in cm^{-1}) and infrared intensities (in parentheses in km/mol) for the structure **2-5S-2**.

B3LYP			M06L			wB97XD		
40(0)	522(18)	1211(1)	39(0)	519(2)	1203(1)	41(0)	534(4)	1222(0)
47(0)	540(6)	1216(0)	47(0)	541(8)	1206(1)	49(0)	551(13)	1223(2)
57(0)	551(48)	1224(0)	59(0)	546(52)	1220(1)	63(0)	563(63)	1236(0)
72(0)	568(23)	1243(0)	68(0)	575(25)	1234(1)	74(0)	583(33)	1253(0)
75(0)	580(62)	1261(2)	71(0)	580(51)	1260(1)	75(0)	600(67)	1281(1)
82(0)	590(52)	1282(9)	79(0)	599(59)	1297(14)	85(0)	607(72)	1320(7)
86(0)	606(13)	1325(8)	81(0)	609(16)	1338(9)	87(0)	620(23)	1353(12)
91(0)	623(185)	1397(1)	88(0)	622(195)	1387(0)	95(0)	638(153)	1410(1)
100(0)	628(34)	1410(3)	95(0)	629(52)	1399(4)	102(0)	647(91)	1428(4)
103(1)	636(47)	1428(7)	104(1)	640(16)	1412(3)	109(0)	663(42)	1441(6)
136(0)	652(5)	1451(5)	132(0)	656(4)	1437(3)	139(0)	675(10)	1463(2)
149(0)	694(7)	1462(11)	153(0)	692(10)	1447(9)	159(0)	709(8)	1473(15)
167(4)	715(2)	1500(15)	171(1)	714(2)	1488(10)	176(1)	732(3)	1525(14)
178(4)	729(2)	1519(3)	188(5)	726(1)	1506(3)	191(7)	749(2)	1540(4)
185(0)	747(2)	1526(4)	192(1)	744(3)	1511(14)	198(1)	763(4)	1543(17)
206(6)	761(4)	1529(20)	232(17)	761(8)	1521(10)	228(11)	773(2)	1550(17)
251(8)	767(4)	1551(28)	262(3)	766(2)	1533(23)	272(5)	789(6)	1569(30)
279(2)	773(4)	1582(13)	278(2)	772(1)	1561(10)	286(2)	798(5)	1605(14)
291(2)	787(4)	1700(28)	293(3)	786(3)	1703(32)	304(2)	812(2)	1748(30)
293(1)	802(0)	2027(917)	321(1)	799(2)	2029(736)	323(2)	822(1)	2084(1143)
307(3)	810(1)	2057(836)	335(3)	806(2)	2046(740)	335(3)	834(1)	2116(1084)
340(0)	820(1)	2060(794)	346(1)	814(2)	2055(933)	356(1)	844(1)	2122(652)
346(3)	829(0)	2065(1109)	366(3)	828(1)	2065(1087)	375(5)	846(1)	2130(1020)
359(5)	903(6)	2110(777)	370(7)	850(51)	2110(670)	376(4)	925(60)	2171(770)
389(3)	909(44)	3129(4)	402(2)	864(7)	3108(39)	414(4)	926(6)	3152(17)
413(1)	910(27)	3131(10)	406(1)	884(4)	3110(24)	421(1)	934(9)	3157(10)
415(1)	923(1)	3134(4)	416(1)	912(1)	3115(13)	432(1)	944(1)	3161(7)
427(2)	927(1)	3135(11)	428(2)	921(0)	3119(19)	440(2)	949(1)	3164(16)
437(2)	939(1)	3145(9)	448(2)	936(0)	3122(34)	457(4)	956(2)	3167(3)
453(6)	952(0)	3159(1)	455(9)	936(0)	3137(11)	463(6)	969(0)	3181(2)
457(5)	954(1)	3160(1)	464(6)	943(1)	3145(14)	466(5)	979(1)	3183(0)
465(0)	964(1)	3166(2)	468(0)	955(0)	3147(4)	484(0)	982(2)	3186(4)
470(0)	979(0)	3213(2)	475(1)	974(0)	3202(5)	487(4)	993(0)	3236(3)
479(10)	982(0)	3216(4)	481(9)	980(0)	3209(11)	495(7)	1000(0)	3240(5)
491(7)	1014(0)	3229(3)	490(5)	1006(0)	3214(8)	508(12)	1028(0)	3249(4)
494(8)	1148(0)	3251(2)	498(11)	1144(0)	3237(4)	514(5)	1163(1)	3277(1)
508(2)	1165(1)	3253(0)	509(1)	1161(1)	3242(6)	523(0)	1182(1)	3279(2)
512(1)	1194(1)	3256(1)	511(18)	1184(0)	3244(7)	529(17)	1205(1)	3279(2)

Table S35: Harmonic vibrational frequencies (in cm^{-1}) and infrared intensities (in parentheses in km/mol) for the structure **2-5S-3**.

B3LYP			M06L			wB97XD		
28(1)	529(23)	1225(1)	18(1)	532(2)	1218(2)	25(1)	541(12)	1239(4)
55(0)	532(5)	1230(2)	50(0)	536(9)	1225(0)	50(0)	549(10)	1239(0)
58(0)	553(82)	1237(1)	57(0)	556(139)	1226(0)	60(0)	570(127)	1242(0)
64(0)	560(74)	1252(0)	58(0)	557(71)	1238(0)	60(1)	576(95)	1258(0)
73(0)	571(40)	1267(6)	73(0)	573(47)	1265(15)	76(0)	592(47)	1287(10)
83(1)	584(17)	1319(12)	81(1)	586(19)	1336(13)	86(1)	599(3)	1359(13)
93(0)	585(6)	1343(8)	86(0)	589(22)	1354(5)	93(0)	603(30)	1368(11)
97(0)	593(5)	1415(4)	91(0)	594(1)	1400(4)	99(0)	606(4)	1427(5)
105(0)	607(4)	1429(0)	100(0)	617(4)	1413(0)	109(0)	625(10)	1443(0)
137(0)	625(72)	1438(8)	130(0)	621(68)	1415(5)	139(0)	643(60)	1448(8)
152(3)	631(110)	1464(9)	153(2)	627(92)	1442(12)	159(0)	654(114)	1478(16)
153(0)	701(7)	1479(1)	169(0)	697(4)	1480(2)	159(4)	716(5)	1500(0)
176(1)	703(4)	1479(29)	184(1)	706(11)	1484(10)	187(1)	718(10)	1506(25)
187(0)	714(0)	1536(29)	194(1)	718(0)	1519(17)	199(0)	733(0)	1552(35)
190(0)	747(3)	1539(7)	201(0)	747(5)	1520(8)	199(1)	762(4)	1554(11)
239(19)	755(0)	1554(17)	245(10)	759(0)	1539(6)	247(31)	767(1)	1574(11)
249(1)	760(0)	1555(10)	256(1)	760(0)	1539(11)	267(0)	776(0)	1576(13)
271(3)	789(5)	1687(12)	270(3)	793(3)	1691(19)	277(4)	812(4)	1736(11)
292(0)	794(1)	1709(7)	311(5)	795(0)	1715(8)	330(0)	826(0)	1753(8)
298(7)	807(1)	1896(434)	324(0)	809(1)	1903(379)	332(4)	830(4)	1945(473)
324(2)	809(2)	2025(23)	330(7)	818(0)	2023(3)	343(3)	838(1)	2086(95)
330(34)	822(1)	2051(956)	355(0)	826(1)	2053(957)	356(1)	847(2)	2107(1078)
332(6)	843(0)	2058(1144)	361(1)	846(1)	2064(1084)	359(1)	862(1)	2112(1058)
348(19)	929(0)	2091(1660)	375(15)	892(5)	2095(1548)	388(66)	945(0)	2150(1772)
352(4)	933(8)	3128(8)	378(4)	899(69)	3109(31)	390(12)	948(5)	3158(20)
380(1)	941(88)	3130(1)	391(1)	926(1)	3112(12)	396(1)	955(89)	3158(1)
391(126)	943(3)	3141(10)	397(7)	929(1)	3114(56)	400(110)	968(3)	3159(18)
395(2)	947(2)	3142(5)	407(43)	932(0)	3114(10)	405(20)	974(0)	3161(1)
415(4)	952(8)	3154(2)	412(0)	939(4)	3128(7)	429(1)	977(0)	3172(3)
415(0)	957(3)	3154(2)	416(44)	941(4)	3128(21)	430(15)	984(7)	3172(7)
443(18)	960(1)	3156(1)	447(35)	950(1)	3141(32)	452(8)	991(4)	3181(6)
454(4)	968(0)	3159(7)	459(4)	965(1)	3146(9)	464(7)	996(1)	3183(7)
466(4)	980(0)	3225(1)	461(2)	979(0)	3207(5)	481(2)	998(1)	3243(3)
479(6)	997(1)	3225(3)	478(16)	993(0)	3208(8)	487(4)	1013(1)	3243(4)
490(47)	1008(0)	3240(1)	485(54)	1005(0)	3222(3)	508(82)	1022(0)	3261(1)
491(0)	1170(0)	3241(1)	502(0)	1165(0)	3222(7)	508(1)	1186(0)	3261(2)
517(2)	1171(0)	3246(3)	518(7)	1167(0)	3238(9)	530(0)	1188(0)	3275(4)
525(14)	1200(18)	3249(4)	530(12)	1199(10)	3242(10)	539(3)	1222(14)	3277(4)

Table S36: Harmonic vibrational frequencies (in cm^{-1}) and infrared intensities (in parentheses in km/mol) for the structure **2-5S-4**.

B3LYP			M06L			wB97XD		
39(0)	522(6)	1219(1)	37(1)	521(11)	1214(0)	40(0)	533(27)	1232(0)
40(0)	524(18)	1225(2)	39(0)	528(3)	1218(0)	41(0)	533(2)	1235(1)
49(0)	536(31)	1229(1)	49(0)	536(29)	1226(5)	52(0)	548(32)	1244(6)
65(0)	566(14)	1244(0)	64(0)	574(12)	1234(0)	67(0)	570(23)	1252(0)
71(0)	566(22)	1259(3)	67(0)	579(33)	1268(7)	74(0)	585(18)	1287(6)
76(0)	579(77)	1278(12)	74(0)	580(58)	1294(12)	75(0)	600(88)	1316(12)
79(0)	597(0)	1321(1)	75(0)	597(2)	1335(0)	80(0)	614(48)	1347(0)
84(0)	602(41)	1387(0)	79(0)	609(55)	1384(1)	85(0)	619(51)	1405(0)
88(0)	621(34)	1402(0)	87(1)	624(46)	1396(1)	93(0)	639(99)	1423(1)
91(0)	624(105)	1440(9)	92(0)	625(75)	1419(6)	96(0)	644(11)	1449(9)
93(0)	636(104)	1467(12)	97(0)	638(105)	1449(11)	98(0)	671(114)	1480(15)
112(0)	691(1)	1482(22)	106(0)	693(2)	1474(16)	114(0)	704(2)	1504(21)
123(1)	698(25)	1500(12)	120(1)	701(20)	1486(6)	125(1)	712(26)	1524(8)
148(0)	708(4)	1513(2)	148(0)	706(13)	1495(1)	153(0)	728(5)	1529(4)
158(0)	730(0)	1516(6)	156(0)	730(0)	1503(5)	162(0)	753(0)	1537(7)
182(0)	749(5)	1520(19)	184(0)	751(6)	1517(16)	192(0)	765(5)	1543(28)
237(0)	764(1)	1525(8)	245(0)	767(3)	1522(5)	256(1)	774(1)	1550(9)
275(1)	766(5)	1690(11)	273(1)	770(1)	1697(13)	280(1)	785(8)	1739(11)
294(1)	798(7)	1711(5)	304(1)	792(0)	1720(5)	306(1)	821(8)	1754(5)
328(1)	803(1)	2029(1034)	325(2)	799(7)	2019(931)	343(0)	827(1)	2091(1234)
337(0)	807(0)	2057(672)	342(0)	801(0)	2047(628)	353(0)	831(0)	2117(863)
340(1)	822(0)	2058(754)	355(1)	820(0)	2055(763)	361(0)	845(1)	2122(689)
351(2)	836(2)	2075(1385)	370(1)	838(2)	2069(1333)	380(1)	853(2)	2138(1313)
382(2)	899(5)	2110(784)	387(2)	885(3)	2110(730)	401(1)	927(3)	2171(787)
398(2)	928(1)	3109(17)	403(0)	887(2)	3086(45)	409(1)	942(1)	3133(21)
401(0)	935(1)	3126(8)	412(0)	894(77)	3104(31)	420(0)	950(92)	3148(13)
412(1)	936(100)	3128(3)	418(2)	919(1)	3104(33)	430(1)	951(5)	3154(3)
424(2)	946(0)	3130(7)	425(3)	934(2)	3106(3)	435(3)	966(0)	3155(3)
428(6)	953(1)	3135(4)	435(7)	944(0)	3115(14)	443(2)	973(1)	3156(23)
437(3)	959(3)	3135(7)	449(1)	954(1)	3118(16)	445(5)	982(3)	3158(0)
455(5)	969(3)	3157(1)	455(6)	961(2)	3144(20)	464(6)	986(4)	3183(2)
460(1)	977(1)	3160(4)	467(1)	968(2)	3146(6)	470(0)	997(1)	3183(6)
465(1)	984(1)	3192(4)	469(0)	978(1)	3183(9)	483(0)	1002(0)	3217(5)
476(11)	998(0)	3212(2)	475(5)	991(0)	3200(3)	497(10)	1012(0)	3234(3)
493(1)	1011(0)	3214(1)	491(1)	1001(0)	3205(7)	502(1)	1024(0)	3237(2)
502(5)	1144(0)	3215(4)	505(1)	1141(0)	3205(11)	513(8)	1159(0)	3238(6)
510(1)	1170(0)	3246(3)	508(14)	1169(0)	3239(8)	524(2)	1189(0)	3276(3)
511(5)	1214(9)	3249(3)	510(6)	1208(2)	3241(10)	525(10)	1226(4)	3277(4)

Table S37: Harmonic vibrational frequencies (in cm^{-1}) and infrared intensities (in parentheses in km/mol) for the structure **2-5S-5**.

B3LYP			M06L			wB97XD		
38(1)	511(0)	1220(0)	38(1)	512(24)	1213(0)	39(1)	527(6)	1231(0)
41(0)	522(28)	1231(0)	43(0)	520(4)	1222(0)	45(0)	530(32)	1238(0)
52(1)	524(5)	1234(1)	56(0)	540(31)	1228(3)	57(0)	541(17)	1245(2)
64(0)	542(37)	1245(1)	67(0)	552(15)	1233(0)	68(0)	561(48)	1251(1)
70(2)	576(4)	1269(8)	71(1)	578(62)	1269(10)	76(1)	597(36)	1290(10)
74(0)	578(55)	1290(14)	72(0)	582(6)	1307(18)	77(0)	601(23)	1330(13)
79(0)	584(27)	1329(3)	76(1)	587(14)	1343(1)	82(1)	605(59)	1352(6)
81(0)	596(42)	1392(0)	82(0)	610(92)	1386(0)	86(0)	622(54)	1409(0)
86(0)	626(145)	1409(0)	87(0)	628(146)	1398(0)	88(0)	639(131)	1425(0)
90(1)	635(66)	1439(8)	87(1)	634(32)	1414(6)	94(0)	659(7)	1446(9)
93(0)	640(27)	1467(14)	90(0)	642(16)	1439(12)	95(0)	667(104)	1473(17)
117(0)	657(7)	1485(44)	114(0)	672(8)	1481(23)	119(0)	673(8)	1510(37)
127(0)	701(32)	1492(12)	124(0)	705(35)	1488(4)	129(0)	716(41)	1519(9)
148(1)	702(0)	1517(4)	145(0)	706(2)	1503(3)	153(0)	719(2)	1538(5)
159(0)	721(11)	1527(9)	157(0)	740(10)	1517(10)	164(0)	750(13)	1547(19)
189(0)	736(6)	1534(10)	197(0)	755(3)	1519(6)	203(0)	756(7)	1549(11)
249(5)	755(0)	1535(36)	260(3)	759(0)	1530(25)	271(2)	770(3)	1555(50)
274(0)	762(1)	1691(10)	272(0)	768(0)	1697(11)	279(0)	773(1)	1739(9)
312(5)	763(1)	1711(5)	294(3)	770(2)	1720(4)	326(0)	791(1)	1754(5)
313(4)	809(0)	2021(755)	343(2)	805(0)	2016(671)	332(9)	828(2)	2091(785)
329(1)	811(1)	2055(674)	345(4)	810(3)	2045(658)	344(4)	836(0)	2115(1311)
340(1)	814(3)	2057(1393)	354(0)	814(4)	2053(1208)	354(0)	839(2)	2119(737)
343(2)	838(1)	2066(785)	361(2)	840(1)	2065(698)	371(1)	856(1)	2137(969)
370(15)	895(3)	2108(856)	384(8)	879(7)	2109(768)	398(8)	922(5)	2170(845)
390(0)	906(16)	3112(0)	404(0)	886(1)	3096(29)	406(2)	933(26)	3135(1)
402(1)	927(1)	3118(12)	408(2)	894(83)	3096(7)	416(1)	941(1)	3136(14)
403(3)	936(94)	3127(7)	410(0)	897(12)	3103(36)	424(0)	949(86)	3151(18)
411(0)	937(0)	3129(2)	424(4)	924(1)	3104(0)	427(1)	953(0)	3156(0)
423(5)	946(1)	3132(12)	427(2)	937(0)	3109(41)	434(4)	967(2)	3156(23)
436(2)	956(1)	3135(9)	442(4)	939(0)	3118(20)	445(1)	973(2)	3158(0)
455(8)	962(0)	3158(1)	464(5)	950(3)	3143(20)	472(5)	979(1)	3183(6)
462(5)	964(4)	3161(4)	468(2)	960(0)	3147(6)	474(7)	985(0)	3183(2)
465(1)	971(1)	3211(6)	470(1)	973(1)	3198(1)	484(0)	992(1)	3230(9)
476(21)	993(0)	3213(2)	478(9)	991(0)	3198(17)	493(20)	1010(0)	3231(1)
478(22)	1007(0)	3214(2)	479(12)	1000(0)	3202(4)	497(1)	1020(0)	3236(3)
488(2)	1151(1)	3215(4)	484(0)	1148(1)	3208(11)	503(17)	1166(1)	3239(5)
496(4)	1173(0)	3247(3)	505(3)	1171(0)	3239(7)	516(4)	1191(0)	3277(3)
511(8)	1213(15)	3250(3)	510(0)	1209(6)	3242(9)	525(0)	1229(9)	3277(4)

Table S38: Harmonic vibrational frequencies (in cm^{-1}) and infrared intensities (in parentheses in km/mol) for the structure **2-5T-6**.

B3LYP			M06L			wB97XD		
20(0)	502(6)	1221(0)	26(0)	508(33)	1216(2)	40(0)	501(3)	1227(1)
34(1)	511(1)	1233(0)	41(0)	509(1)	1226(0)	45(0)	515(5)	1237(3)
42(0)	514(11)	1237(2)	45(1)	514(2)	1227(2)	55(0)	522(6)	1250(1)
46(0)	523(13)	1250(1)	50(0)	527(4)	1233(1)	63(0)	525(3)	1262(1)
66(0)	535(18)	1271(6)	66(0)	551(16)	1271(9)	73(0)	532(15)	1280(1)
71(0)	568(10)	1282(10)	72(0)	569(8)	1302(9)	75(0)	554(4)	1314(11)
76(1)	579(47)	1325(8)	76(0)	579(72)	1339(7)	77(0)	595(2)	1350(11)
78(0)	582(39)	1396(1)	81(0)	586(20)	1387(1)	85(0)	600(88)	1416(6)
84(0)	590(28)	1417(4)	84(0)	598(32)	1400(0)	92(0)	623(26)	1429(11)
85(1)	624(5)	1439(7)	91(0)	612(12)	1417(5)	95(0)	639(111)	1446(3)
93(0)	627(110)	1456(56)	94(1)	627(123)	1446(12)	117(0)	661(124)	1466(9)
102(1)	636(83)	1467(10)	110(1)	635(57)	1459(19)	127(0)	706(3)	1471(10)
121(1)	678(16)	1495(18)	119(0)	677(8)	1485(10)	128(1)	719(5)	1524(15)
124(0)	702(23)	1518(5)	133(1)	702(22)	1505(3)	144(1)	734(9)	1541(9)
154(0)	704(2)	1527(12)	154(0)	705(6)	1516(36)	161(0)	739(4)	1549(12)
163(0)	723(3)	1531(36)	173(0)	731(4)	1519(3)	174(0)	749(13)	1573(65)
183(2)	748(4)	1556(18)	225(3)	750(2)	1532(9)	241(1)	753(2)	1579(20)
232(2)	760(1)	1689(10)	242(2)	757(1)	1694(15)	282(0)	764(4)	1731(51)
271(3)	764(0)	1709(7)	273(0)	764(7)	1716(6)	301(4)	775(25)	1742(17)
284(3)	793(4)	2014(862)	288(2)	767(0)	2013(792)	309(11)	788(0)	2081(1567)
316(1)	809(0)	2054(2149)	316(1)	803(0)	2045(615)	321(31)	830(2)	2114(1088)
326(1)	813(1)	2056(852)	331(7)	807(2)	2053(1967)	328(5)	842(0)	2120(759)
339(0)	820(12)	2060(216)	347(0)	817(5)	2059(396)	337(1)	854(6)	2152(886)
342(1)	837(3)	2109(873)	357(1)	837(1)	2110(764)	347(14)	903(2)	2170(709)
357(15)	895(2)	3126(7)	378(1)	879(2)	3106(39)	356(1)	912(13)	3146(7)
384(4)	928(3)	3129(2)	394(0)	884(6)	3108(3)	368(1)	921(36)	3151(17)
387(0)	935(41)	3132(15)	409(0)	890(79)	3108(42)	377(7)	925(49)	3156(2)
398(2)	937(58)	3135(9)	411(3)	924(1)	3112(9)	412(4)	948(3)	3156(16)
405(32)	945(1)	3135(1)	419(1)	937(0)	3118(20)	417(2)	957(2)	3160(1)
410(4)	956(1)	3140(4)	424(6)	938(0)	3131(6)	421(0)	972(0)	3161(1)
413(0)	960(1)	3157(1)	427(1)	954(1)	3144(17)	428(2)	976(1)	3181(3)
426(3)	965(0)	3160(3)	450(19)	962(0)	3147(7)	437(3)	980(0)	3182(1)
449(18)	970(0)	3214(2)	462(7)	969(0)	3203(4)	440(2)	994(1)	3236(3)
460(12)	984(1)	3215(4)	469(3)	980(1)	3208(11)	456(55)	1002(0)	3240(5)
463(38)	1008(0)	3223(3)	470(10)	998(0)	3210(8)	468(3)	1025(0)	3251(2)
465(2)	1155(1)	3240(1)	476(6)	1152(0)	3235(5)	483(1)	1172(0)	3253(2)
478(14)	1176(0)	3247(3)	485(3)	1172(0)	3240(6)	487(49)	1183(0)	3277(2)
483(5)	1214(13)	3250(3)	488(31)	1208(4)	3243(10)	496(7)	1211(1)	3277(5)

Table S39: Harmonic vibrational frequencies (in cm^{-1}) and infrared intensities (in parentheses in km/mol) for the structure **2-5T-7**.

B3LYP			M06L			wB97XD		
39(0)	492(0)	1219(0)	28(0)	505(1)	1219(1)	41(0)	506(57)	1233(1)
42(0)	506(0)	1230(0)	38(1)	509(15)	1221(0)	45(0)	522(1)	1236(0)
51(0)	512(1)	1235(1)	49(0)	513(1)	1229(3)	54(0)	526(14)	1244(2)
64(0)	513(3)	1245(0)	59(0)	522(10)	1235(0)	69(0)	527(0)	1253(0)
72(0)	526(14)	1264(6)	66(0)	529(8)	1270(10)	74(0)	537(5)	1287(8)
74(0)	569(18)	1279(9)	70(0)	564(6)	1294(9)	76(0)	595(32)	1317(9)
77(0)	580(71)	1326(2)	73(0)	579(70)	1341(0)	79(0)	600(62)	1352(3)
85(0)	588(7)	1398(0)	76(0)	583(5)	1393(1)	86(0)	606(25)	1412(0)
90(0)	592(3)	1410(1)	87(1)	606(7)	1403(0)	97(0)	617(26)	1429(1)
91(0)	625(111)	1439(9)	93(0)	625(110)	1418(7)	98(0)	640(94)	1447(9)
98(0)	635(99)	1462(4)	94(0)	635(96)	1446(4)	108(0)	671(113)	1476(8)
115(0)	685(4)	1467(21)	100(0)	678(5)	1450(36)	119(1)	697(4)	1482(21)
124(0)	698(17)	1501(21)	117(1)	696(1)	1487(10)	128(0)	714(20)	1525(19)
131(0)	700(0)	1517(3)	123(1)	702(21)	1505(3)	134(0)	716(1)	1538(5)
161(0)	710(7)	1525(29)	156(0)	727(0)	1521(8)	169(0)	728(6)	1546(36)
176(1)	727(0)	1527(7)	178(1)	743(3)	1529(23)	187(1)	748(0)	1550(14)
229(2)	748(7)	1533(15)	228(0)	753(3)	1551(20)	247(4)	764(7)	1554(19)
246(0)	763(0)	1689(12)	235(1)	769(0)	1695(11)	262(0)	773(0)	1739(11)
271(1)	771(6)	1710(5)	249(3)	775(7)	1718(4)	279(1)	786(10)	1754(5)
282(0)	799(1)	2024(1538)	274(1)	797(2)	2003(1038)	320(1)	817(2)	2087(1675)
324(0)	805(0)	2056(635)	309(2)	800(1)	2045(618)	331(0)	831(0)	2115(949)
342(1)	822(0)	2056(848)	333(1)	814(2)	2054(687)	352(16)	844(0)	2120(679)
346(1)	837(0)	2090(1226)	347(0)	824(6)	2060(1481)	355(0)	854(0)	2155(1214)
350(1)	877(8)	2108(737)	362(2)	842(4)	2109(762)	361(12)	896(17)	2170(720)
353(2)	879(16)	3125(9)	374(1)	865(3)	3106(32)	374(1)	896(7)	3153(11)
365(14)	926(0)	3128(0)	379(10)	887(1)	3106(22)	377(4)	940(1)	3155(9)
385(1)	934(97)	3134(8)	396(2)	894(76)	3109(9)	401(0)	948(93)	3157(3)
391(9)	935(0)	3136(1)	401(1)	923(1)	3120(15)	410(4)	950(0)	3157(16)
395(4)	944(0)	3136(5)	414(0)	940(2)	3133(18)	410(8)	963(0)	3158(5)
413(0)	957(2)	3137(10)	422(4)	941(1)	3139(7)	431(0)	973(1)	3159(4)
417(5)	959(1)	3157(1)	426(3)	952(1)	3144(20)	434(10)	982(1)	3182(2)
428(0)	969(4)	3159(4)	443(10)	959(1)	3147(5)	443(4)	988(4)	3183(6)
429(32)	982(0)	3215(1)	455(6)	973(0)	3201(4)	446(27)	997(0)	3237(2)
456(7)	985(0)	3217(4)	459(12)	982(0)	3210(10)	464(6)	1008(0)	3240(5)
465(1)	1014(1)	3218(0)	469(0)	1003(1)	3228(1)	483(61)	1031(1)	3244(1)
467(62)	1151(0)	3219(3)	472(29)	1153(1)	3233(9)	483(0)	1168(0)	3246(4)
477(16)	1171(0)	3246(3)	476(5)	1172(0)	3240(7)	498(7)	1191(0)	3276(3)
488(33)	1209(13)	3249(4)	488(0)	1211(10)	3241(10)	501(0)	1230(9)	3276(4)

Table S40: Harmonic vibrational frequencies (in cm^{-1}) and infrared intensities (in parentheses in km/mol) for the structure 2-5T-8.

B3LYP			M06L			wB97XD		
21(0)	492(1)	1222(0)	24(0)	496(4)	1213(0)	41(0)	504(0)	1230(0)
34(1)	510(1)	1229(0)	38(0)	509(17)	1220(0)	48(0)	506(83)	1238(0)
43(0)	514(3)	1240(4)	48(1)	510(2)	1238(2)	50(0)	526(0)	1264(0)
47(1)	519(13)	1255(0)	51(0)	525(4)	1243(0)	58(0)	528(11)	1265(1)
66(0)	525(11)	1265(7)	67(0)	536(11)	1276(8)	74(0)	536(13)	1294(6)
69(0)	536(23)	1282(4)	69(0)	555(20)	1288(9)	79(0)	544(4)	1311(6)
77(1)	579(79)	1327(4)	75(0)	579(73)	1340(8)	85(0)	601(88)	1351(8)
82(0)	582(2)	1396(0)	82(0)	585(12)	1387(1)	86(0)	630(3)	1412(0)
86(0)	608(59)	1411(7)	84(0)	612(87)	1395(0)	93(0)	630(127)	1420(0)
92(0)	624(99)	1442(8)	92(1)	624(76)	1419(6)	105(0)	641(68)	1449(9)
93(0)	637(45)	1448(62)	99(0)	636(2)	1447(13)	110(0)	677(61)	1474(20)
107(1)	643(22)	1469(12)	122(0)	640(56)	1452(19)	122(0)	699(2)	1482(9)
117(1)	686(14)	1495(16)	129(1)	688(8)	1488(9)	132(0)	707(21)	1519(16)
129(0)	692(21)	1516(5)	135(1)	697(23)	1504(2)	137(1)	711(0)	1536(3)
155(0)	700(4)	1525(2)	157(0)	701(7)	1515(23)	163(0)	724(6)	1544(13)
158(0)	718(1)	1532(48)	169(0)	721(1)	1520(16)	172(0)	728(1)	1549(37)
163(1)	750(6)	1563(28)	215(2)	752(4)	1531(17)	237(2)	770(2)	1554(25)
220(3)	759(0)	1689(6)	226(2)	758(3)	1695(7)	260(0)	774(15)	1735(7)
271(5)	769(0)	1711(4)	273(0)	760(2)	1717(2)	279(1)	779(0)	1753(4)
280(4)	793(5)	2014(932)	285(2)	772(0)	2015(845)	317(1)	826(0)	2091(1524)
314(1)	806(0)	2054(1544)	312(0)	804(1)	2048(633)	323(5)	832(4)	2114(988)
328(0)	811(8)	2058(1070)	328(4)	806(2)	2055(499)	328(16)	841(1)	2119(678)
338(0)	822(0)	2059(639)	349(0)	823(0)	2058(1796)	349(0)	843(2)	2156(1329)
342(2)	829(2)	2110(836)	361(1)	828(1)	2110(761)	352(0)	892(13)	2171(593)
357(10)	891(0)	3126(8)	385(2)	882(1)	3106(40)	368(7)	893(4)	3149(18)
380(5)	926(3)	3129(2)	394(2)	883(2)	3107(39)	380(1)	939(1)	3154(0)
384(3)	935(29)	3131(13)	409(1)	891(74)	3108(2)	405(21)	949(98)	3155(24)
398(27)	938(62)	3134(8)	412(0)	922(1)	3114(19)	410(2)	954(0)	3157(0)
409(2)	941(1)	3135(2)	424(7)	934(0)	3117(20)	412(8)	963(0)	3158(7)
410(0)	953(1)	3139(7)	428(2)	936(1)	3134(11)	425(1)	973(1)	3159(4)
425(5)	962(0)	3158(1)	445(1)	956(0)	3145(9)	435(8)	982(0)	3182(1)
431(4)	963(0)	3161(1)	454(3)	958(0)	3148(5)	444(8)	988(0)	3182(3)
447(21)	975(0)	3212(2)	457(19)	975(0)	3201(4)	464(7)	992(0)	3234(3)
456(7)	984(0)	3214(4)	468(1)	977(0)	3207(12)	470(8)	1002(0)	3238(6)
462(50)	1008(0)	3224(3)	472(8)	1001(0)	3212(9)	483(0)	1021(0)	3245(1)
464(0)	1150(0)	3240(2)	476(7)	1146(0)	3238(6)	487(23)	1161(1)	3247(4)
476(12)	1179(0)	3247(3)	488(5)	1175(0)	3240(6)	496(0)	1196(0)	3275(3)
483(2)	1217(9)	3250(2)	492(34)	1208(1)	3242(8)	499(12)	1224(2)	3275(2)

Table S41: Harmonic vibrational frequencies (in cm^{-1}) and infrared intensities (in parentheses in km/mol) for the structure **2-5T-9**.

B3LYP			M06L			wB97XD		
-38(0)	515(0)	1218(0)	-53(0)	511(33)	1212(0)	38(0)	508(80)	1232(1)
40(0)	521(29)	1230(1)	42(0)	518(0)	1221(1)	43(0)	524(0)	1239(0)
43(1)	532(32)	1231(0)	44(0)	532(35)	1221(0)	49(0)	525(21)	1252(4)
46(0)	538(18)	1240(1)	45(1)	544(25)	1226(0)	64(0)	529(1)	1256(1)
61(0)	560(1)	1265(9)	61(0)	567(2)	1265(11)	73(0)	532(12)	1292(9)
66(0)	579(58)	1288(5)	65(0)	578(31)	1306(5)	76(0)	592(15)	1320(8)
74(0)	584(13)	1324(12)	72(0)	583(48)	1336(11)	86(0)	599(59)	1348(6)
78(0)	585(24)	1391(0)	74(0)	590(19)	1384(0)	88(0)	605(34)	1410(0)
86(0)	608(69)	1407(0)	83(0)	610(90)	1399(0)	91(0)	615(47)	1427(0)
90(0)	627(91)	1438(8)	84(0)	628(93)	1414(6)	92(0)	643(102)	1447(8)
93(0)	635(85)	1465(17)	92(0)	634(59)	1440(10)	105(0)	658(99)	1475(20)
118(0)	653(5)	1475(44)	114(0)	637(6)	1470(15)	113(0)	693(1)	1490(11)
124(0)	699(0)	1493(15)	120(0)	698(0)	1487(10)	125(0)	709(0)	1518(18)
146(0)	701(28)	1516(0)	143(0)	702(25)	1503(0)	135(0)	713(29)	1537(5)
158(0)	733(2)	1520(25)	156(0)	714(4)	1508(11)	163(0)	725(3)	1546(26)
181(0)	735(3)	1520(13)	185(0)	737(4)	1509(36)	178(2)	748(0)	1548(19)
240(0)	749(1)	1528(7)	249(0)	748(1)	1519(5)	251(1)	766(8)	1555(14)
243(0)	758(1)	1685(15)	249(1)	756(1)	1689(21)	263(0)	774(0)	1736(10)
277(0)	760(0)	1705(5)	276(0)	762(0)	1712(5)	278(1)	775(6)	1752(8)
310(0)	809(0)	2021(844)	300(1)	804(0)	2017(818)	328(1)	824(0)	2091(1345)
328(1)	810(0)	2056(648)	316(2)	807(3)	2045(630)	333(3)	832(0)	2114(1225)
336(0)	815(2)	2058(1630)	344(2)	814(3)	2054(1346)	335(22)	839(1)	2119(673)
340(1)	836(2)	2065(786)	353(1)	837(1)	2065(900)	351(0)	851(1)	2157(1016)
346(3)	889(2)	2109(837)	361(2)	869(2)	2109(730)	353(2)	896(11)	2170(804)
364(3)	898(7)	3126(7)	368(5)	880(7)	3104(39)	365(1)	897(23)	3151(20)
387(0)	927(1)	3129(0)	400(1)	881(1)	3106(0)	371(5)	941(0)	3154(6)
402(4)	935(100)	3132(16)	404(0)	888(83)	3108(46)	401(2)	950(99)	3156(20)
407(0)	935(0)	3135(7)	409(0)	924(1)	3112(22)	409(24)	953(0)	3156(2)
411(0)	945(1)	3136(2)	423(2)	936(0)	3116(5)	411(2)	967(2)	3158(3)
426(2)	957(1)	3139(6)	425(3)	937(0)	3118(20)	421(0)	975(1)	3159(2)
448(1)	962(1)	3157(1)	446(3)	949(1)	3144(18)	427(3)	978(1)	3181(2)
461(4)	964(0)	3160(3)	459(10)	962(0)	3147(6)	435(6)	985(0)	3182(6)
465(14)	968(0)	3214(2)	464(6)	968(0)	3202(4)	445(11)	991(0)	3235(3)
466(0)	991(1)	3216(4)	470(0)	982(0)	3208(11)	473(1)	1006(1)	3239(6)
477(8)	1008(0)	3226(4)	477(3)	999(0)	3213(1)	483(0)	1021(0)	3244(0)
487(2)	1150(1)	3228(0)	485(2)	1149(1)	3215(13)	486(25)	1167(1)	3245(4)
494(8)	1169(0)	3247(3)	502(3)	1167(0)	3240(7)	492(0)	1194(0)	3275(4)
511(0)	1211(13)	3250(3)	510(0)	1208(6)	3243(9)	502(9)	1230(6)	3275(3)

Table S42: Harmonic vibrational frequencies (in cm^{-1}) and infrared intensities (in parentheses in km/mol) for the structure **2-5S-10**.

B3LYP			M06L			wB97XD		
31(0)	499(35)	1225(11)	25(0)	504(3)	1222(11)	29(0)	512(31)	1242(8)
40(0)	504(6)	1234(3)	40(0)	508(35)	1233(1)	41(0)	520(21)	1249(0)
51(0)	522(5)	1241(1)	53(0)	527(3)	1237(9)	53(0)	539(12)	1253(11)
65(0)	541(20)	1258(0)	61(0)	545(31)	1250(1)	61(0)	562(28)	1267(0)
66(1)	552(96)	1270(12)	63(1)	553(55)	1272(16)	68(1)	580(114)	1294(10)
70(0)	571(20)	1283(4)	67(0)	574(34)	1287(11)	73(0)	585(28)	1312(8)
77(0)	585(6)	1326(3)	71(0)	586(4)	1336(2)	75(0)	600(20)	1350(3)
83(0)	591(26)	1393(1)	83(0)	592(30)	1389(2)	85(0)	605(31)	1413(2)
89(0)	605(81)	1437(15)	86(0)	609(69)	1416(15)	91(0)	618(36)	1446(18)
94(1)	608(1)	1442(6)	92(0)	618(5)	1418(1)	95(0)	639(45)	1451(3)
100(0)	665(20)	1464(9)	102(0)	676(20)	1443(8)	102(0)	683(19)	1476(10)
101(0)	689(0)	1470(18)	106(0)	692(2)	1447(17)	106(0)	700(0)	1481(23)
112(0)	693(1)	1484(5)	113(1)	694(1)	1458(5)	119(1)	706(1)	1497(6)
126(1)	705(9)	1503(18)	127(1)	710(10)	1492(10)	127(1)	719(13)	1529(15)
132(0)	707(6)	1524(7)	129(1)	711(9)	1522(6)	135(1)	722(1)	1554(13)
181(1)	743(9)	1685(12)	193(1)	742(9)	1692(12)	193(3)	755(6)	1734(13)
194(1)	753(2)	1692(6)	196(1)	752(2)	1699(7)	202(1)	764(3)	1740(6)
208(2)	763(1)	1704(1)	216(1)	765(16)	1712(2)	224(2)	774(5)	1748(2)
241(6)	767(15)	1717(10)	250(5)	768(2)	1727(9)	249(5)	778(19)	1759(11)
284(2)	777(8)	1937(614)	280(2)	776(5)	1943(475)	290(3)	789(12)	1983(798)
319(0)	798(1)	2024(542)	300(0)	796(0)	2016(563)	327(0)	815(2)	2088(583)
337(1)	816(5)	2033(1002)	319(2)	808(5)	2026(898)	346(0)	839(3)	2092(1168)
340(4)	847(10)	2069(1303)	341(1)	848(7)	2061(1162)	352(7)	864(10)	2136(1269)
348(5)	929(1)	2102(919)	356(6)	885(12)	2096(950)	361(4)	943(1)	2169(985)
360(1)	929(4)	3090(1)	362(3)	890(3)	3063(7)	370(2)	943(5)	3102(1)
380(11)	936(43)	3124(10)	388(9)	895(22)	3100(25)	392(4)	950(37)	3151(11)
400(2)	940(92)	3127(2)	400(1)	898(124)	3105(21)	410(0)	954(116)	3151(12)
404(2)	942(69)	3131(10)	402(2)	928(2)	3115(24)	414(4)	955(42)	3154(6)
406(3)	953(4)	3156(1)	413(0)	941(0)	3142(35)	417(2)	969(6)	3181(3)
413(1)	960(1)	3158(3)	417(2)	948(2)	3145(15)	441(4)	972(0)	3182(10)
431(1)	962(1)	3159(6)	427(3)	952(2)	3146(7)	442(0)	983(2)	3184(3)
438(2)	978(4)	3162(3)	451(1)	972(2)	3148(6)	455(3)	994(5)	3186(3)
469(2)	983(3)	3164(6)	467(2)	975(2)	3151(10)	481(2)	997(1)	3189(8)
479(5)	993(1)	3212(4)	475(6)	989(1)	3207(9)	486(2)	1014(1)	3237(5)
483(4)	1033(1)	3246(3)	483(1)	1028(2)	3239(9)	491(8)	1049(1)	3275(4)
485(3)	1160(0)	3248(4)	488(10)	1161(0)	3240(6)	499(7)	1177(1)	3276(5)
487(7)	1185(0)	3249(4)	492(19)	1183(0)	3241(10)	504(9)	1205(0)	3277(4)
494(40)	1214(17)	3251(3)	496(15)	1213(3)	3243(11)	507(18)	1233(4)	3280(3)

Table S43: Harmonic vibrational frequencies (in cm^{-1}) and infrared intensities (in parentheses in km/mol) for the structure **2-5T-11**.

B3LYP			M06L			wB97XD		
27(0)	487(12)	1229(6)	35(0)	488(13)	1223(3)	30(0)	500(13)	1242(7)
35(0)	492(2)	1234(2)	45(0)	491(1)	1232(3)	43(0)	504(16)	1250(1)
45(0)	496(23)	1241(0)	48(0)	494(3)	1235(5)	47(0)	505(5)	1253(10)
60(0)	498(7)	1258(0)	62(1)	499(19)	1248(0)	61(0)	511(7)	1266(0)
64(0)	501(7)	1270(10)	65(0)	511(15)	1274(15)	65(0)	516(15)	1296(9)
65(0)	518(3)	1283(4)	66(0)	522(19)	1287(7)	67(0)	532(8)	1310(6)
68(0)	542(39)	1327(3)	71(0)	542(31)	1338(3)	73(0)	559(48)	1351(2)
72(0)	568(7)	1388(1)	76(0)	573(4)	1383(4)	78(0)	587(13)	1407(3)
76(0)	584(14)	1438(14)	78(0)	585(3)	1415(12)	81(0)	599(11)	1447(16)
80(0)	589(18)	1443(5)	86(0)	599(31)	1419(3)	86(0)	604(38)	1453(4)
88(1)	605(63)	1464(9)	96(1)	607(66)	1442(8)	96(0)	631(51)	1477(13)
94(0)	692(1)	1470(13)	98(1)	697(1)	1446(16)	100(1)	704(1)	1484(19)
99(0)	694(1)	1472(15)	105(0)	698(2)	1457(8)	105(0)	710(1)	1490(14)
124(1)	700(4)	1511(13)	124(2)	702(1)	1497(6)	129(2)	716(5)	1531(11)
128(1)	706(8)	1525(14)	125(0)	710(13)	1527(14)	130(1)	720(5)	1558(20)
145(0)	713(13)	1684(13)	166(4)	720(4)	1691(13)	151(0)	737(7)	1733(13)
178(3)	746(12)	1691(9)	192(4)	750(10)	1697(10)	193(4)	759(9)	1739(9)
207(1)	757(9)	1703(1)	211(1)	761(14)	1712(1)	224(1)	774(4)	1747(1)
218(0)	764(3)	1716(13)	229(2)	770(2)	1725(11)	232(0)	777(15)	1758(14)
282(1)	776(3)	1961(539)	286(4)	776(3)	1955(381)	293(2)	789(8)	2007(694)
292(3)	799(3)	2020(676)	300(1)	798(3)	2006(712)	301(3)	815(3)	2075(839)
318(0)	810(3)	2051(1077)	304(2)	805(2)	2043(939)	331(1)	832(2)	2114(1134)
323(1)	847(8)	2072(1181)	316(1)	848(6)	2059(1181)	337(1)	864(9)	2135(1142)
336(0)	929(1)	2106(896)	328(1)	888(1)	2098(896)	348(0)	943(1)	2169(879)
337(0)	930(1)	3006(4)	351(0)	892(3)	2974(5)	350(0)	944(4)	3013(5)
349(1)	936(49)	3118(10)	355(3)	895(38)	3092(20)	365(3)	950(56)	3142(16)
365(2)	940(132)	3124(14)	369(2)	900(126)	3101(36)	382(3)	955(49)	3148(11)
370(8)	942(29)	3126(1)	376(4)	941(1)	3102(21)	388(10)	957(94)	3153(6)
402(2)	954(4)	3155(1)	400(0)	944(2)	3142(35)	410(1)	974(0)	3181(2)
403(2)	960(0)	3158(1)	402(1)	949(1)	3143(13)	414(1)	976(4)	3182(10)
411(25)	969(0)	3158(8)	416(28)	960(0)	3145(7)	431(41)	990(0)	3184(2)
412(19)	976(3)	3161(3)	416(24)	971(0)	3147(4)	437(18)	998(5)	3186(3)
427(4)	984(1)	3167(4)	425(2)	976(1)	3160(10)	448(6)	1000(1)	3202(5)
434(7)	985(1)	3199(5)	439(4)	983(1)	3195(10)	458(1)	1006(2)	3225(5)
445(12)	1019(2)	3245(3)	446(8)	1008(2)	3238(10)	464(19)	1029(2)	3275(4)
469(3)	1157(0)	3247(3)	471(4)	1156(0)	3239(6)	480(4)	1172(1)	3276(5)
479(1)	1186(0)	3248(4)	475(2)	1184(0)	3241(9)	489(2)	1207(0)	3277(4)
484(1)	1218(18)	3250(3)	481(2)	1219(6)	3242(9)	494(2)	1239(4)	3279(3)

Table S44: Harmonic vibrational frequencies (in cm^{-1}) and infrared intensities (in parentheses in km/mol) for the structure **2-5T-12**.

B3LYP			M06L			wB97XD		
31(0)	516(1)	1215(0)	13(0)	504(8)	1205(1)	26(0)	532(0)	1226(2)
34(0)	522(47)	1220(1)	35(0)	508(0)	1216(1)	37(0)	533(22)	1229(0)
56(0)	528(25)	1238(4)	52(0)	515(20)	1234(2)	56(0)	541(14)	1246(3)
65(0)	529(1)	1245(0)	55(0)	526(2)	1244(5)	59(0)	544(28)	1253(0)
65(0)	554(11)	1252(4)	64(0)	541(29)	1254(9)	70(0)	573(13)	1269(6)
70(0)	577(68)	1307(11)	65(0)	570(8)	1317(7)	71(0)	595(0)	1342(15)
76(0)	581(1)	1319(11)	68(0)	573(63)	1338(8)	79(0)	597(77)	1351(11)
84(0)	582(13)	1385(0)	76(1)	582(2)	1390(1)	84(0)	608(40)	1403(0)
91(0)	600(16)	1414(0)	83(0)	589(9)	1412(5)	90(0)	614(6)	1434(0)
102(1)	619(98)	1434(7)	90(2)	617(110)	1412(1)	113(0)	634(117)	1441(8)
105(0)	631(99)	1463(6)	97(0)	625(90)	1440(9)	117(0)	658(20)	1470(14)
119(0)	649(17)	1483(11)	115(1)	676(5)	1461(13)	123(1)	663(94)	1505(17)
122(1)	688(0)	1506(6)	124(1)	704(1)	1494(8)	129(0)	700(0)	1527(4)
138(0)	707(23)	1515(3)	157(1)	712(20)	1505(4)	144(0)	724(25)	1536(14)
173(1)	723(1)	1517(9)	162(0)	735(3)	1525(15)	180(0)	743(1)	1537(36)
179(1)	736(0)	1518(22)	170(1)	744(2)	1526(6)	190(0)	752(1)	1539(1)
227(0)	750(2)	1526(8)	229(3)	757(4)	1555(13)	249(0)	760(4)	1553(14)
253(0)	755(3)	1683(54)	233(2)	760(5)	1692(27)	265(0)	774(5)	1735(28)
283(0)	772(0)	1701(10)	242(3)	780(2)	1713(9)	297(0)	790(0)	1748(10)
308(0)	806(0)	2020(210)	267(1)	795(2)	2001(472)	316(1)	822(0)	2075(258)
328(1)	812(0)	2054(407)	290(5)	808(0)	2031(982)	329(1)	841(0)	2113(577)
339(0)	822(0)	2064(1110)	316(0)	821(1)	2049(689)	353(0)	847(0)	2126(1193)
352(1)	842(2)	2069(1229)	348(0)	824(1)	2064(1057)	373(1)	861(3)	2126(1170)
359(1)	898(1)	2111(1259)	369(1)	847(5)	2109(1021)	383(2)	920(1)	2172(1286)
370(3)	904(12)	3123(0)	376(4)	873(7)	3100(39)	384(1)	924(9)	3145(21)
388(0)	914(1)	3125(10)	390(4)	887(0)	3107(29)	405(0)	933(1)	3145(1)
405(0)	923(0)	3125(13)	393(2)	893(77)	3113(16)	415(0)	940(0)	3146(11)
409(4)	929(103)	3129(0)	402(1)	917(0)	3116(20)	431(0)	947(96)	3151(10)
415(0)	951(3)	3131(18)	407(4)	934(1)	3131(15)	434(0)	961(4)	3159(15)
421(0)	952(1)	3133(1)	410(0)	942(3)	3137(14)	435(2)	970(2)	3161(1)
443(0)	967(4)	3156(1)	428(1)	950(2)	3141(32)	455(1)	990(4)	3181(10)
455(5)	967(0)	3159(5)	440(11)	956(1)	3146(7)	463(6)	992(0)	3181(2)
466(0)	987(0)	3207(2)	453(9)	977(1)	3194(6)	481(17)	1006(0)	3230(3)
468(13)	994(0)	3209(4)	458(12)	991(0)	3206(9)	486(0)	1008(0)	3234(5)
470(4)	1015(0)	3218(5)	465(1)	1010(0)	3224(4)	488(2)	1028(0)	3242(7)
493(14)	1156(0)	3219(1)	467(5)	1155(0)	3234(7)	502(1)	1174(0)	3242(1)
494(1)	1159(0)	3247(3)	479(28)	1165(0)	3239(7)	514(31)	1176(0)	3275(4)
511(0)	1182(26)	3249(3)	483(2)	1179(19)	3241(11)	524(0)	1207(27)	3275(4)

Table S45:Harmonic vibrational frequencies (in cm^{-1}) and infrared intensities (in parentheses in km/mol) for the structure **3-9S-1**.

B3LYP			M06L			wB97XD		
37(0)	466(0)	1010(1)	35(1)	470(0)	1005(1)	37(0)	485(0)	1026(1)
41(0)	479(18)	1010(1)	41(0)	480(10)	1005(1)	43(0)	503(10)	1026(1)
41(0)	479(18)	1142(0)	41(0)	480(10)	1139(0)	43(0)	503(10)	1158(0)
49(0)	488(0)	1142(0)	50(0)	487(0)	1139(0)	53(0)	513(0)	1158(0)
49(0)	510(1)	1207(3)	50(0)	509(0)	1204(4)	53(0)	524(0)	1222(3)
54(0)	511(0)	1218(5)	53(0)	509(0)	1205(4)	57(0)	524(0)	1225(5)
71(0)	511(0)	1218(5)	65(0)	509(0)	1208(5)	73(0)	524(0)	1225(5)
73(0)	520(0)	1224(0)	71(0)	509(34)	1209(0)	76(0)	529(36)	1230(0)
73(0)	521(31)	1224(0)	71(0)	509(34)	1209(0)	76(0)	529(36)	1230(0)
79(0)	521(31)	1266(15)	72(0)	509(1)	1288(15)	80(0)	529(0)	1310(13)
79(0)	535(0)	1266(15)	72(0)	537(0)	1288(15)	80(0)	548(0)	1310(13)
82(0)	535(0)	1312(0)	79(0)	537(0)	1332(0)	86(0)	548(0)	1340(0)
85(0)	561(0)	1386(0)	82(0)	567(0)	1383(1)	88(0)	582(0)	1402(0)
88(0)	579(194)	1396(0)	84(0)	579(183)	1392(0)	89(0)	600(0)	1418(0)
88(0)	579(0)	1396(0)	84(0)	580(0)	1392(0)	89(0)	600(0)	1419(0)
95(1)	579(0)	1494(32)	92(1)	580(0)	1485(20)	98(0)	600(216)	1515(31)
95(1)	591(19)	1494(32)	92(1)	587(16)	1485(20)	98(0)	611(26)	1515(31)
101(0)	595(3)	1502(0)	98(0)	602(5)	1494(0)	104(0)	615(2)	1527(0)
122(0)	595(3)	1513(0)	117(0)	602(5)	1498(0)	124(0)	615(2)	1533(0)
122(0)	623(322)	1513(0)	117(0)	623(321)	1498(0)	124(0)	638(342)	1533(0)
154(0)	623(322)	1521(11)	150(0)	623(321)	1509(6)	156(0)	638(342)	1540(13)
160(0)	626(0)	1526(12)	158(0)	626(0)	1518(13)	165(0)	641(0)	1546(19)
160(0)	635(0)	1526(12)	158(0)	635(0)	1518(13)	165(0)	658(0)	1546(19)
160(0)	641(19)	1529(0)	164(0)	645(18)	1525(0)	169(0)	668(53)	1551(0)
246(1)	641(19)	2054(0)	255(2)	645(18)	2043(0)	264(0)	668(53)	2117(0)
246(1)	712(0)	2054(0)	255(2)	718(0)	2043(0)	264(0)	737(0)	2118(0)
270(0)	712(0)	2056(1864)	269(0)	718(0)	2045(1826)	274(0)	737(0)	2118(0)
333(0)	748(3)	2058(0)	352(0)	749(4)	2054(0)	347(0)	766(4)	2120(1988)
339(0)	798(12)	2061(1377)	354(0)	797(0)	2058(1358)	354(0)	809(0)	2120(1571)
339(0)	798(12)	2061(1377)	354(0)	797(0)	2058(1358)	354(0)	821(12)	2120(1571)
340(0)	799(0)	2107(1415)	358(1)	799(0)	2108(1262)	358(0)	821(12)	2169(1360)
340(0)	799(0)	2107(1414)	358(1)	800(16)	2108(1263)	358(0)	828(0)	2169(1360)
359(5)	799(0)	2115(0)	383(3)	800(16)	2115(0)	390(4)	828(0)	2175(0)
379(0)	808(1)	3127(3)	397(0)	806(3)	3102(30)	395(0)	836(1)	3151(18)
405(0)	817(0)	3130(0)	410(0)	816(0)	3105(0)	424(0)	848(0)	3151(18)
407(4)	823(5)	3132(13)	410(0)	824(4)	3107(37)	424(0)	852(4)	3152(0)
407(4)	823(5)	3132(13)	411(0)	824(4)	3107(36)	425(0)	852(4)	3157(7)
408(0)	942(0)	3134(0)	425(5)	927(0)	3107(0)	431(1)	964(5)	3157(0)
408(0)	942(0)	3136(0)	427(0)	927(0)	3116(0)	431(1)	964(0)	3157(0)
424(4)	948(5)	3136(0)	427(0)	927(9)	3116(0)	437(4)	964(0)	3158(21)
430(0)	959(2)	3136(15)	431(3)	944(1)	3116(35)	439(0)	984(4)	3161(0)

430(0)	961(4)	3214(2)	431(3)	944(1)	3202(4)	439(0)	986(6)	3237(3)
461(4)	961(4)	3214(2)	464(5)	944(0)	3202(4)	472(5)	986(6)	3237(3)
461(4)	961(0)	3214(0)	464(5)	956(0)	3202(0)	472(5)	986(0)	3237(0)
464(0)	965(0)	3216(0)	467(0)	959(0)	3207(0)	477(0)	993(0)	3240(0)
465(2)	965(0)	3216(0)	469(1)	960(0)	3207(0)	485(0)	993(0)	3240(0)
466(0)	1001(0)	3216(11)	470(0)	993(0)	3207(31)	485(0)	1016(0)	3240(14)

Table S46:Harmonic vibrational frequencies (in cm^{-1}) and infrared intensities (in parentheses in km/mol) for the structure **3-9S-2**.

B3LYP			M06L			wB97XD		
10(0)	464(1)	1011(0)	31(0)	468(1)	999(0)	27(0)	484(0)	1021(0)
37(0)	473(15)	1021(0)	34(0)	475(5)	1011(1)	40(0)	491(9)	1032(0)
43(0)	479(17)	1125(0)	39(0)	485(16)	1120(0)	41(0)	512(19)	1140(0)
48(0)	488(12)	1145(0)	49(0)	493(5)	1145(0)	51(0)	515(1)	1162(0)
54(0)	501(0)	1180(18)	52(0)	498(2)	1169(10)	54(0)	517(6)	1195(14)
57(0)	506(0)	1199(4)	54(0)	507(27)	1189(0)	57(0)	525(0)	1210(0)
63(0)	509(0)	1204(0)	63(0)	508(1)	1200(5)	67(0)	527(11)	1214(3)
63(0)	518(4)	1227(4)	66(0)	512(2)	1224(0)	68(0)	529(13)	1239(0)
76(0)	519(22)	1231(2)	68(0)	515(19)	1225(0)	77(0)	534(23)	1245(0)
79(0)	521(0)	1237(0)	78(0)	518(5)	1247(17)	82(0)	538(0)	1268(9)
81(0)	529(24)	1287(16)	82(0)	518(7)	1308(16)	86(0)	540(3)	1328(13)
82(0)	532(2)	1299(4)	82(0)	526(2)	1323(2)	87(0)	541(2)	1336(7)
86(0)	543(3)	1370(0)	84(0)	545(3)	1373(0)	89(0)	562(0)	1394(0)
91(0)	577(5)	1377(0)	87(0)	576(2)	1375(0)	94(0)	595(1)	1399(0)
94(0)	578(89)	1403(0)	90(0)	577(10)	1400(0)	98(0)	596(136)	1428(0)
98(0)	579(95)	1482(24)	98(0)	577(157)	1478(14)	103(0)	599(68)	1506(28)
110(0)	579(4)	1504(1)	115(0)	589(14)	1487(12)	117(0)	606(20)	1522(12)
123(0)	604(0)	1507(11)	118(0)	610(1)	1488(5)	126(0)	629(173)	1525(9)
131(1)	613(95)	1508(0)	135(0)	615(174)	1492(1)	143(0)	631(25)	1527(1)
136(0)	619(18)	1514(4)	145(1)	617(20)	1496(1)	149(0)	634(16)	1531(0)
140(0)	622(322)	1515(12)	153(0)	621(308)	1503(9)	155(0)	640(271)	1535(15)
154(0)	625(163)	1518(2)	157(0)	624(102)	1512(6)	163(0)	644(186)	1542(8)
167(1)	631(10)	1524(6)	172(1)	634(17)	1521(1)	180(1)	648(22)	1547(7)
177(0)	634(23)	1529(16)	179(0)	636(1)	1533(9)	186(0)	659(34)	1562(15)
181(1)	661(4)	2046(2)	185(1)	661(4)	2037(57)	194(0)	688(5)	2108(31)
255(2)	676(4)	2055(786)	263(1)	674(3)	2046(513)	269(0)	699(8)	2113(1151)
262(0)	717(0)	2056(564)	266(5)	718(0)	2050(856)	279(1)	744(1)	2119(817)
328(0)	749(5)	2061(1399)	337(0)	748(6)	2058(1268)	341(1)	770(6)	2120(1177)
330(0)	767(25)	2068(28)	345(0)	768(28)	2065(241)	346(0)	788(24)	2124(28)
334(0)	785(0)	2083(1236)	347(0)	779(2)	2065(998)	349(0)	810(0)	2141(1327)
334(0)	802(2)	2104(1071)	350(0)	802(4)	2104(991)	360(0)	813(2)	2162(747)
363(0)	810(2)	2108(1220)	369(0)	803(1)	2109(1267)	381(0)	834(3)	2169(1373)
372(4)	810(1)	2127(957)	382(3)	808(5)	2124(695)	400(3)	836(1)	2186(992)
389(2)	818(4)	3113(14)	397(2)	812(3)	3092(38)	412(2)	842(5)	3136(19)

398(0)	824(0)	3120(6)	401(2)	821(2)	3094(19)	416(0)	851(1)	3138(13)
399(2)	829(1)	3120(10)	402(2)	828(1)	3104(43)	421(0)	855(0)	3145(9)
409(0)	835(1)	3125(4)	414(1)	834(1)	3106(15)	432(1)	855(2)	3146(7)
412(2)	906(0)	3128(13)	423(1)	907(0)	3107(34)	435(2)	936(0)	3148(19)
426(1)	946(1)	3131(1)	425(1)	923(1)	3108(9)	438(0)	971(4)	3155(8)
428(0)	950(1)	3134(14)	431(1)	931(0)	3111(4)	442(0)	974(1)	3162(12)
435(1)	957(1)	3136(2)	437(3)	932(0)	3116(18)	454(2)	976(0)	3165(1)
439(1)	965(0)	3196(2)	447(0)	938(2)	3190(4)	456(2)	986(1)	3223(2)
454(5)	971(1)	3199(5)	459(4)	945(0)	3192(3)	466(1)	992(1)	3224(3)
460(0)	975(1)	3200(2)	462(5)	953(4)	3197(0)	470(5)	997(7)	3227(5)
461(4)	979(7)	3203(5)	463(3)	965(1)	3197(23)	477(2)	998(3)	3228(7)
463(1)	986(0)	3211(1)	465(1)	983(0)	3201(3)	480(0)	1006(0)	3235(2)
463(2)	998(0)	3214(4)	467(0)	990(0)	3207(11)	482(1)	1013(0)	3239(5)

Table S47: Harmonic vibrational frequencies (in cm^{-1}) and infrared intensities (in parentheses in km/mol) for the structure **3-8S-1**.

B3LYP			M06L			wB97XD		
26(0)	487(8)	1007(1)	20(0)	483(11)	1001(1)	25(0)	504(0)	1022(1)
40(0)	491(32)	1141(0)	38(0)	487(51)	1135(0)	43(0)	509(26)	1156(0)
48(0)	492(17)	1165(0)	48(0)	493(4)	1157(0)	53(0)	509(42)	1182(1)
52(1)	496(1)	1201(6)	51(1)	501(1)	1189(2)	55(1)	517(12)	1211(5)
59(0)	510(0)	1214(2)	55(0)	508(27)	1201(0)	61(0)	523(0)	1224(0)
65(0)	516(11)	1219(1)	59(0)	510(0)	1213(2)	66(0)	528(28)	1230(1)
73(0)	522(19)	1233(0)	70(0)	519(12)	1219(0)	76(0)	535(7)	1239(0)
78(0)	526(9)	1247(1)	72(0)	531(8)	1230(0)	80(0)	543(13)	1254(0)
80(0)	534(9)	1262(8)	77(0)	534(0)	1277(15)	84(0)	546(1)	1303(10)
86(0)	547(5)	1306(13)	81(0)	546(11)	1323(12)	89(0)	565(16)	1348(15)
89(0)	558(114)	1335(4)	84(0)	557(59)	1349(4)	92(0)	577(139)	1363(5)
96(0)	560(65)	1390(0)	90(0)	561(153)	1384(0)	98(0)	578(83)	1410(0)
104(0)	578(58)	1413(3)	99(0)	579(77)	1398(2)	108(0)	599(91)	1428(3)
106(0)	579(28)	1425(0)	99(0)	584(6)	1408(0)	109(0)	602(4)	1440(0)
135(0)	589(1)	1473(14)	131(1)	590(9)	1474(9)	139(0)	604(13)	1497(14)
149(1)	600(17)	1481(20)	146(1)	603(17)	1479(13)	154(1)	618(9)	1506(27)
153(1)	610(14)	1486(6)	157(1)	613(89)	1486(1)	160(1)	627(11)	1513(3)
159(1)	618(60)	1516(4)	168(0)	620(144)	1500(3)	165(2)	639(258)	1536(5)
171(0)	624(223)	1526(6)	178(0)	623(148)	1513(4)	184(0)	641(138)	1544(8)
178(1)	629(134)	1535(26)	183(1)	630(39)	1517(16)	191(1)	657(59)	1552(31)
189(1)	634(11)	1539(8)	196(1)	635(6)	1521(7)	201(0)	665(24)	1556(13)
196(0)	649(5)	1552(17)	209(0)	649(7)	1535(14)	209(2)	684(9)	1576(16)
242(14)	698(3)	1556(11)	246(9)	691(3)	1539(5)	253(22)	716(4)	1577(9)
268(4)	719(1)	1893(437)	265(2)	718(0)	1901(373)	273(3)	747(0)	1943(472)
274(5)	747(3)	2028(19)	281(10)	746(4)	2025(8)	298(3)	767(6)	2089(91)
288(3)	778(0)	2053(709)	323(2)	779(0)	2047(387)	328(1)	799(0)	2110(972)

300(4)	781(1)	2059(840)	329(4)	780(3)	2055(1147)	334(2)	808(7)	2114(1087)
325(8)	790(8)	2060(1205)	350(1)	787(9)	2059(876)	348(1)	812(2)	2121(952)
330(19)	794(0)	2063(882)	355(0)	794(1)	2066(1090)	353(0)	824(4)	2122(925)
337(2)	803(0)	2091(2120)	361(2)	807(4)	2095(2022)	357(2)	832(1)	2151(2033)
344(6)	812(3)	2113(578)	366(3)	812(2)	2113(476)	372(4)	838(5)	2174(619)
345(14)	816(2)	3130(4)	376(9)	815(2)	3109(37)	382(45)	845(1)	3152(17)
347(10)	820(5)	3132(8)	379(9)	820(4)	3109(26)	388(12)	848(6)	3157(7)
376(1)	831(3)	3134(6)	387(1)	832(2)	3112(24)	395(9)	852(2)	3158(14)
396(101)	931(0)	3136(7)	408(32)	920(1)	3113(6)	410(114)	956(0)	3159(7)
402(7)	942(3)	3141(8)	410(10)	923(0)	3113(34)	425(1)	964(2)	3162(11)
409(0)	946(2)	3143(4)	412(24)	924(2)	3119(17)	426(0)	973(4)	3165(1)
415(4)	949(7)	3155(1)	419(17)	934(2)	3126(8)	431(10)	976(0)	3173(2)
427(1)	954(1)	3155(2)	425(2)	938(1)	3129(14)	438(1)	978(0)	3173(5)
443(14)	955(0)	3215(1)	447(29)	939(6)	3204(3)	452(6)	983(5)	3238(2)
451(6)	957(3)	3217(3)	459(2)	943(1)	3206(4)	464(6)	989(3)	3241(4)
455(4)	960(4)	3225(1)	461(1)	954(1)	3207(6)	471(6)	994(5)	3243(3)
462(2)	973(0)	3225(1)	463(8)	970(0)	3210(11)	482(3)	995(1)	3244(2)
464(0)	992(2)	3241(1)	468(0)	986(1)	3220(4)	483(0)	1009(2)	3261(1)
468(6)	1003(0)	3242(1)	471(3)	997(0)	3223(5)	485(2)	1017(0)	3262(1)

Table S48: Harmonic vibrational frequencies (in cm^{-1}) and infrared intensities (in parentheses in km/mol) for the structure 3-8S-2.

B3LYP			M06L			wB97XD		
36(0)	478(17)	1011(1)	36(0)	479(9)	1002(0)	38(0)	502(10)	1026(1)
38(0)	484(8)	1140(0)	37(0)	484(3)	1135(0)	39(0)	507(8)	1156(0)
40(0)	505(6)	1143(0)	41(0)	505(5)	1140(0)	43(0)	516(8)	1160(0)
46(0)	509(1)	1206(3)	49(0)	508(20)	1200(4)	51(0)	523(0)	1221(4)
49(0)	510(0)	1214(5)	50(1)	508(6)	1205(3)	53(0)	523(0)	1222(3)
54(0)	519(10)	1220(4)	55(0)	509(11)	1207(2)	57(0)	529(21)	1228(3)
71(0)	521(32)	1223(1)	67(0)	509(14)	1208(2)	74(0)	530(31)	1229(1)
72(0)	529(7)	1226(0)	72(0)	532(13)	1213(1)	76(0)	540(12)	1233(0)
76(0)	535(27)	1254(7)	73(0)	534(20)	1275(7)	79(0)	548(26)	1296(7)
79(0)	537(3)	1270(14)	74(0)	540(4)	1290(14)	80(0)	550(2)	1313(13)
82(0)	560(8)	1308(2)	80(0)	568(9)	1327(2)	86(0)	573(24)	1337(2)
85(0)	567(33)	1384(1)	83(0)	578(63)	1380(1)	87(0)	580(14)	1400(0)
87(0)	579(41)	1392(0)	85(0)	578(82)	1388(0)	89(0)	600(1)	1414(0)
89(1)	579(94)	1399(0)	89(1)	581(11)	1394(0)	93(0)	600(159)	1421(0)
92(1)	590(7)	1483(24)	93(1)	589(1)	1474(16)	94(1)	610(9)	1506(22)
98(0)	595(4)	1492(27)	96(0)	598(6)	1483(19)	101(0)	617(19)	1516(27)
112(0)	605(23)	1499(12)	106(0)	610(48)	1489(3)	114(0)	621(77)	1524(9)
120(0)	617(156)	1513(1)	115(0)	619(193)	1492(0)	123(0)	634(188)	1529(2)
146(0)	624(195)	1513(1)	146(0)	625(152)	1497(1)	152(0)	640(140)	1534(2)
153(0)	630(58)	1518(7)	153(0)	631(47)	1505(6)	161(0)	651(35)	1539(10)

157(0)	637(56)	1523(20)	156(0)	640(50)	1515(12)	162(0)	668(44)	1545(26)
161(0)	639(44)	1525(10)	160(0)	642(44)	1519(14)	167(0)	671(117)	1547(17)
234(1)	697(2)	1528(3)	244(1)	699(3)	1525(3)	253(1)	720(3)	1553(3)
249(1)	715(0)	2029(891)	258(2)	716(1)	2019(805)	270(0)	740(1)	2092(1062)
270(0)	731(0)	2055(4)	268(1)	733(1)	2044(30)	275(0)	758(1)	2118(620)
294(1)	751(7)	2056(1266)	306(1)	751(6)	2046(1206)	306(1)	770(9)	2119(156)
332(0)	791(10)	2059(601)	347(0)	791(7)	2056(579)	347(0)	811(1)	2120(1472)
338(0)	796(2)	2061(1425)	350(0)	792(1)	2058(1491)	354(1)	813(6)	2121(1391)
339(0)	799(0)	2076(1263)	352(1)	797(2)	2070(1140)	355(0)	823(6)	2140(1184)
340(1)	801(5)	2107(1434)	355(1)	799(5)	2108(1276)	359(0)	824(8)	2170(1372)
354(3)	805(11)	2113(295)	379(2)	802(5)	2113(280)	381(2)	828(6)	2174(305)
384(2)	806(1)	3109(11)	398(1)	807(11)	3083(30)	401(1)	834(1)	3132(15)
402(1)	819(3)	3127(4)	406(1)	816(5)	3103(29)	416(0)	844(3)	3149(14)
404(1)	824(5)	3129(7)	410(1)	824(4)	3104(34)	426(0)	852(2)	3151(12)
408(1)	897(7)	3131(8)	415(0)	886(5)	3105(20)	428(0)	924(6)	3156(5)
410(2)	937(0)	3133(5)	424(3)	922(0)	3106(2)	432(1)	960(1)	3157(2)
422(3)	945(3)	3136(3)	427(0)	923(3)	3115(12)	436(2)	963(3)	3157(2)
428(6)	950(1)	3136(8)	430(2)	940(1)	3116(13)	440(0)	972(1)	3158(17)
429(2)	957(2)	3138(2)	437(7)	942(2)	3117(9)	443(5)	978(3)	3162(1)
440(2)	960(3)	3192(3)	452(3)	948(1)	3183(8)	448(3)	985(4)	3216(4)
459(1)	962(1)	3213(2)	463(4)	956(0)	3200(3)	470(2)	987(1)	3235(2)
462(3)	964(1)	3214(2)	466(2)	960(0)	3201(3)	473(3)	992(2)	3236(2)
463(1)	986(3)	3216(1)	468(0)	972(4)	3206(4)	477(1)	1003(2)	3239(4)
465(1)	997(1)	3216(6)	469(1)	986(0)	3206(8)	484(0)	1011(1)	3240(1)
466(0)	1006(1)	3217(1)	470(0)	998(1)	3207(14)	485(0)	1020(1)	3240(6)

Table S49: Harmonic vibrational frequencies (in cm^{-1}) and infrared intensities (in parentheses in km/mol) for the structure **3-8T-3.

B3LYP			M06L			wB97XD		
24(1)	459(8)	1011(0)	28(0)	463(7)	1003(0)	24(1)	475(6)	1025(0)
39(0)	462(2)	1148(1)	40(0)	464(2)	1144(0)	42(0)	478(4)	1164(1)
46(1)	465(0)	1163(0)	49(1)	469(0)	1162(0)	49(0)	485(0)	1181(0)
48(1)	470(5)	1205(10)	51(0)	471(4)	1199(8)	50(1)	487(5)	1224(0)
54(0)	480(6)	1213(9)	55(0)	479(1)	1205(1)	57(0)	498(4)	1226(12)
66(0)	491(5)	1226(3)	66(0)	486(7)	1216(1)	68(1)	507(2)	1235(2)
68(0)	499(26)	1229(1)	69(0)	496(11)	1225(5)	73(0)	512(29)	1243(6)
71(0)	508(3)	1244(0)	70(0)	505(1)	1233(1)	74(1)	521(4)	1252(0)
75(1)	511(1)	1260(1)	72(0)	507(14)	1260(6)	77(2)	524(0)	1281(3)
79(0)	512(2)	1288(12)	75(0)	509(7)	1305(13)	80(0)	525(5)	1328(14)
81(0)	521(26)	1327(2)	78(0)	517(27)	1344(1)	84(0)	531(29)	1356(3)
85(0)	527(5)	1391(0)	84(0)	526(19)	1386(1)	87(0)	537(6)	1410(1)
91(1)	536(8)	1413(3)	88(1)	539(9)	1402(6)	94(1)	553(9)	1430(4)
97(0)	569(37)	1431(15)	93(0)	565(31)	1411(3)	99(0)	584(44)	1443(15)

107(0)	572(59)	1460(16)	108(1)	578(29)	1440(12)	111(0)	593(70)	1473(20)
114(0)	579(71)	1489(25)	117(0)	580(50)	1483(16)	119(0)	600(76)	1514(25)
124(0)	581(113)	1495(4)	129(0)	584(153)	1491(5)	128(0)	601(123)	1522(6)
130(0)	592(24)	1517(4)	135(0)	594(46)	1503(3)	136(0)	614(64)	1537(7)
135(0)	603(6)	1527(9)	146(0)	601(27)	1516(9)	145(1)	622(11)	1547(19)
153(3)	615(202)	1529(7)	159(0)	613(143)	1519(4)	158(4)	636(204)	1548(6)
162(1)	630(69)	1532(9)	168(1)	630(102)	1524(5)	168(2)	657(63)	1553(7)
170(1)	637(73)	1578(32)	178(1)	637(38)	1562(23)	180(0)	666(76)	1606(41)
186(8)	694(8)	1684(11)	194(2)	693(9)	1685(9)	194(18)	710(10)	1732(13)
218(10)	709(1)	1930(433)	221(4)	708(0)	1928(410)	226(13)	730(1)	1983(501)
250(14)	727(5)	2016(306)	253(12)	726(3)	2002(48)	271(16)	751(6)	2071(491)
271(6)	750(2)	2042(931)	273(1)	749(3)	2031(1033)	278(1)	771(3)	2107(994)
287(15)	757(2)	2057(691)	286(7)	760(5)	2047(664)	305(42)	773(3)	2119(836)
316(0)	772(7)	2062(964)	307(1)	768(4)	2058(928)	329(2)	799(7)	2121(1078)
326(10)	801(1)	2064(1427)	344(2)	796(1)	2061(1453)	339(22)	828(3)	2122(1288)
333(1)	806(6)	2095(1476)	352(1)	803(10)	2091(1369)	346(10)	831(4)	2155(1409)
339(1)	810(1)	2112(687)	353(1)	808(1)	2112(663)	355(2)	835(2)	2173(706)
341(2)	813(1)	3125(4)	362(6)	810(3)	3099(18)	359(5)	838(1)	3153(17)
346(5)	825(2)	3129(1)	367(2)	823(1)	3106(13)	369(46)	852(2)	3155(5)
366(54)	833(0)	3133(13)	374(30)	831(0)	3109(41)	380(62)	854(0)	3155(9)
372(53)	911(38)	3136(7)	383(4)	865(34)	3112(23)	393(3)	927(39)	3157(10)
377(4)	923(4)	3139(6)	388(2)	880(3)	3119(16)	400(5)	946(0)	3159(2)
398(2)	927(0)	3141(3)	392(20)	922(0)	3119(11)	412(1)	951(0)	3160(8)
402(4)	946(2)	3162(1)	403(3)	925(1)	3144(9)	419(11)	967(3)	3185(1)
403(8)	953(0)	3166(1)	407(5)	936(0)	3149(5)	425(1)	974(1)	3187(3)
411(1)	956(1)	3215(2)	412(3)	938(1)	3204(4)	427(0)	979(1)	3238(2)
414(0)	958(1)	3217(3)	421(1)	941(2)	3206(3)	433(3)	979(1)	3240(2)
425(4)	961(3)	3220(1)	424(1)	954(2)	3209(9)	439(1)	990(3)	3241(4)
426(10)	972(5)	3222(2)	428(15)	958(1)	3211(8)	449(5)	996(4)	3244(3)
429(3)	979(0)	3251(2)	435(2)	978(0)	3241(2)	457(9)	1001(1)	3278(1)
448(42)	1002(0)	3255(0)	452(39)	994(0)	3245(6)	460(39)	1016(0)	3279(2)

Table S50: Harmonic vibrational frequencies (in cm^{-1}) and infrared intensities (in parentheses in km/mol) for the structure 3-8S-4.

B3LYP			M06L			wB97XD		
-56(0)	475(15)	1013(1)	-18(0)	480(1)	1005(0)	-47(0)	493(20)	1027(1)
37(0)	484(20)	1138(0)	38(0)	482(17)	1125(0)	38(0)	501(17)	1149(0)
39(0)	493(3)	1156(0)	41(0)	497(0)	1154(0)	41(0)	519(0)	1174(0)
39(1)	506(0)	1205(9)	46(0)	500(4)	1181(8)	43(1)	520(8)	1209(7)
46(0)	509(0)	1210(3)	49(1)	508(0)	1197(0)	50(0)	522(0)	1221(1)
49(1)	519(3)	1219(0)	53(1)	508(17)	1209(3)	51(0)	528(6)	1225(1)
64(0)	520(0)	1221(2)	62(0)	512(17)	1213(2)	66(0)	530(26)	1231(2)
68(0)	522(26)	1228(0)	66(0)	516(0)	1216(1)	71(0)	532(0)	1236(0)

73(0)	527(22)	1257(6)	72(0)	532(27)	1264(7)	77(0)	546(19)	1288(6)
79(0)	539(2)	1291(5)	73(0)	538(4)	1314(4)	80(0)	555(0)	1335(3)
81(0)	556(3)	1320(2)	78(0)	556(35)	1332(3)	82(0)	569(49)	1344(4)
83(0)	561(38)	1379(0)	79(0)	570(22)	1372(0)	85(0)	579(12)	1396(0)
89(0)	579(1)	1402(0)	83(0)	576(52)	1391(0)	89(0)	599(50)	1419(0)
92(1)	579(136)	1422(0)	84(1)	580(77)	1408(0)	93(0)	601(102)	1438(0)
96(1)	586(5)	1459(26)	91(1)	593(2)	1435(8)	99(0)	608(12)	1473(26)
102(0)	602(6)	1465(11)	98(0)	606(3)	1453(9)	104(0)	622(51)	1480(13)
121(0)	606(15)	1497(16)	116(0)	608(39)	1477(1)	124(0)	632(8)	1520(2)
130(0)	622(219)	1507(1)	138(2)	618(117)	1487(8)	139(0)	636(216)	1523(15)
146(1)	626(107)	1519(8)	147(0)	624(195)	1490(5)	151(0)	639(105)	1530(8)
147(0)	629(120)	1520(4)	156(0)	626(110)	1507(2)	154(0)	654(115)	1541(5)
152(0)	637(29)	1527(9)	157(1)	637(28)	1517(11)	157(0)	665(77)	1547(15)
163(0)	653(36)	1547(18)	162(0)	658(37)	1526(8)	170(0)	672(45)	1562(20)
222(1)	695(1)	1556(12)	231(4)	697(2)	1533(9)	241(1)	717(1)	1575(14)
247(2)	712(1)	2012(793)	254(0)	705(1)	2017(691)	253(1)	739(1)	2064(860)
259(2)	746(1)	2048(1714)	269(2)	741(2)	2045(323)	280(1)	763(1)	2100(1723)
280(3)	759(1)	2057(11)	296(3)	764(1)	2048(951)	297(4)	786(0)	2121(479)
313(4)	763(1)	2059(1273)	332(0)	769(4)	2053(1708)	334(1)	790(2)	2122(38)
329(0)	787(4)	2061(331)	345(3)	776(0)	2056(553)	341(0)	800(5)	2122(1503)
340(1)	794(0)	2064(1379)	354(0)	780(6)	2060(1006)	357(0)	814(6)	2123(1320)
340(0)	795(6)	2110(1534)	356(1)	798(3)	2109(1388)	364(1)	817(0)	2172(1482)
352(4)	807(2)	2114(322)	374(3)	798(2)	2114(296)	369(6)	831(3)	2175(323)
358(3)	809(4)	3130(3)	377(2)	806(3)	3108(28)	383(2)	832(1)	3153(11)
368(1)	819(1)	3133(1)	385(0)	809(0)	3108(18)	393(1)	842(0)	3155(6)
391(4)	828(2)	3135(7)	404(16)	827(3)	3109(21)	413(3)	850(2)	3158(6)
405(2)	927(1)	3137(3)	413(4)	905(0)	3110(0)	428(0)	949(0)	3160(4)
407(0)	930(7)	3138(4)	413(1)	916(6)	3117(10)	429(1)	954(2)	3162(11)
413(0)	932(0)	3139(6)	415(1)	919(2)	3117(13)	431(0)	959(5)	3165(0)
422(2)	948(0)	3159(2)	425(3)	936(2)	3136(17)	438(2)	964(0)	3180(4)
425(2)	956(4)	3160(2)	428(0)	936(3)	3139(12)	441(0)	977(3)	3180(4)
429(0)	961(4)	3216(1)	444(1)	944(1)	3202(1)	449(1)	980(6)	3238(2)
460(1)	968(1)	3217(0)	460(2)	953(2)	3203(3)	470(1)	994(2)	3240(1)
463(2)	969(1)	3218(3)	465(0)	956(5)	3206(8)	474(2)	994(0)	3241(4)
464(1)	973(1)	3219(3)	469(1)	973(0)	3208(11)	480(0)	997(0)	3242(5)
466(0)	983(0)	3246(1)	470(0)	977(0)	3230(2)	484(0)	999(3)	3270(1)
473(0)	1004(1)	3248(0)	476(1)	994(1)	3234(5)	485(0)	1018(1)	3271(2)

Table S51: Harmonic vibrational frequencies (in cm^{-1}) and infrared intensities (in parentheses in km/mol) for the structure **3-8T-5**.

B3LYP			M06L			wB97XD		
36(0)	464(32)	1012(2)	17(1)	469(1)	1004(1)	38(0)	479(57)	1028(2)
38(0)	465(1)	1144(0)	35(0)	469(0)	1141(0)	40(0)	484(0)	1159(0)

42(0)	465(0)	1147(1)	38(0)	474(24)	1149(1)	48(0)	485(0)	1164(1)
48(0)	479(25)	1207(1)	46(1)	478(11)	1208(3)	51(0)	503(25)	1221(1)
51(0)	483(20)	1217(6)	47(0)	484(0)	1209(4)	55(0)	507(37)	1225(5)
55(1)	491(18)	1222(4)	51(1)	507(2)	1210(6)	60(0)	515(9)	1232(0)
71(0)	510(0)	1226(0)	66(0)	508(19)	1213(0)	73(0)	524(0)	1232(3)
72(0)	510(0)	1230(0)	67(0)	509(22)	1216(1)	75(0)	524(0)	1237(0)
75(0)	520(12)	1256(7)	70(0)	509(4)	1274(10)	79(0)	529(13)	1296(8)
80(0)	521(1)	1268(13)	73(0)	517(1)	1294(12)	81(0)	530(36)	1312(12)
85(0)	522(32)	1312(0)	76(0)	529(13)	1333(0)	88(0)	537(0)	1341(0)
85(0)	532(1)	1389(0)	81(0)	533(2)	1385(1)	88(0)	546(1)	1406(0)
87(0)	557(11)	1397(0)	81(0)	557(9)	1394(0)	90(0)	584(20)	1420(0)
88(0)	578(46)	1405(0)	85(0)	577(15)	1399(0)	93(0)	599(30)	1423(0)
94(1)	579(83)	1466(17)	90(1)	579(107)	1451(34)	99(0)	600(121)	1485(17)
100(0)	590(18)	1493(37)	92(1)	583(13)	1482(19)	104(0)	612(16)	1515(35)
116(0)	591(15)	1499(11)	105(0)	599(29)	1487(3)	119(0)	615(23)	1523(10)
120(0)	597(21)	1514(0)	111(0)	603(11)	1498(0)	124(0)	619(48)	1534(0)
129(0)	624(249)	1517(4)	119(1)	624(218)	1504(4)	136(0)	640(208)	1538(6)
151(1)	628(106)	1525(30)	149(0)	628(133)	1516(11)	160(1)	645(102)	1545(16)
157(0)	633(58)	1526(10)	151(1)	636(55)	1520(5)	161(0)	662(70)	1547(40)
166(0)	640(22)	1528(0)	161(0)	640(12)	1529(21)	173(0)	667(85)	1550(0)
223(3)	678(2)	1533(14)	223(2)	666(3)	1553(15)	242(4)	695(1)	1555(15)
242(1)	704(2)	2023(1303)	243(4)	714(1)	2001(914)	260(0)	728(3)	2087(1406)
248(0)	716(0)	2053(115)	253(3)	723(0)	2043(313)	266(0)	740(0)	2116(745)
267(0)	730(0)	2055(1129)	254(2)	743(4)	2045(897)	273(0)	751(0)	2118(10)
282(0)	751(8)	2057(606)	269(0)	756(8)	2055(696)	326(1)	769(11)	2118(1520)
334(0)	788(5)	2059(1369)	325(2)	783(2)	2058(1809)	347(7)	803(3)	2119(1315)
340(0)	800(0)	2091(1297)	346(0)	791(0)	2060(744)	354(0)	821(9)	2155(1257)
341(1)	802(6)	2106(1407)	348(1)	798(3)	2108(1301)	356(12)	825(7)	2168(1347)
346(0)	805(7)	2112(250)	353(0)	800(5)	2113(329)	362(12)	825(0)	2173(253)
353(4)	807(0)	3126(5)	365(2)	808(9)	3105(24)	365(0)	831(0)	3150(13)
353(0)	822(3)	3130(2)	374(3)	813(4)	3105(27)	377(1)	845(2)	3155(4)
364(14)	825(2)	3132(9)	387(6)	824(2)	3107(21)	385(1)	847(2)	3155(11)
384(6)	876(14)	3135(5)	404(1)	829(17)	3109(7)	398(6)	896(13)	3156(4)
390(4)	880(18)	3136(1)	408(2)	866(3)	3115(11)	413(7)	899(19)	3157(7)
402(4)	936(0)	3136(7)	411(0)	922(2)	3123(14)	427(0)	956(1)	3158(1)
406(2)	948(2)	3137(2)	415(2)	925(2)	3131(9)	428(0)	967(1)	3159(17)
408(0)	950(0)	3139(9)	424(5)	938(2)	3140(4)	435(0)	970(1)	3160(0)
417(4)	961(4)	3212(2)	427(0)	940(0)	3201(3)	436(6)	982(4)	3235(2)
426(1)	962(2)	3215(4)	429(2)	949(2)	3203(4)	438(0)	985(4)	3238(5)
429(0)	964(1)	3217(1)	440(10)	962(0)	3206(10)	440(3)	988(1)	3239(1)
433(29)	969(0)	3218(0)	452(11)	963(0)	3214(8)	450(28)	996(0)	3242(5)
461(15)	982(0)	3219(2)	463(9)	968(0)	3227(1)	472(0)	998(0)	3244(0)
462(15)	1006(1)	3219(4)	464(5)	996(0)	3233(7)	475(1)	1020(1)	3246(3)

Table S52: Harmonic vibrational frequencies (in cm^{-1}) and infrared intensities (in parentheses in km/mol) for the structure **3-8T-6**.

B3LYP			M06L			wB97XD		
-33(0)	469(13)	1010(1)	-37(0)	470(0)	1003(0)	-25(0)	485(0)	1024(1)
37(0)	477(16)	1141(0)	37(0)	478(8)	1137(0)	37(0)	502(8)	1156(0)
37(0)	485(4)	1146(0)	41(0)	484(1)	1143(0)	39(0)	513(2)	1163(0)
39(0)	508(0)	1207(2)	41(0)	505(0)	1199(4)	41(0)	521(0)	1221(2)
47(0)	510(0)	1215(6)	48(0)	508(22)	1204(1)	49(0)	522(0)	1222(5)
47(0)	516(11)	1220(6)	50(0)	508(0)	1208(3)	51(0)	528(2)	1228(7)
63(0)	520(21)	1222(0)	61(0)	508(23)	1209(4)	65(0)	529(35)	1228(0)
67(0)	521(37)	1225(0)	64(0)	523(0)	1212(0)	70(0)	531(35)	1232(0)
72(0)	523(1)	1262(11)	71(0)	528(33)	1279(11)	76(0)	535(3)	1304(11)
77(0)	529(10)	1273(9)	73(0)	530(14)	1295(9)	77(0)	544(9)	1318(6)
79(0)	535(20)	1314(3)	75(0)	538(21)	1330(3)	81(0)	551(23)	1342(4)
84(0)	553(10)	1386(0)	82(0)	562(19)	1379(0)	85(0)	577(14)	1403(0)
88(0)	578(38)	1395(0)	84(0)	575(0)	1387(0)	88(0)	599(4)	1416(0)
88(1)	579(63)	1401(0)	85(0)	578(17)	1395(0)	90(0)	600(136)	1424(0)
94(0)	583(44)	1469(60)	92(1)	579(116)	1465(22)	96(0)	603(25)	1490(64)
98(0)	584(1)	1489(25)	97(0)	589(3)	1481(17)	100(0)	606(28)	1511(26)
118(0)	594(41)	1498(11)	113(0)	600(39)	1489(4)	119(0)	616(56)	1523(8)
122(0)	610(66)	1512(0)	118(0)	611(119)	1496(0)	124(0)	630(131)	1533(0)
148(0)	624(202)	1514(0)	144(0)	623(152)	1499(1)	150(0)	640(130)	1537(0)
155(0)	628(98)	1521(27)	150(0)	628(113)	1505(32)	158(0)	647(79)	1540(46)
159(0)	635(52)	1522(15)	158(0)	637(24)	1509(10)	165(0)	667(39)	1543(11)
160(0)	638(43)	1525(8)	163(0)	640(50)	1512(8)	169(0)	671(121)	1543(17)
235(0)	656(4)	1527(3)	245(2)	647(7)	1520(4)	253(0)	674(5)	1549(6)
236(0)	708(0)	2022(794)	249(0)	709(0)	2018(755)	255(0)	735(0)	2079(887)
249(1)	736(1)	2055(20)	259(2)	723(2)	2044(11)	273(1)	758(3)	2117(1048)
271(0)	737(3)	2056(1260)	273(0)	739(4)	2045(1248)	276(0)	762(5)	2119(12)
310(0)	752(1)	2059(682)	315(2)	753(0)	2055(453)	320(0)	776(0)	2121(1348)
334(0)	793(3)	2062(1575)	343(1)	789(0)	2058(1577)	345(1)	804(1)	2121(1374)
335(0)	796(0)	2065(1067)	350(0)	793(3)	2065(1082)	348(0)	819(10)	2126(1163)
338(0)	798(8)	2107(1491)	352(0)	797(12)	2108(1344)	355(0)	823(0)	2170(1428)
339(0)	803(8)	2113(326)	354(1)	798(1)	2113(303)	362(0)	824(9)	2174(338)
352(5)	804(1)	3128(4)	374(3)	803(11)	3104(24)	380(1)	831(0)	3151(11)
369(0)	818(3)	3130(2)	385(1)	814(3)	3105(30)	383(2)	842(2)	3152(12)
395(3)	823(4)	3132(10)	401(0)	822(3)	3106(26)	413(1)	846(3)	3156(5)
403(0)	894(2)	3134(5)	408(1)	873(2)	3108(0)	416(0)	914(2)	3157(4)
403(0)	901(6)	3135(4)	410(0)	885(5)	3111(24)	424(0)	921(5)	3159(3)
408(4)	934(0)	3136(3)	419(3)	920(0)	3114(12)	427(0)	951(0)	3160(11)
408(0)	946(2)	3137(5)	424(4)	922(5)	3115(11)	435(0)	964(2)	3160(9)
424(3)	949(1)	3139(6)	427(0)	936(1)	3116(5)	436(3)	970(3)	3162(0)
429(0)	959(4)	3214(1)	431(2)	939(1)	3200(2)	438(0)	980(6)	3237(2)
449(0)	960(2)	3214(1)	450(1)	943(0)	3202(3)	460(0)	981(3)	3237(2)

461(3)	962(0)	3216(3)	461(5)	953(0)	3205(9)	471(5)	986(0)	3240(4)
462(2)	964(1)	3216(4)	463(5)	962(0)	3206(12)	474(5)	989(1)	3240(6)
465(1)	985(1)	3228(3)	466(6)	975(1)	3214(1)	482(10)	999(0)	3253(4)
465(0)	1004(1)	3229(0)	469(1)	995(1)	3216(12)	484(0)	1017(1)	3253(0)

Table S53: Harmonic vibrational frequencies (in cm^{-1}) and infrared intensities (in parentheses in km/mol) for the structure **3-8S-7**.

B3LYP			M06L			wB97XD		
28(0)	477(11)	1043(2)	19(0)	479(4)	1052(2)	28(0)	494(11)	1075(4)
30(0)	479(12)	1143(0)	32(0)	482(7)	1142(0)	32(0)	504(8)	1159(0)
39(0)	493(5)	1164(0)	40(0)	492(1)	1164(0)	42(0)	505(1)	1183(0)
43(0)	497(1)	1211(15)	48(0)	503(5)	1206(6)	50(0)	514(8)	1227(1)
47(0)	505(4)	1215(1)	49(0)	507(6)	1209(1)	51(0)	521(6)	1230(6)
65(0)	511(0)	1228(1)	63(0)	510(1)	1218(0)	65(0)	525(1)	1238(2)
72(0)	517(10)	1231(0)	68(0)	510(31)	1222(5)	72(0)	529(23)	1240(3)
74(0)	520(22)	1242(1)	70(0)	519(2)	1230(1)	76(0)	530(10)	1250(2)
78(0)	526(2)	1262(2)	72(0)	530(2)	1268(6)	79(0)	540(2)	1287(5)
78(0)	535(30)	1280(15)	75(0)	535(17)	1298(16)	80(0)	550(29)	1323(12)
82(0)	547(4)	1318(5)	77(1)	552(13)	1333(4)	85(1)	567(5)	1343(6)
86(0)	562(110)	1388(2)	84(0)	565(98)	1387(2)	88(0)	581(118)	1408(1)
87(0)	569(13)	1401(2)	84(0)	574(6)	1398(2)	90(0)	593(23)	1424(1)
90(0)	579(66)	1441(9)	87(1)	578(30)	1418(7)	92(0)	599(65)	1450(9)
94(0)	581(43)	1468(12)	91(0)	579(96)	1446(13)	94(0)	600(59)	1479(16)
96(1)	589(19)	1476(4)	100(0)	590(15)	1471(4)	104(0)	610(64)	1502(3)
104(0)	594(35)	1492(30)	107(1)	598(24)	1486(21)	110(1)	611(14)	1516(31)
106(0)	599(36)	1501(12)	121(0)	605(81)	1495(5)	115(0)	620(28)	1529(5)
126(0)	623(100)	1518(6)	122(1)	624(82)	1505(5)	129(0)	639(140)	1538(8)
129(0)	629(56)	1528(13)	130(0)	631(87)	1518(11)	135(0)	655(26)	1548(17)
159(0)	635(72)	1529(7)	156(0)	636(32)	1522(6)	164(0)	665(100)	1554(14)
183(1)	660(50)	1690(8)	185(3)	667(45)	1696(8)	193(2)	683(41)	1739(8)
190(1)	696(1)	1711(4)	194(1)	697(6)	1719(3)	202(1)	709(3)	1755(4)
204(1)	700(30)	1929(427)	210(0)	708(24)	1943(348)	217(2)	720(29)	1973(510)
231(1)	712(1)	2035(560)	242(1)	718(3)	2024(558)	245(3)	740(1)	2099(576)
253(4)	743(2)	2054(411)	261(3)	746(4)	2042(469)	272(2)	762(6)	2117(610)
286(3)	761(1)	2061(789)	287(3)	767(0)	2056(437)	295(4)	771(0)	2120(898)
328(1)	762(0)	2064(1412)	314(2)	769(2)	2058(1362)	339(1)	780(0)	2127(1331)
339(0)	772(7)	2069(1437)	353(1)	783(4)	2062(1515)	353(0)	802(10)	2134(1565)
341(1)	803(3)	2107(1311)	356(0)	799(4)	2103(1096)	362(2)	826(2)	2170(1231)
346(1)	810(0)	2112(443)	361(2)	806(0)	2111(589)	369(0)	832(2)	2175(482)
364(12)	813(1)	2788(6)	372(13)	812(3)	2781(4)	376(13)	836(0)	2766(8)
382(2)	817(5)	3125(5)	390(9)	817(5)	3089(11)	398(1)	840(2)	3149(5)
385(7)	840(3)	3128(1)	392(1)	840(3)	3104(18)	404(3)	858(3)	3152(18)
398(3)	932(0)	3133(14)	405(1)	899(8)	3110(42)	413(1)	948(1)	3156(6)
402(2)	935(12)	3136(6)	410(0)	910(76)	3116(16)	420(1)	952(5)	3157(6)

406(1)	943(93)	3136(5)	416(5)	926(1)	3120(17)	422(5)	961(98)	3158(10)
412(1)	949(2)	3159(0)	421(2)	930(4)	3146(16)	428(2)	969(3)	3185(3)
420(4)	956(2)	3160(3)	425(2)	939(0)	3152(5)	435(1)	973(2)	3186(3)
426(1)	958(1)	3162(3)	430(2)	941(0)	3152(4)	445(7)	980(5)	3193(3)
433(2)	962(1)	3215(2)	445(1)	955(2)	3204(4)	453(0)	981(1)	3237(3)
440(1)	967(5)	3217(3)	452(5)	959(2)	3209(5)	458(3)	988(2)	3241(2)
455(1)	979(2)	3218(3)	459(4)	975(2)	3210(10)	464(1)	997(4)	3241(5)
462(5)	1004(1)	3249(1)	464(5)	993(1)	3243(7)	474(5)	1016(2)	3278(2)
466(0)	1010(2)	3252(3)	470(0)	1001(1)	3249(5)	486(0)	1022(1)	3280(3)

Table S54: Harmonic vibrational frequencies (in cm^{-1}) and infrared intensities (in parentheses in km/mol) for the structure **3-8T-8**.

B3LYP			M06L			wB97XD		
25(0)	462(4)	1022(1)	34(0)	464(5)	1010(1)	29(0)	474(5)	1031(1)
31(0)	466(0)	1145(1)	38(0)	470(0)	1143(0)	36(0)	484(2)	1160(1)
37(0)	474(4)	1166(0)	39(0)	474(4)	1164(0)	39(0)	487(3)	1184(0)
41(0)	479(11)	1210(16)	49(0)	479(5)	1202(6)	48(0)	492(2)	1229(6)
46(0)	480(1)	1217(0)	53(0)	480(3)	1210(0)	51(0)	500(9)	1230(1)
61(0)	489(16)	1226(1)	63(1)	490(8)	1215(4)	61(0)	506(7)	1237(4)
63(0)	497(15)	1230(0)	65(0)	495(3)	1219(2)	67(0)	508(15)	1237(3)
68(0)	499(1)	1243(1)	70(0)	498(13)	1230(1)	69(0)	510(5)	1251(1)
70(0)	500(7)	1260(3)	72(0)	508(22)	1265(7)	74(0)	515(13)	1286(6)
74(0)	511(0)	1284(14)	74(0)	510(16)	1300(14)	78(0)	524(1)	1325(11)
77(1)	515(4)	1318(4)	77(1)	510(1)	1331(4)	80(1)	527(14)	1343(5)
78(0)	521(24)	1383(0)	79(1)	520(20)	1377(2)	81(0)	532(19)	1402(1)
81(0)	525(1)	1400(0)	84(0)	530(2)	1393(0)	85(0)	539(1)	1421(1)
85(0)	541(35)	1440(9)	85(0)	541(27)	1418(8)	88(0)	558(45)	1447(10)
90(0)	567(1)	1468(10)	88(0)	573(1)	1446(13)	91(0)	588(2)	1476(16)
93(0)	579(63)	1471(9)	98(0)	579(65)	1452(5)	96(0)	599(17)	1490(8)
97(0)	582(32)	1493(23)	107(1)	591(20)	1485(16)	104(0)	601(102)	1517(23)
100(0)	591(24)	1512(7)	114(0)	594(16)	1494(2)	105(1)	607(45)	1530(6)
122(0)	596(3)	1518(9)	120(0)	602(20)	1505(6)	125(0)	614(5)	1538(9)
125(0)	605(102)	1527(12)	132(1)	609(125)	1518(9)	128(1)	631(142)	1548(13)
144(1)	628(107)	1529(12)	156(0)	630(117)	1530(12)	154(0)	649(46)	1562(18)
157(1)	636(71)	1690(10)	164(3)	636(39)	1695(10)	163(0)	664(94)	1739(9)
179(1)	698(14)	1711(6)	187(1)	702(3)	1719(5)	192(2)	708(3)	1755(6)
184(1)	699(3)	1961(493)	197(3)	703(6)	1954(349)	199(3)	715(28)	2006(642)
216(0)	704(21)	2022(649)	227(1)	710(24)	2008(683)	231(0)	727(7)	2077(799)
244(2)	723(4)	2053(749)	252(3)	733(1)	2042(518)	266(1)	750(2)	2116(571)
280(1)	748(15)	2053(603)	282(2)	751(18)	2045(705)	288(2)	765(18)	2116(990)
290(2)	761(0)	2060(1200)	301(2)	764(0)	2056(1168)	301(1)	775(1)	2119(1238)
321(0)	762(0)	2073(1222)	315(1)	766(0)	2059(1234)	329(1)	775(4)	2136(1195)
329(1)	804(4)	2106(1450)	328(1)	793(5)	2099(1118)	336(1)	826(2)	2169(1359)

336(0)	810(0)	2111(422)	351(1)	806(0)	2110(637)	348(0)	832(3)	2172(440)
340(1)	813(1)	3009(3)	354(0)	810(3)	2976(3)	352(1)	836(0)	3016(4)
341(1)	815(5)	3120(7)	358(1)	816(4)	3095(16)	366(1)	839(2)	3142(10)
351(1)	841(3)	3127(8)	358(1)	842(2)	3099(21)	370(1)	859(2)	3152(19)
368(5)	931(0)	3129(2)	379(1)	898(6)	3104(15)	390(10)	945(3)	3153(6)
385(6)	935(2)	3133(13)	388(9)	907(84)	3109(43)	399(4)	948(8)	3157(3)
396(5)	941(106)	3136(8)	402(1)	926(1)	3119(18)	412(0)	953(99)	3157(12)
403(1)	948(2)	3159(1)	409(4)	936(2)	3145(15)	422(9)	969(2)	3184(3)
408(17)	956(2)	3161(3)	413(9)	939(0)	3149(4)	426(4)	973(1)	3185(3)
410(8)	957(1)	3168(3)	417(30)	949(6)	3157(5)	430(33)	980(2)	3201(4)
412(19)	963(2)	3200(4)	421(4)	955(1)	3193(8)	435(3)	981(3)	3225(4)
424(4)	973(6)	3215(2)	425(8)	961(2)	3203(4)	439(16)	995(3)	3237(3)
429(3)	979(5)	3216(4)	426(1)	975(4)	3209(10)	448(6)	997(6)	3241(5)
436(7)	985(0)	3248(2)	439(2)	980(0)	3242(6)	456(1)	1007(4)	3278(3)
445(12)	1006(0)	3251(3)	446(8)	996(0)	3245(5)	464(18)	1018(0)	3280(2)

Table S55. Harmonic vibrational frequencies (in cm-1) ν(CO) and infrared intensities (in parentheses, in km/mol) for the (hericene)$Fe_m(CO)_n$ (m = 1, n = 3; m = 2, n = 6, 5; m = 3, m = 9, 8) derivatives. Bridging ν(CO) frequencies are in **bold** type.

**structures**	ν(CO)/cm^{-1}
**1-3S-1(*C_s*)**	2045(629),2052(895),2108(794)
**2-6S-1(*C_s*)**	2044(254),2046(978),2054(438),2056(1371),2107(1272),2111(348)
**2-6S-2(*C_{2v}*)**	2046(0),2049(1202),2053(600),2056(1082),2105(1490),2114(240)
**2-6S-3(*C_{2v}*)**	2032(0),2049(1288),2063(46),2072(1178),2103(578),2123(1314)
**2-5S-1(*C_1*)**	2027(692),2046(676),2055(1224),2064(910),2110(710)
**2-5S-2(*C_1*)**	2029(735),2046(740),2055(933),2065(1087),2110(670)
**2-5S-3(*C_s*)**	**1902(378)**,2022(2),2052(956),2063(1084),2095(1547)
**2-5S-4(*C_1*)**	2018(931),2046(628),2053(762),2068(1333),2110(729)
**2-5S-5(*C_s*)**	2015(670),2044(657),2053(1208),2065(697),2108(768)
**2-5T-6(*C_1*)**	2012(791),2045(615),2053(1967),2059(396),2109(764)
**2-5T-7(*C_1*)**	2003(1037),2045(617),2054(686),2060(1480),2109(761)
**2-5T-8(*C_1*)**	2014(844),2048(633,2054(498),2057(1796),2110(761)
**2-5T-9(*C_1*)**	2017(818),2044(630),2053(1346),2065(899),2109(730)
**2-5S-10(*C_1*)**	**1942(475)**,2015(563),2025(898),2060(1161),2095(949)
**2-5T-11(*C_1*)**	**1955(380)**,2005(711),2042(938),2058(1180),2098(895)
**2-5T-12(*C_1*)**	2000(472),2031(982),2049(688),2064(1057),2108(1020)
**3-9S-1(*C_{3h}*)**	2043(0),2043(0),2044(1825),2054(0),2058(1358),2058(1358) 2108(1262),2108(1263),2115(0)
**3-9S-2(*C_1*)**	2037(57),2046(513),2050(855),2057(1267),2064(240) 2065(998),2104(990),2108(1266),2123(695)
**3-8S-1(*C_1*)**	**1900(373)**,2025(8),2047(387),2055(1146),2058(876) 2065(1090),2095(2022),2113(475)
**3-8S-2(*C_1*)**	2019(804),2044(30),2045(1205),2055(579),2057(1491) 2069(1140),2108(1275),2113(279)
**3-8T-3(*C_1*)**	**1927(409)**,2001(47),2031(1033),2046(664),2058(927) 2061(1452),2091(1369),2112(662)
**3-8S-4(*C_s*)**	2017(691),2045(322),2047(951),2053(1708),2055(553) 2059(1006),2109(1388),2114(295)
**3-8T-5(*C_1*)**	2001(913),2043(312),2045(896),2055(696),2057(1809) 2059(744),2107(1300),2112(328)
**3-8T-6(*C_s*)**	2018(755),2044(10),2045(1248),2055(452),2058(1576) 2065(1081),2108(1344),2113(302)
**3-8S-7(*C_1*)**	**1943(348)**,2023(557),2042(469),2055(437),2058(1361) 2062(1514),2102(1095),2110(589)
**3-8T-8(*C_1*)**	**1953(349)**,2008(683),2042(517),2044(704),2055(1168) 2059(1234),2098(1117),2109(636)

Table S56. Natural charges, Wiberg bond indices, Fe-Fe distances, and formal Fe-Fe bond orders for the (hericene)$Fe_m(CO)_n$ (m = 1, n = 3; m = 2, n = 6, 5; m = 3, m = 9, 8) derivatives using the M06-L method.

M06-L	Natural charge on Fe1/Fe2/Fe3	Wiberg bond index	Fe1-Fe2 distance (Å)	Formal Fe-Fe bond order
**(3-9S-2)**	-1.500/-1.497/-1.548	0.01	4.096	0
**(3-8S-1**)	-1.199/–1.175/-1.556	0.22	2.708	0
**(3-8T-3**)	-0.503/-1.390/-1.557	0.24	2.557	1
**(3-8S-7**)	-0.893/-1.197/-1.565	0.54	2.376	1
**(3-8T-8**)	-0.532/-1.142/-1.566	0.28	2.451	1
**(2-6S-3**)	-1.515/-1.515	0.01	4.234	0
**(2-5S-3**)	-1.196/-1.196	0.22	2.711	1
**(2-5S-10**)	-0.788/-1.265	0.47	2.313	2
**(2-5T-11**)	-1.156/-0.538	0.28	2.451	1
**(2-5T-12**)	-1.511/-0.328	0.04	3.648	0

Complete Gaussian 09 reference (Reference 24)

M. J. Frisch; G. W. Trucks; H. B. Schlegel; G. E. Scuseria; M. A. Robb; J. R. Cheeseman; G. Scalmani; V. Barone; B. Mennucci; G. A. Petersson; H. Nakatsuji; M. Caricato; X. Li; H. P. Hratchian; A. F. Izmaylov; J. Bloino; G. Zheng;J. L. Sonnenberg; M. Hada; M. Ehara; K. Toyota; R. Fukuda; J. Hasegawa; M. Ishida; T. Nakajima; Y. Honda; O. Kitao; H. Nakai; T. Vreven; J. A. Montgomery; Jr.; J. E. Peralta; F. Ogliaro; M. Bearpark; J. J. Heyd; E. Brothers; K. N. Kudin; V. N. Staroverov; R. Kobayashi; J.Normand; K. Raghavachari; A. Rendell; J. C. Burant; S. S. Iyengar; J. Tomasi; M.Fessi; N. Rega; J. M. Millam; M. Klene; J. E. Knox; J. B. Cross; V. Bakken; C.Adamo; J. Jaramillo; R. Gomperts; R. E. Stratmann; O. Yazyev; A. J. Austin; R.Cammi; C. Pomelli; J. W. Ochterski; R. L. Martin; K. Morokuma; V. G. Zakrzewski; G. A. Voth; P. Salvador; J. J. Dannenberg; S. Dapprich; A. D. Daniels; O. Farkas; J. B. Foresman; J. V. Ortiz; J. Cioslowski; and D. J. Fox, J. A. Gaussian 09, Revision A.02, Gaussian, Inc., Wallingford CT, 2009.
